# Supplementary material for: Vulnerabilities in child well-being among primary school children: a cross-sectional study in Bradford, UK
Source: BMJ Open. 2022 Jun 30;12(6):e049416. doi: 10.1136/bmjopen-2021-049416 (PMC9247690; doi:10.1136/bmjopen-2021-049416)
Supplement: Supplementary data [file bmjopen-2021-049416supp001.pdf]

Supplementary Materials: Vulnerabilities in child wellbeing among primary school children: a cross sectional study in Bradford, UK

1: STROBE Statement—Checklist of items that should be included in reports of ***cross-sectional studies***

	Item No	Recommendation
**Title and abstract**	1	(*a*) Indicate the study's design with a commonly used term in the title or the abstract SEE TITLE
		(*b*) Provide in the abstract an informative and balanced summary of what was done and what was found SEE ABSTRACT
**Introduction**		
Background/rationale	2	Explain the scientific background and rationale for the investigation being reported SEE INTRODUCTION, PAGE 4
Objectives	3	State specific objectives, including any prespecified hypotheses N/A
**Methods**		
Study design	4	Present key elements of study design early in the paper SEE METHODS, PAGES -7
Setting	5	Describe the setting, locations, and relevant dates, including periods of recruitment, exposure, follow-up, and data collection SEE SETTINGS AND PARTICIPANTS, PAGE 5
Participants	6	(*a*) Give the eligibility criteria, and the sources and methods of selection of participants SEE SETTINGS AND PARTICIPANTS, PAGE 5
Variables	7	Clearly define all outcomes, exposures, predictors, potential confounders, and effect modifiers. Give diagnostic criteria, if applicable SEE DESIGN AND PROCEDURES, PAGE 6
Data sources/ measurement	8*	For each variable of interest, give sources of data and details of methods of assessment (measurement). Describe comparability of assessment methods if there is more than one group SEE DESIGN AND PROCEDURES, PAGE 6
Bias	9	Describe any efforts to address potential sources of bias SEE FIGURE 1, PAGE 5
Study size	10	Explain how the study size was arrived at SEE SETTINGS AND PARTICIPANTS, PAGE 5
Quantitative variables	11	Explain how quantitative variables were handled in the analyses. If applicable, describe which groupings were chosen and why SEE DESIGN AND PROCEDURES, PAGE 6
Statistical methods	12	(*a*) Describe all statistical methods, including those used to

		control for confounding
		(*b*) Describe any methods used to examine subgroups and interactions
		(*c*) Explain how missing data were addressed
		(*d*) If applicable, describe analytical methods taking account of sampling strategy
		(*e*) Describe any sensitivity analyses<br>SEE DATA ANALYSIS, PAGE 7
**Results**		
Participants	13*	(a) Report numbers of individuals at each stage of study—eg numbers potentially eligible, examined for eligibility, confirmed eligible, included in the study, completing follow-up, and analysed
		(b) Give reasons for non-participation at each stage
		(c) Consider use of a flow diagram<br>SEE METHODS, FIGURE 1, PAGE 5 AND ALL RESULTS TABLES
Descriptive data	14*	(a) Give characteristics of study participants (eg demographic, clinical, social) and information on exposures and potential confounders<br>SEE TABLE 1, PAGE 8
		(b) Indicate number of participants with missing data for each variable of interest<br>SEE ALL TABLES AND FIGURES
Outcome data	15*	Report numbers of outcome events or summary measures<br>SEE FIGURE 1, PAGE 9
Main results	16	(*a*) Give unadjusted estimates and, if applicable, confounder-adjusted estimates and their precision (eg, 95% confidence interval). Make clear which confounders were adjusted for and why they were included<br>SEE ALL TABLES AND FIGURES
		(*b*) Report category boundaries when continuous variables were categorized<br>N/A
		(*c*) If relevant, consider translating estimates of relative risk into absolute risk for a meaningful time period<br>N/A
Other analyses	17	Report other analyses done—eg analyses of subgroups and interactions, and sensitivity analyses<br>SEE DISCUSSION RE: UNREPORTED ANALYSIS OF IMD, PAGE 18
**Discussion**		
Key results	18	Summarise key results with reference to study objectives<br>SEE STATEMENT OF PRINCIPAL FINDINGS, PAGE 16
Limitations	19	Discuss limitations of the study, taking into account sources of potential bias or imprecision. Discuss both direction and magnitude of any potential bias

		SEE STRENGTHS AND WEAKNESSES OF THE STUDY, PAGE 17
Interpretation	20	Give a cautious overall interpretation of results considering objectives, limitations, multiplicity of analyses, results from similar studies, and other relevant evidence<br>SEE IMPLICATIONS SECTION, PAGE 18
Generalisability	21	Discuss the generalisability (external validity) of the study results<br>SEE STRENGTHS AND WEAKNESSES OF THE STUDY, PAGE 17
**Other information**		
Funding	22	Give the source of funding and the role of the funders for the present study and, if applicable, for the original study on which the present article is based<br>SEE FUNDING STATEMENT

2. Me and My Life wellbeing survey

Me and my life

Please tick the box next to your answer

Please try to answer all the questions. There are no right or wrong answers. We want to know what you think.

Your answers will not be shown to anyone that you know (including mums and dads).

If you do not want to answer a question you can miss it out.

If you need any help, you can ask your teacher or the person who gave you this quiz.

Thank you!

1. Which of these would you most like to eat today?

Pizza 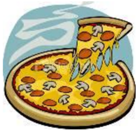 ☐

Curry 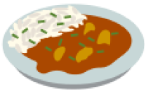 ☐

Pasta with sauce 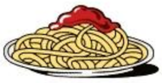 ☐

Sandwich 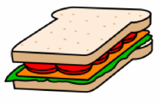 ☐

Where I live

2. How much do you like the home where you live?

I like it a lot 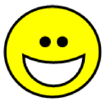 ☐

I like it a bit 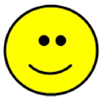 ☐

I don't like it 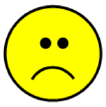 ☐

3. How often do you play in a park?

Very often ☐

Sometimes ☐

Never ☐

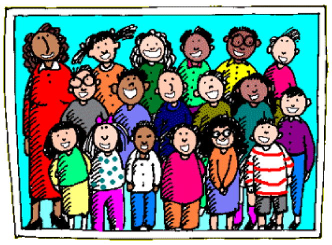

My family and the people I live with

4. How often do you have fun with your family at the weekend?

All of the time ☐

Some of the time ☐

Never ☐

5. How often does your family get along well together?

All of the time ☐

Some of the time ☐

Never ☐

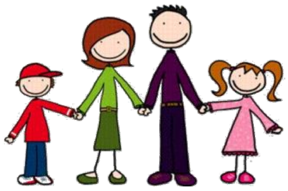

6. How often do you get along with your brothers, sisters and other children you live with?

All of the time ☐

Some of the time ☐

Never ☐

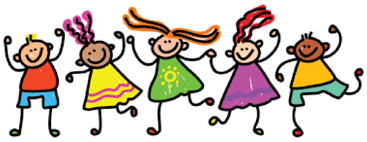

I don't have brothers or sisters or live with other children ☐

7. What languages do you speak with your family at home?

You can tick **more than one** answer if you like.

- English ☐
- Urdu ☐
- Mirpuri/Punjabi ☐
- Slovakian ☐
- Arabic ☐
- Bangla/Bengali ☐
- Hindko ☐
- Pushto ☐
- Other ☐

A bit about me

8. Here is a list of things that some children have.

Do you have each of these things?

		Yes<br>I have it	No<br>I don't have it
Some money to spend on myself	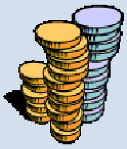	☐	☐
Clothes that you think your friends like	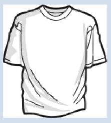	☐	☐
A warm winter coat	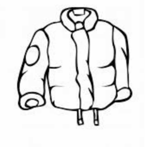	☐	☐
Your own mobile phone	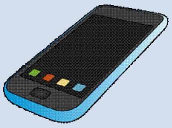	☐	☐
A computer, laptop or tablet with internet at home	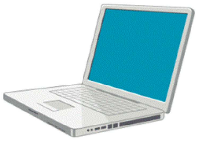	☐	☐
Three meals every day	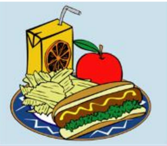	☐	☐

9. Do you have each of these things?

		**Yes** I have it	**No** I don't have it
**Family trips or days out at least once a month, such as to the seaside or a theme park**	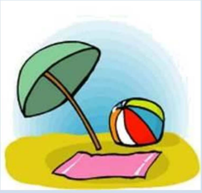	☐	☐
**At least one family holiday away from home each year**	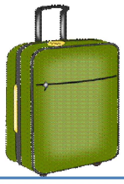	☐	☐

10. Does your home have a garden where you can play?

Yes ☐

No ☐

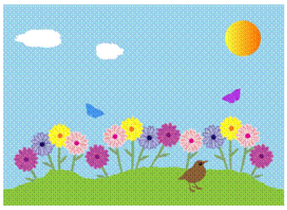

11. Does your family have a car?

Yes ☐

No ☐

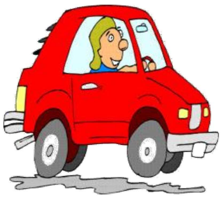

12. Do you have a park near your home where you can play with your friends?

Yes ☐

No ☐

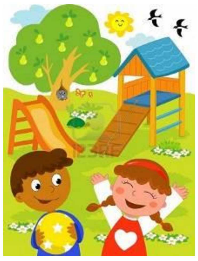

13. How often do you worry about how much money your family has?

All of the time ☐

Some of the time ☐

Never ☐

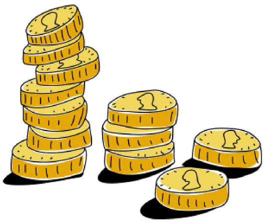

14. How often do you feel happy?

All of the time ☐

Some of the time ☐

Never ☐

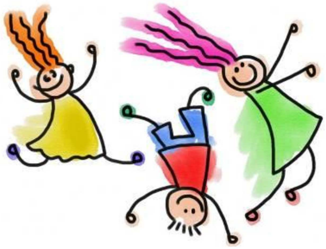

15. How often do you feel sad?

All of the time ☐

Some of the time ☐

Never ☐

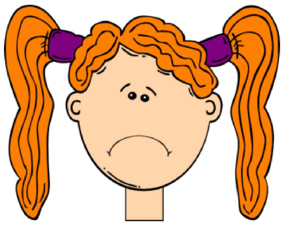

16. How often are you ill or unwell?

All of the time ☐

Some of the time ☐

Never ☐

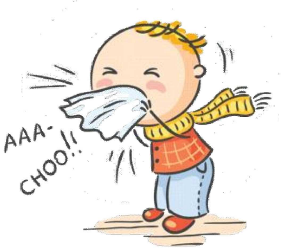

17. How often do you feel healthy?

All of the time ☐

Some of the time ☐

Never ☐

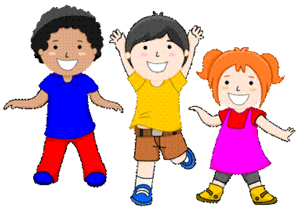

18. What do you do if you are worried about something?

You can tick **more than one** answer if you like.

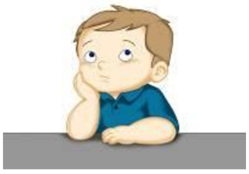

I keep it to myself ☐

I tell a friend ☐

I tell my Mum, Dad or the person who looks after me ☐

I tell a teacher ☐

19. When I find something really hard I can work out what to do next.

All of the time ☐

Some of the time ☐

Never ☐

My friends and other children

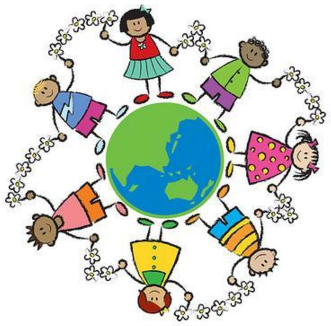

20. How many friends do you have?

Lots ☐

Some ☐

Not many ☐

21. Do you have any best friends?

Yes ☐

No ☐

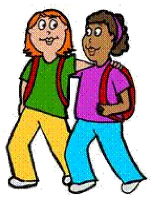

22. How much do you like playing with your friends?

I like it a lot 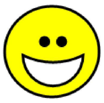 ☐

I like it a bit 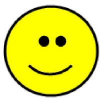 ☐

I don't like it 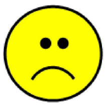 ☐

23. How often do other children bully you?

All of the time ☐

Some of the time ☐

Never ☐

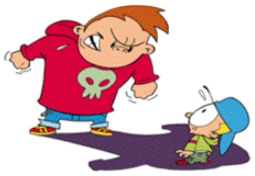

24. How often are you mean to other children at school?

All of the time ☐

Some of the time ☐

Never ☐

25. How often do you feel left out of things by other children?

All of the time ☐

Some of the time ☐

Never ☐

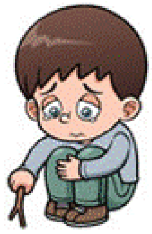

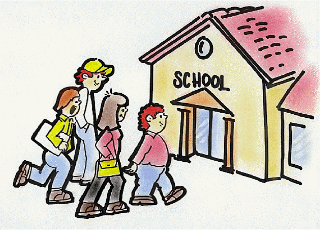

My school

26. How much do you like school?

I like it a lot	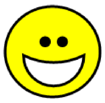	☐
I like it a bit	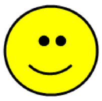	☐
I don't like it	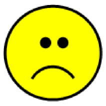	☐

After school and weekend activities

27. Do you go to any of these clubs or activities after school or at weekends?

		Yes	No
**Sports (such as football, cricket, swimming, karate)**	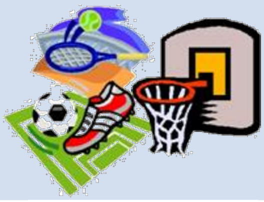	☐	☐
**Music lessons (such as choir, piano lessons)**	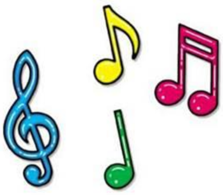	☐	☐
**Dance lessons**	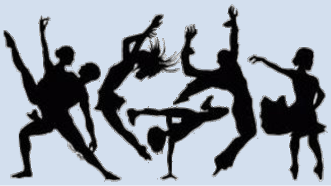	☐	☐
**Any other club (such as art, drama, film, cooking)**		☐	☐

Religion

28. **What is your religion?**

- Christian ☐
- Muslim ☐
- Hindu ☐
- Sikh ☐
- Other religion ☐
- None/No religion ☐
- Don't know ☐

If you are Muslim:

Do you go to Mosque or Madrassa?

- Yes ☐
- No ☐

If yes: How often do you go to Mosque or Madrassa?

- Most days a week ☐
- Some days a week ☐
- Less than once a week ☐

If you are Christian:

Do you go to Church or Sunday school?

Yes ☐

No ☐

If yes: How often do you go to Church or Sunday school?

Most days a week ☐

Some days a week ☐

Less than once a week ☐

If you are Hindu/Sikh:

Do you go to a Temple?

Yes ☐

No ☐

If yes: How often do you go to a Temple?

Most days a week ☐

Some days a week ☐

Less than once a week ☐

If you are another religion:

Do you go to a place of worship?

Yes ☐

No ☐

If yes: How often do you go to a place of worship?

Most days a week ☐

Some days a week ☐

Less than once a week ☐

When I grow up

29. When you grow up, would you like to be:

		Yes, I'd like it a lot	Yes, I'd like it a bit	No, not at all
A cook	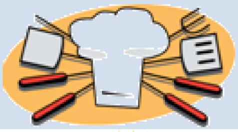	☐	☐	☐
A sports star	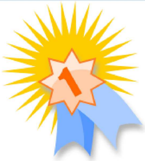	☐	☐	☐
A train driver	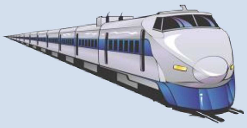	☐	☐	☐
A pop star	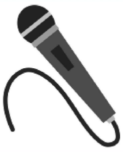	☐	☐	☐
Someone who looks after children at home	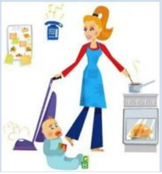	☐	☐	☐
A doctor or a nurse	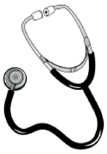	☐	☐	☐
Someone who works in a shop	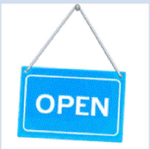	☐	☐	☐
A teacher	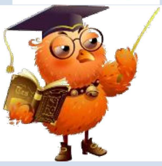	☐	☐	☐
Someone who works with computers	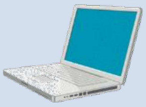	☐	☐	☐

30. What would you most like to be when you grow up?
You can write any job you like!

………

………

31. How much do you like:

		I like it a lot	I like it a bit	I don't like it/ I don't do it
Eating Asian food, such as curry	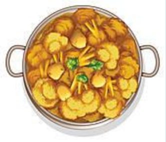	☐	☐	☐
Eating English food, such as fish and chips	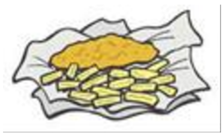	☐	☐	☐
Wearing Asian clothes such as Salwar kameez or kurta	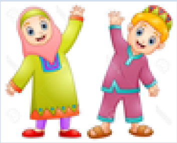	☐	☐	☐
Wearing clothes such as jeans, t-shirts or dresses	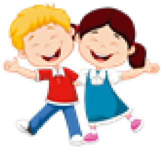	☐	☐	☐

32.

		Yes	No
Do you and your family celebrate Eid?	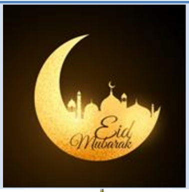	☐	☐
Do you and your family celebrate Christmas?	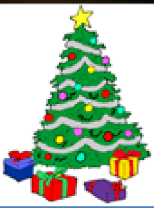	☐	☐

Thank you for your time!

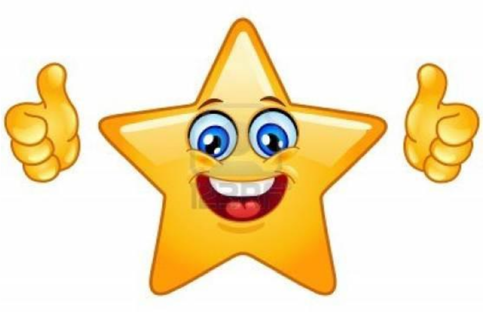

<u>Researcher/Teacher only:</u>
<u>Tick if the child was assisted to complete the questionnaire.</u> ☐

<u>Table S1: Vulnerability across domains of wellbeing</u>

Table S1a: Response to items within 'Home, Family and Family Relationships' domain

How often does your family get along well	Number	Percentage (95% CI)
Never	729	4.8% (4.4%-5.1%)
Some of the time	6,845	44.8% (44.1%-45.6%)
All of the time	7,689	50.4% (49.6%-51.2%)
Missing	378	
Total	15,641	100.0%

Does your home have a garden where you can play?	Number	Percentage (95% CI)
No	2,036	13.2% (12.7%-13.8%)
Yes	13,357	86.8% (86.2%-87.3%)
Missing	248	
Total	15,641	100.0%

Do you have a park near your home where you can play with your friends?	Number	Percentage (95% CI)
No	4,711	30.8% (30.1%-31.5%)
Yes	10,584	69.2% (68.5%-69.9%)
Missing	346	
Total	15,641	100.0%

How often do you play in a park?	Number	Percentage (95% CI)
Never	1,137	7.5% (7.1%-7.9%)
Sometimes	11,701	76.9% (76.2%-77.5%)
Very often	2,386	15.7% (15.1%-16.3%)
Missing	417	
Total	15,641	100.0%

Supplementary Table S1b: Response to items within 'Material Resources' domain

Do you have a warm winter coat?	Number	Percentage (95% CI)
No	1,402	10% (9.5%-10.5%)
Yes	12,594	90% (89.5%-90.5%)
Missing	1,645	
Total	15,641	100.0%

Do you have three meals every day?	Number	Percentage (95% CI)
No	2,243	15.9% (15.3%-16.5%)
Yes	11,891	84.1% (83.5%-84.7%)
Missing	1,507	
Total	15,641	100.0%

Do you have a computer/laptop/tablet with internet at home?	Number	Percentage (95% CI)
No	2,733	19.1% (18.5%-19.8%)
Yes	11,563	80.9% (80.2%-81.5%)
Missing	1,345	
Total	15,641	100.0%

How often do you worry about how much money your family has?	Number	Percentage (95% CI)
Never	5,264	34.1% (33.3%-34.8%)
Some of the time	6,124	39.7% (38.9%-40.4%)
All of the time	4,054	26.3% (25.6%-27%)
Missing	199	
Total	15,641	100.0%

Supplementary Table S1c: Response to items within 'Friends and School' domain

How much do you like your school?	Number	Percentage (95% CI)
Don't like it	2,032	13.1% (12.6%-13.6%)
Like it a bit	4,691	30.2% (29.5%-31%)
Like it a lot	8,797	56.7% (55.9%-57.5%)
Missing	121	
Total	15,641	100.0%

How many friends do you have?	Number	Percentage (95% CI)
Not many	2,257	14.5% (14%-15.1%)
Some	3,647	23.5% (22.8%-24.2%)
Lots	9,623	62% (61.2%-62.7%)
Missing	114	
Total	15,641	100.0%

How often do other children bully you?	Number	Percentage (95% CI)
Never	7,276	47.3% (46.6%-48.1%)
Some of the time	6,413	41.7% (41%-42.5%)
All of the time	1,679	10.9% (10.4%-11.4%)
Missing	273	
Total	15,641	100.0%

How often are you mean to other children at school?	Number	Percentage (95% CI)
Never	10,806	70.9% (70.2%-71.6%)
Some of the time	3,645	23.9% (23.2%-24.6%)
All of the time	794	5.2% (4.9%-5.6%)
Missing	396	
Total	15,641	100.0%

How often do you feel left out by other children?	Number	Percentage (95% CI)
Never	5,598	36.2% (35.5%-37%)
Some of the time	8,129	52.6% (51.8%-53.4%)
All of the time	1,729	11.2% (10.7%-11.7%)
Missing	185	
Total	15,641	100.0%

Supplementary Table S1d: Response to items within 'Subjective Wellbeing' domain

How often do you feel happy?	Number	Percentage (95% CI)
Never	585	3.8% (3.5%-4.1%)
Some of the time	8,306	54.2% (53.5%-55%)
All of the time	6,420	41.9% (41.2%-42.7%)
Missing	330	
Total	15,641	100.0%

How often do you feel sad?	Number	Percentage (95% CI)
Never	3,837	24.9% (24.2%-25.6%)
Some of the time	10,715	69.5% (68.8%-70.2%)
All of the time	863	5.6% (5.2%-6%)
Missing	226	
Total	15,641	100.0%

How often are you ill or unwell?	Number	Percentage (95% CI)
Never	2,216	14.4% (13.8%-14.9%)
Some of the time	11,966	77.6% (76.9%-78.2%)
All of the time	1,247	8.1% (7.7%-8.5%)
Missing	212	
Total	15,641	100.0%

Keep worries to self	Number	Percentage (95% CI)
No	10,655	68.8% (68.1%-69.5%)
Yes	4,828	31.2% (30.5%-31.9%)
Missing	158	
Total	15,641	100.0%

When I find something really hard I can work out what to do next	Number	Percentage (95% CI)
Never	1,299	8.6% (8.1%-9%)
Some of the time	9,429	62.3% (61.5%-63.1%)
All of the time	4,409	29.1% (28.4%-29.9%)
Missing	504	
Total	15,641	100.0%

Table S2: Percentage primary school children with zero, one, or more than one vulnerability in each domain of wellbeing

Home, family, relationships	Number	Percentage (95% CI)
None	8,085	55.9% (55%-56.7%)
One	4,902	33.9% (33.1%-34.6%)
More than one	1,489	10.3% (9.8%-10.8%)
Missing	1,165	
Total	15,641	100.0%

Material resources	Number	Percentage (95% CI)
None	6,331	48.3% (47.5%-49.2%)
One	4,763	36.4% (35.5%-37.2%)
More than one	2,008	15.3% (14.7%-16%)
Missing	2,539	
Total	15,641	100.0%

Friends and school	Number	Percentage (95% CI)
None	5,204	35% (34.3%-35.8%)
One	6,189	41.7% (40.9%-42.5%)
More than one	3,455	23.3% (22.6%-24%)
Missing	793	
Total	15,641	100.0%

Subjective wellbeing	Number	Percentage (95% CI)
None	8,312	57.4% (56.6%-58.2%)
One	4,676	32.3% (31.5%-33.1%)
More than one	1,496	10.3% (9.8%-10.8%)
Missing	1,157	
Total	15,641	100.0%

Supplementary Table S3: Associations of gender with vulnerabilities in child wellbeing domains

Supplementary Table S3a – Home, family, family relations

	Family never get along well together (N = 15,263 non-missing)		No garden where they can play (N = 15,393 non-missing)		No park near home where they can play with friends (N = 15,295 non-missing)		Never plays in park (N = 15,224 non-missing)	
**Gender**	**N**	**Percentage (95% CI)**	**N**	**Percentage (95% CI)**	**N**	**Percentage (95% CI)**	**N**	**Percentage (95% CI)**
Female (N = 7,647)	296	4.0% (3.5%-4.4%)	891	11.8% (11.1%-12.5%)	2225	11.8% (11.1%-12.5%)	456	6.1% (5.6%-6.7%)
Male (N = 7,994)	433	5.6% (5.1%-6.1%)	1145	14.6% (13.8%-15.4%)	2486	14.6% (13.8%-15.4%)	681	8.8% (8.2%-9.4%)

Supplementary Table S3b – Material resources

	No warm winter coat (N = 13,996 non-missing)		Not having three meals a day (N = 14,134 non-missing)		No internet at home (N = 14,296 non-missing)		Worried about money all the time (N = 15,442 non-missing)	
**Gender**	**N**	**Percentage (95% CI)**	**N**	**Percentage (95% CI)**	**N**	**Percentage (95% CI)**	**N**	**Percentage (95% CI)**
Female (N = 7,647)	616	8.9% (8.2%-9.6%)	983	14.0% (13.3%-14.9%)	1383	19.7% (18.8%-20.6%)	1771	23.4% (22.5%-24.4%)
Male (N = 7,994)	786	11.1% (10.4%-11.9%)	1260	17.7% (16.8%-18.6%)	1350	18.6% (17.7%-19.5%)	2283	28.9% (28.0%-30.0%)

Supplementary Table S3c – Friends and school

Gender	Does not like school (N = 15,520 non-missing) N	Percentage (95% CI)	Not many friends (N = 15,527 non-missing) N	Percentage (95% CI)	Bullied some or all of the time (N = 15,368 non-missing) N	Percentage (95% CI)	Mean to others all the time (N = 15,245 non-missing) N	Percentage (95% CI)	Feel left out all the time (N = 15,456 non-missing) N	Percentage (95% CI)
Female (N = 7,647)	606	8.0% (7.4%-8.6%)	1269	16.7% (15.9%-17.6%)	4192	55.8% (54.7%-56.9%)	252	3.4% (3.0%-3.8%)	916	12.1% (11.4%-12.8%)
Male (N = 7,994)	1426	18.0% (17.1%-18.8%)	988	12.5% (11.8%-13.2%)	3900	49.7% (48.6%-50.8%)	542	7.0% (6.4%-7.6%)	813	10.3% (9.7%-11.0%)

Supplementary Table S3d – Subjective wellbeing

Gender	Never happy (N = 15,331 non-missing) N	Percentage (95% CI)	Always sad (N = 15,415 non-missing) N	Percentage (95% CI)	Always ill or unwell (N = 15,429 non-missing) N	Percentage (95% CI)	Keeps worries to self (N = 15,483 non-missing) N	Percentage (95% CI)	Cannot work out what to do when things are hard (N = 15,137 non-missing) N	Percentage (95% CI)
Female (N = 7,647)	179	2.4% (2.1%-2.8%)	412	5.5% (5.0%-6.0%)	581	7.7% (7.1%-8.3%)	2166	28.5% (27.5%-29.6%)	555	7.5% (6.9%-8.1%)
Male (N = 7,994)	406	5.2% (4.7%-5.7%)	451	5.7% (5.2%-6.3%)	666	8.5% (7.9%-9.1%)	2662	33.7% (32.7%-34.8%)	744	9.6% (9.0%-10.3%)

Supplementary Table S3e: Associations of gender for each child wellbeing domain

	Home, family and family relationships (N = 14,476 non-missing)		Material resources (N = 13,102 non-missing)		Friends and school (N = 14,848 non-missing)		Subjective wellbeing (N = 14,484 non-missing)	
**Gender**	**N**	**Percentage (95% CI)**	**N**	**Percentage (95% CI)**	**N**	**Percentage (95% CI)**	**N**	**Percentage (95% CI)**
Female (N = 7,647)	7,117	41.7% (40.6%-42.9%)	6,520	48.8% (47.6%-50.0%)	7,284	65.0% (63.9%-66.1%)	7,148	39.5% (38.4%-40.7%)
Male (N = 7,994)	7,359	46.5% (45.3%-47.6%)	6,582	54.5% (53.3%-55.7%)	7,564	64.9% (63.8%-66.0%)	7,336	45.6% (44.5%-46.7%)

Supplementary Table S4: Associations of ethnicity with vulnerabilities in child wellbeing domains

Supplementary Table S4a – Home, family, family relations

	Family never get along well together (N = 15,263 non-missing)		No garden where they can play (N = 15,393 non-missing)		No park near home where they can play with friends (N = 15,295 non-missing)		Never plays in park (N = 15,224 non-missing)	
**Ethnicity**	**N**	**Percentage (95% CI)**	**N**	**Percentage (95% CI)**	**N**	**Percentage (95% CI)**	**N**	**Percentage (95% CI)**
Pakistani (N = 7,031)	278	4.1% (3.6%-4.6%)	837	12.1% (11.4%-12.9%)	2243	32.7% (31.6%-33.9%)	470	6.9% (6.3%-7.5%)
White British (N = 4,247)	265	6.3% (5.6%-7.1%)	562	13.4% (12.4%-14.5%)	1247	29.9% (28.6%-31.3%)	374	9.0% (8.2%-9.9%)
Mixed (N = 900)	55	6.3% (4.9%-8.1%)	134	15.0% (12.8%-17.5%)	254	28.7% (25.8%-31.8%)	75	8.5% (6.8%-10.5%)
White Other (N = 707)	24	3.5% (2.3%-5.2%)	125	18.0% (15.3%-21%)	188	27.1% (23.9%-30.6%)	46	6.7% (5.1%-8.9%)
Bangladeshi (N = 471)	18	3.9% (2.5%-6.2%)	73	15.8% (12.8%-19.5%)	154	33.6% (29.4%-38.0%)	32	6.9% (4.9%-9.6%)
Indian (N = 357)	17	4.9% (3.0%-7.7%)	64	18.1% (14.4%-22.4%)	121	34.4% (29.6%-39.5%)	25	7.2% (4.9%-10.4%)
Black/ Black British (N = 277)	5	1.8% (0.8%-4.3%)	49	18.0% (13.9%-23%)	62	22.7% (18.1%-28.1%)	19	7.0% (4.5%-10.7%)
Gypsy/ Irish traveller (N = 190)	13	7.3% (4.3%-12.2%)	28	15.2% (10.7%-21.2%)	54	29.2% (23.1%-36.1%)	18	9.9% (6.3%-15.2%)
Other (N = 425)	14	3.4% (2.0%-5.7%)	76	18.1% (14.7%-22.1%)	129	31.5% (27.2%-36.1%)	35	8.5% (6.1%-11.5%)

Supplementary Table S4b – Material resources

Ethnicity	No warm winter coat (N = 13,996 non-missing) N	Percentage (95% CI)	Not having three meals a day (N = 14,134 non-missing) N	Percentage (95% CI)	No internet at home (N = 14,296 non-missing) N	Percentage (95% CI)	Worried about money all the time (N = 15,442 non-missing) N	Percentage (95% CI)
Pakistani (N = 7,031)	609	9.8% (9.1%-10.6%)	1116	17.9% (16.9%-18.8%)	1141	18.0% (17.0%-18.9%)	2031	29.3% (28.2%-30.3%)
White British (N = 4,247)	432	11.1% (10.1%-12.1%)	470	11.9% (10.9%-12.9%)	880	22.3% (21.0%-23.6%)	912	21.6% (20.4%-22.9%)
Mixed (N = 900)	76	9.6% (7.8%-11.9%)	145	18.0% (15.5%-20.8%)	159	19.7% (17.1%-22.6%)	223	25.3% (22.5%-28.3%)
White Other (N = 707)	55	8.9% (6.9%-11.4%)	90	14.4% (11.8%-17.3%)	113	17.7% (14.9%-20.8%)	204	29.4% (26.1%-32.9%)
Bangladeshi (N = 471)	31	7.5% (5.3%-10.5%)	65	16.1% (12.8%-20.0%)	74	17.6% (14.2%-21.5%)	128	27.6% (23.7%-31.8%)
Indian (N = 357)	31	9.6% (6.8%-13.3%)	67	20.4% (16.4%-25.1%)	47	14.3% (10.9%-18.5%)	88	24.9% (20.7%-29.7%)
Black/ Black British (N = 277)	20	8.0% (5.2%-12.1%)	40	15.9% (11.9%-20.9%)	36	13.7% (10%-18.4%)	74	26.8% (21.9%-32.3%)
Gypsy/ Irish traveller (N = 190)	15	9.4% (5.7%-15%)	32	19.8% (14.3%-26.6%)	43	25.3% (19.3%-32.4%)	73	39.0% (32.3%-46.2%)
Other (N = 425)	33	8.9% (6.4%-12.2%)	56	14.9% (11.6%-18.8%)	81	21.3% (17.5%-25.7%)	101	24.4% (20.5%-28.8%)

Supplementary Table S4c – Friends and school

	Does not like school (N = 15,520 non-missing)		Not many friends (N = 15,527 non-missing)		Bullied some or all of the time (N = 15,368 non-missing)		Mean to others all the time (N = 15,245 non-missing)		Feel left out all the time (N = 15,456 non-missing)	
**Ethnicity**	**N**	**Percentage (95% CI)**	**N**	**Percentage (95% CI)**	**N**	**Percentage (95% CI)**	**N**	**Percentage (95% CI)**	**N**	**Percentage (95% CI)**
Pakistani (N = 7,031)	889	12.7% (12.0%-13.5%)	1029	14.7% (13.9%-15.6%)	3630	52.6% (51.4%-53.7%)	400	5.8% (5.3%-6.4%)	790	11.4% (10.6%-12.1%)
White British (N = 4,247)	652	15.4% (14.4%-16.6%)	662	15.7% (14.6%-16.8%)	2253	53.8% (52.3%-55.3%)	167	4.0% (3.5%-4.7%)	486	11.6% (10.6%-12.6%)
Mixed (N = 900)	128	14.4% (12.2%-16.8%)	113	12.7% (10.6%-15%)	459	51.9% (48.6%-55.1%)	41	4.7% (3.5%-6.3%)	98	11.0% (9.1%-13.2%)
White Other (N = 707)	69	9.8% (7.8%-12.3%)	106	15.2% (12.7%-18%)	328	47.1% (43.4%-50.8%)	44	6.4% (4.8%-8.5%)	74	10.6% (8.5%-13.1%)
Bangladeshi (N = 471)	54	11.5% (8.9%-14.8%)	53	11.3% (8.7%-14.5%)	235	51.1% (46.5%-55.6%)	24	5.2% (3.5%-7.6%)	40	8.5% (6.3%-11.4%)
Indian (N = 357)	33	9.3% (6.7%-12.8%)	46	13.0% (9.9%-16.9%)	183	52.0% (46.8%-57.2%)	15	4.3% (2.6%-7.0%)	31	8.8% (6.3%-12.3%)
Black/ Black British (N = 277)	32	11.6% (8.3%-15.9%)	23	8.3% (5.6%-12.2%)	144	52.9% (47%-58.8%)	14	5.2% (3.1%-8.5%)	24	8.8% (6.0%-12.8%)
Gypsy/ Irish traveller (N = 190)	20	10.7% (7.0%-16.0%)	30	16.0% (11.4%-21.9%)	79	42.2% (35.4%-49.4%)	18	9.9% (6.3%-15.2%)	21	11.3% (7.5%-16.7%)
Other (N = 425)	53	12.6% (9.8%-16.2%)	60	14.3% (11.3%-18%)	233	56.3% (51.5%-61.0%)	24	5.8% (3.9%-8.5%)	40	9.6% (7.1%-12.8%)

Supplementary Table S4d – Subjective wellbeing

	**Never happy** (N = 15,331 non-missing)		**Always sad** (N = 15,415 non-missing)		**Always ill or unwell** (N = 15,429 non-missing)		**Keeps worries to self** (N = 15,483 non-missing)		**Cannot work out what to do when things are hard** (N = 15,137 non-missing)	
**Ethnicity**	**N**	**Percentage (95% CI)**	**N**	**Percentage (95% CI)**	**N**	**Percentage (95% CI)**	**N**	**Percentage (95% CI)**	**N**	**Percentage (95% CI)**
Pakistani (N = 7,031)	281	4.1% (3.7%-4.6%)	406	5.9% (5.3%-6.4%)	622	9% (8.3%-9.7%)	2222	31.9% (30.8%-33%)	570	8.4% (7.7%-9.1%)
White British (N = 4,247)	168	4% (3.5%-4.7%)	223	5.3% (4.7%-6%)	305	7.2% (6.5%-8.1%)	1268	30.2% (28.8%-31.6%)	372	9% (8.2%-9.9%)
Mixed (N = 900)	38	4.3% (3.2%-5.9%)	55	6.2% (4.8%-8%)	73	8.3% (6.6%-10.3%)	281	31.5% (28.6%-34.7%)	88	10.1% (8.2%-12.2%)
White Other (N = 707)	26	3.8% (2.6%-5.5%)	43	6.2% (4.6%-8.2%)	50	7.2% (5.5%-9.4%)	210	30% (26.8%-33.5%)	69	10.1% (8.1%-12.7%)
Bangladeshi (N = 471)	14	3.1% (1.8%-5.1%)	21	4.5% (3%-6.9%)	45	9.7% (7.3%-12.8%)	139	29.8% (25.8%-34.1%)	22	4.8% (3.2%-7.1%)
Indian (N = 357)	6	1.7% (0.8%-3.8%)	20	5.7% (3.7%-8.7%)	22	6.3% (4.2%-9.4%)	112	31.6% (27%-36.7%)	23	6.7% (4.5%-9.9%)
Black/ Black British (N = 277)	6	2.2% (1%-4.8%)	16	5.8% (3.6%-9.3%)	19	6.9% (4.4%-10.5%)	79	28.6% (23.6%-34.2%)	15	5.6% (3.4%-9.1%)
Gypsy/ Irish traveller (N = 190)	6	3.3% (1.5%-7.1%)	13	7% (4.1%-11.7%)	16	8.6% (5.3%-13.5%)	60	32.3% (25.9%-39.3%)	27	15.2% (10.6%-21.2%)
Other (N = 425)	14	3.4% (2%-5.6%)	23	5.5% (3.7%-8.2%)	36	8.6% (6.2%-11.7%)	135	32.5% (28.1%-37.1%)	38	9.3% (6.8%-12.5%)

BMJ Publishing Group Limited (BMJ) disclaims all liability and responsibility arising from any reliance placed on this supplemental material which has been supplied by the author(s)

Supplementary Table S5: Associations of Index of Multiple Deprivation for each child wellbeing domain

IMD 2019 Quintile (1 = most materially deprived)	**Home, family and family relationships** (N = 5,703 non-missing)		**Material resources** (N = 5,153 non-missing)		**Friends and school** (N = 5,869 non-missing)		**Subjective wellbeing** (N = 5,706 non-missing)	
	**N**	**Percentage (95% CI)**	**N**	**Percentage (95% CI)**	**N**	**Percentage (95% CI)**	**N**	**Percentage (95% CI)**
1 (N = 4,334)	1817	45.4% (43.8%-46.9%)	1907	53.5% (51.9%-55.1%)	2713	65.7% (64.2%-67.1%)	1726	43.1% (41.6%-44.6%)
2 (N = 1,303)	511	42.0% (39.2%-44.8%)	546	48.5% (45.6%-51.5%)	797	64.0% (61.3%-66.6%)	482	39.5% (36.8%-42.3%)
3 (N = 308)	103	35.5% (30.0%-41.0%)	119	43.9% (38.0%-49.8%)	182	61.1% (55.5%-66.6%)	113	39.6% (34.0%-45.3%)
4 (N = 139)	50	38.5% (30.1%-46.8%)	49	37.1% (28.9%-45.4%)	80	60.6% (52.3%-68.9%)	47	35.1% (27.0%-43.2%)
5 (N = 59)	23	39.0% (26.5%-51.4%)	19	33.9% (21.5%-46.3%)	33	56.9% (44.2%-69.6%)	19	32.8% (20.7%-44.8%)
