# Supplementary material for: Vulnerabilities in child well-being among primary school children: a cross-sectional study in Bradford, UK
Source: BMJ Open. 2022 Jun 30;12(6):e049416. doi: 10.1136/bmjopen-2021-049416 (PMC9247690; doi:10.1136/bmjopen-2021-049416)
Supplement: Author's manuscript [file bmjopen-2021-049416.draft_revisions.pdf]

BMJ Open is committed to open peer review. As part of this commitment we make the peer review history of every article we publish publicly available.

When an article is published we post the peer reviewers' comments and the authors' responses online. We also post the versions of the paper that were used during peer review. These are the versions that the peer review comments apply to.

The versions of the paper that follow are the versions that were submitted during the peer review process. They are not the versions of record or the final published versions. They should not be cited or distributed as the published version of this manuscript.

BMJ Open is an open access journal and the full, final, typeset and author-corrected version of record of the manuscript is available on our site with no access controls, subscription charges or pay-per-view fees (http://bmjopen.bmj.com).

If you have any questions on BMJ Open's open peer review process please email info.bmjopen@bmj.com

BMJ Open

Vulnerabilities in child wellbeing among primary school children: a cross-sectional study in Bradford, UK

Journal:	*BMJ Open*
Manuscript ID	bmjopen-2021-049416
Article Type:	Original research
Date Submitted by the Author:	22-Mar-2021

Complete List of Authors:	Pickett, Kate; University of York<br>Ajebon, Mildred; University of York<br>Hou, Bo; Bradford Institute for Health Research<br>Kelly, Brian; Bradford Institute for Health Research<br>Bird, Philippa K; Leeds Teaching Hospitals NHS Trust<br>Dickerson, Josie; Bradford Institute for Health Research<br>Shire, Katy; Bradford Institute for Health Research<br>Mon-Williams, Mark; University of Leeds, Psychology<br>Small, Neil; University of Bradford<br>McEachan, Rosemary; Bradford Institute for Health Research<br>Wright, John; Bradford Institute for Health Research<br>Lawlor, Deborah; University of Bristol

Keywords:	COVID-19, Community child health < PAEDIATRICS, SOCIAL MEDICINE

SCHOLARONE™
Manuscripts

I, the Submitting Author has the right to grant and does grant on behalf of all authors of the Work (as defined in the below author licence), an exclusive licence and/or a non-exclusive licence for contributions from authors who are: i) UK Crown employees; ii) where BMJ has agreed a CC-BY licence shall apply, and/or iii) in accordance with the terms applicable for US Federal Government officers or employees acting as part of their official duties; on a worldwide, perpetual, irrevocable, royalty-free basis to BMJ Publishing Group Ltd ("BMJ") its licensees and where the relevant Journal is co-owned by BMJ to the co-owners of the Journal, to publish the Work in this journal and any other BMJ products and to exploit all rights, as set out in our licence.

The Submitting Author accepts and understands that any supply made under these terms is made by BMJ to the Submitting Author unless you are acting as an employee on behalf of your employer or a postgraduate student of an affiliated institution which is paying any applicable article publishing charge ("APC") for Open Access articles. Where the Submitting Author wishes to make the Work available on an Open Access basis (and intends to pay the relevant APC), the terms of reuse of such Open Access shall be governed by a Creative Commons licence – details of these licences and which Creative Commons licence will apply to this Work are set out in our licence referred to above.

Other than as permitted in any relevant BMJ Author's Self Archiving Policies, I confirm this Work has not been accepted for publication elsewhere, is not being considered for publication elsewhere and does not duplicate material already published. I confirm all authors consent to publication of this Work and authorise the granting of this licence.

For peer review only

Vulnerabilities in child wellbeing among primary school children: a cross-sectional study in Bradford, UK

Kate E Pickett[1*](0000-0002-8066-8507), Mildred Ajebon[1](0000-0001-5353-8080), Bo Hou[2](0000-0001-5337-6560), Brian Kelly[2](0000-0003-1834-2992), Philippa K Bird[3](0000-0002-9601-7979), Josie Dickerson[2](0000-0003-0121-3406), Katy Shire[2] (0000-0002-2093-181X) Mark Mon-Williams[4] (0000-0001-7595-8545), Neil Small[5] (0000-0002-4426-3596), Rosemary McEachan[2] (0000-0003-1302-6675), John Wright[2] (0000-0001-9572-7293) Deborah A Lawlor[6-8] (0000-0002-6793-2262)

***Corresponding author: kate.pickett@york.ac.uk**

Addresses:

[1]Department of Health Sciences, Seebohm Rowntree Building Area 2, University of York, Heslington, York, YO105DD

[2]Bradford Institute for Health Research, Bradford Teaching Hospitals NHS Foundation Trust, Bradford Royal Infirmary, Duckworth Lane, Bradford, BD9 6RJ, UK

[3]Leeds Teaching Hospitals NHS Foundation Trust, St. James's University Hospital, Beckett Street, Leeds, West Yorkshire, LS9 7TF

[4] School of Psychology, University of Leeds, Leeds LS2 9JT

[5] Faculty of Health Studies, University of Bradford, West Yorkshire BD7 1DP UK

[6]MRC Integrative Epidemiology Unit, University of Bristol, Oakfield House, Oakfield Grove, Bristol, BS8 2BN, UK

[7]Population Health Science, Bristol Medical School, University of Bristol, UK

[8]Bristol National Institute for Health Research Biomedical Research Centre, UK

ABSTRACT

Objective: To describe the prevalence of factors related to wellbeing among primary school children in a deprived multi-ethnic community.

Design and participants: Cross-sectional survey of 15,641 children aged 7-10 years in Born in Bradford's Primary School Years study: whole-classroom samples in 89 Bradford primary schools between 2016 and 2019.

Main outcome measures: Prevalence estimates by ethnicity (%, 95% CI) of single and multiple vulnerabilities in child wellbeing within and across four domains (home, family, relationships; material resources; friends and school; subjective wellbeing).

Results Only 10% of children have no vulnerabilities in any domain of wellbeing; 10% have one or more vulnerabilities in all four domains. The highest prevalence estimates were for being bullied some or all of the time (52.7%, 51.9 to 53.4%), keeping worries to oneself (31.2%, 30.5 to 31.9%), having no park near home (30.8%, 30.1 to 31.5%) and worrying all the time about how much money their family has (26.3%, 25.6 to 27%).

Boys were consistently significantly more likely than girls to report all of the vulnerabilities in the Home, Family and Family Relationships domain, and the majority of indicators in the other domains, and in all domains except Friends and School, boys were significantly more likely to have at least one vulnerability. Girls were significantly more likely to report not having many friends (16.7%, 95% CI: 15.9 to 17.6% vs. 12.5%, 95% CI: 11.8 to 13.2%), being bullied some or all of the time (55.8%, 95% CI: 54.7 to 56.9% vs. 49.7%, 95% CI: 48.6 to 50.8%) and feeling left out all the time (12.1%, 95% CI: 11.4 to 12.8%) vs. 10.3%, 95% CI: 9.7 to 11.0%).

Variations in vulnerabilities by ethnicity were complex, with children in Black, Asian and Minority Ethnic groups sometimes reporting more vulnerabilities and sometimes fewer than White British children. For example, compared to children of Pakistani heritage, White British children were more likely to say that their family never gets along well (6.3%,5.6 to 7.1% vs. 4.1%,3.6 to 4.6%) and to have no access to the internet at home (22.3%,21 to 23.6% vs. 18%,17 to 18.9%). Children with Pakistani heritage were more likely than White British

For peer review only - http://bmjopen.bmj.com/site/about/guidelines.xhtml

children to say they had no park near their home where they can play with friends (32.7%,31.6 to 33.9% vs. 29.9%,28.6 to 31.3%), to report not having three meals a day (17.9%,16.9 to 18.8% vs. 11.9%,10.9 to 12.9%) and to worry all the time about how much money their families have (29.3%,28.2 to 30.3%) vs. 21.6%,20.4 to 22.9%). Gypsy/Irish Traveller children were less likely than White British children to say they were bullied some or all of the time (42.2%,35.4 to 49.4% vs. 53.8%,52.3 to 55.3%), but more likely to say they were mean to others all the time (9.9%,6.3 to 15.2% vs. 4%,3.5 to 4.7%) and can never work out what to do when things are hard (15.2%,10.6 to 21.2% vs. 9%, 8.2 to 9.9%).

We considered six vulnerabilities to be of particular concern during the current Covid-19 pandemic and associated national and local lockdowns: family never gets along well together; no garden where child can play; no nearby park where they can play; not having 3 meals a day; no internet at home; worried about money all the time. Pre-pandemic, 37.4% (36.6 to 38.3%) of Bradford children had one of these vulnerabilities and a further 29.6% (28.9 to 30.4%) had more than one.

Conclusions

Although most primary school children aged 7-10 in our study have good levels of wellbeing on most indicators across multiple domains, fewer than 10% have no vulnerabilities at all, a worrying 10% have at least one vulnerability in all the four domains we studied and two thirds have vulnerabilities of concern during the Covid-19 lockdowns.

KEYWORDS

Children; subjective wellbeing; poverty; health inequalities; ethnicity; social determinants of health; cohorts; Born in Bradford; Covid-19

Summary

Strengths and limitations of this study

- A uniquely large study of contemporary child wellbeing in the UK with a high response rate and including a broad group of ethnicities, generalisable to other UK cities with high levels of ethnic diversity and deprivation
- Subjective measures of a wide range of experiences capture how children perceive their lives
- A descriptive study of prevalence of vulnerabilities in wellbeing, unable to examine causes

For peer review only

Introduction

In 2007, the UK ranked bottom of 21 countries in UNICEF's first report on child wellbeing in rich countries,[1] and from 2009 through 2019 the Good Childhood inquiry[2] and subsequent reports from the Children's Society[3] have found increasing levels of unhappiness and mental distress among children and young people in the UK. The State of the Nation 2019 report on children and young people's wellbeing suggested a need to "understand wellbeing across different groups" and to "use a range of measures to understand their experience."[4]

Wellbeing declines as children and young people grow older[4] but little is known about wellbeing in primary school aged children in the UK as previous studies, including the Good Childhood surveys, Understanding Society and HeadStart, include children over 10 years old only. The impact of ethnicity on child wellbeing at any age is unclear and most previous UK studies are unable to examine wellbeing across a wide range of ethnicities.[4] To provide evidence to meet these knowledge gaps, we examined child wellbeing among primary school children, aged 7 to 10 years, in a city in the North of England. Bradford is the fifth largest metropolitan district in England with one of the youngest and most ethnically diverse populations.[5] Bradford also has some of the highest levels of poverty and ill-health in England; almost a quarter of Bradford children live in poverty and 24% are obese at age 10/11.[6]

The global Covid-19 pandemic and restrictions, such as lockdowns that closed schools, heightened concerns about child wellbeing across the world. The prevalence of pre-existing child vulnerabilities and risk factors that could potentially require mitigation and targeted intervention during and after the pandemic has been a concern, particularly in poor, ethnically diverse urban settings.[7] The aim of this paper is to describe the prevalence of vulnerabilities in child wellbeing by ethnicity and gender in a deprived community. Whilst this research is undertaken in the city of Bradford and has been used to support local policy during the COVID-19 pandemic,[8] we believe the results have wider relevance for deprived multi-ethnic urban settings.

For peer review only - http://bmjopen.bmj.com/site/about/guidelines.xhtml

Methods (for STROBE statement see Supplementary Materials: 1)

Setting and Participants

Born in Bradford (BiB) is a cohort study of 13,500 children born between 2007-2011.[9] Between 2016 and 2019, BiB administered the Primary School Years study as a cross-sectional survey of wellbeing of children aged 7-10 years in 89 Bradford schools (Figure 1). We surveyed whole classrooms, including children previously recruited to the ongoing BiB cohort study as well as non-BiB children. The study protocol and detailed methods of school recruitment are described elsewhere.[10 11] Schools were given information sheets and opt-out consent forms to give to parents of children in eligible year groups ahead of the school visit; one school asked for opt-in consent to be used and this was accommodated. Fewer than 5% of parents opted out of the study.

Design and Procedures

Ethical approval was obtained from the NHS Health Research Authority's Yorkshire and the Humber - Bradford Leeds Research Ethics Committee (reference: 16/YH/0062) on the 24th March 2016.

The wellbeing survey ("Me and My Life") was developed for this study (see Supplementary Materials: 2), drawing on questions asked of children in the Millennium Cohort Study,[12] the International Survey of Children's Wellbeing[13] and the Avon Longitudinal Study of Parents and Children.[14] The survey aimed to assess children's wellbeing in multiple domains, including: happiness, health, material wellbeing, relationships with family and friends, school experience, neighbourhood, aspirations and acculturation. 'Think aloud' testing with a small group of children was used to check understanding and face validity. During administration, whole classrooms completed the survey at the same time, supported by three research facilitators.

Schools provided class lists, including date of birth, gender and ethnicity. Child ethnicity is reported by parents on registration with a school. The ethnicity information provided from school records contained 192 categories. These were re-coded into 18 broad groups in line with the 2011 census categorisation of ethnic groups in the UK and for the analyses presented here collapsed into 10 categories: Pakistani, Bangladeshi, Indian, Black/Black British, White British, Mixed, Gypsy/Irish Traveller, Other White, Other, and Unknown. Where ethnicity

For peer review only - http://bmjopen.bmj.com/site/about/guidelines.xhtml

was missing it was possible to supplement, for BiB children only, with data held by BiB (n=311).

To assess vulnerabilities in wellbeing, we grouped questions within four domains: (1) home, family and family relationships, (2) material resources, (3) friends and school, (4) subjective (self-reported) wellbeing. Within each domain, we defined 'vulnerabilities' on (a) the basis of research literature showing these to be well-established childhood risk factors for long-term health, wellbeing, educational attainment, and social mobility and (b) subsequent consultation with the vulnerabilities workstream of the Bradford Institute for Health Research Covid-19 Scientific Advisory Group (BIHR C-SAG) (https://www.bradfordresearch.nhs.uk/vulnerable-groups/). The risk factors categorised as vulnerabilities include, by domain:

• **Home, family, family relationships**: family never gets along well together, no garden where child can play, no nearby park where they can play, never plays in park

• **Material resources**: no warm winter coat, not having 3 meals a day, no internet at home, worried about money all the time

• **Friends and school**: doesn't like school, not many friends, bullied some or all of the time, mean to others all the time, feel left out all the time

• **Subjective wellbeing**: never happy, always sad, ill/unwell all of the time, keeps worries to self, can never work out what to do when things are hard

Data Analysis

Socio-demographics and the prevalence of vulnerabilities in child wellbeing are presented as counts and/or percentages with 95% confidence intervals, for all children and by gender and ethnicity. We also describe the number and percent of children with multiple vulnerabilities within and across domains of wellbeing by gender and ethnicity. Children with missing data for any answer are excluded from the relevant analyses and number of missing reported in figures and tables.

For peer review only - http://bmjopen.bmj.com/site/about/guidelines.xhtml

Results

Table 1: Socio-demographic characteristics of 15,641 Bradford primary school children

	**Number**	**Percent**
**Gender**		
Female	7647	48.9%
Male	7994	51.1%
**Age (years)**		
6	2	<0.01%
7	5264	33.7%
8	6901	44.1%
9	3175	20.3%
10	293	1.9%
11	1	<0.01%
12	3	<0.01%
13	2	<0.01%
Missing		
**Ethnicity**		
Pakistani	7031	45.0%
Bangladeshi	471	3.0%
Indian	357	2.3%
Black/Black British	277	1.8%
White British	4247	27.1%
Mixed	900	5.7%
Gypsy/Irish Traveller	190	1.2%
White Other	707	4.5%
Other	425	2.7%
Unknown	1036	6.6%

Table 1 shows the socio-demographics of the 15,641 survey respondents. Figure 2 presents the prevalence of each vulnerability across all domains for all children; Figure 3 presents the percentage of all children who have zero, one and more than one vulnerability within each domain.

For peer review only - http://bmjopen.bmj.com/site/about/guidelines.xhtml

Although the prevalence of vulnerability varies by item and domain, within each domain there is an item with notably high prevalence, with more than one in four children reporting vulnerability: no park near home (30.8%, 95% CI: 30.1-31.5%); worried about money all the time (26.3%, 95% CI: 25.6-27.0%); bullied some or all of the time (52.7%, 95% CI: 51.9-53.4%); and keeping worries to self (31.2%, 95% CI: 30.5-31.9%). We considered six vulnerabilities in child wellbeing would be of particular concern during lockdowns and school closures associated with the Covid-19 pandemic: family never gets along well together; no garden where child can play; no nearby park where they can play; not having 3 meals a day; no internet at home; worried about money all the time. Before the pandemic 37.4% (36.6-38.3%) of children had one of these vulnerabilities and a further 29.6% (28.9-30.4%) had more than one.

Although the majority of children have no vulnerabilities in the Home, Family, Relationships domain (55.9%, 95% CI: 55.0-56.7%) and the Subjective Wellbeing domain (57.4%, 95% CI: 56.6-58.2%), the majority of children have one or more vulnerabilities in the Material Resources, and Friends and School domains. Only 10% (add 95% CI) of children have no vulnerabilities in any domain of wellbeing. Figure 4 is a Venn diagram that shows how vulnerabilities overlap across domains. 1,494 (10%) children have no vulnerabilities. Each oval in the Venn diagram includes all the children who have one or more vulnerabilities within that domain; so, for example, 2% of children have vulnerabilities in Home, Family, Relationships *and* Subjective Wellbeing but not in other domains. 1,519 children (10%) have one or more vulnerabilities in all four domains.

Variation by gender

Boys were consistently significantly more likely to report all of the vulnerabilities in the Home, Family and Family Relationships domain, although the gender differences were not large (see Supplementary Materials: Table 3 for full results, including 95% confidence intervals, for all domains by gender). There were no gender differences in having access to the internet at home, but boys were significantly more likely to report not having a winter coat (11.1%, 95% CI: 10.4-11.9% vs 8.9%, 95% CI: 8.2-9.6%), not having three meals a day (17.7%, 95% CI: 16.8-18.6%) vs 14.0%, 95% CI: 13.3-14.9%) and worrying about money all the time (28.9%, 95% CI: 28.0-30.0%) vs. 23.4%, 95% CI: 22.5-24.4%). Within the Friends and School domain, boys were significantly more likely to not like school (18.0%, 95% CI: 17.1-18.8%) vs. 8%, 95% CI: 7.4-8.6%) and to be mean to others all the time (7.0%, 95% CI:

For peer review only - http://bmjopen.bmj.com/site/about/guidelines.xhtml

6.4-7.6% vs. 3.4%, 95% CI: 3.0-3.8%), while girls were significantly more likely to report not having many friends (16.7%, 95% CI: 15.9-17.6% vs. 12.5%, 95% CI: 11.8-13.2%), being bullied some or all of the time (55.8%, 95% CI: 54.7-56.9% vs. 49.7%, 95% CI: 48.6-50.8%) and feeling left out all the time (12.1%, 95% CI: 11.4-12.8%) vs. 10.3%, 95% CI: 9.7-11.0%). Within the Subjective Wellbeing domain there were no gender differences in being always sad or always ill or unwell, but boys were significantly likely to report never being happy (5.2%, 95% CI: 4.7-5.7%) vs. 2.4%, 95% CI: 2.1-2.8%), keeping worries to themselves (33.7%, 95% CI: 32.7-34.8% vs. 28.5%, 95% CI: 27.5-29.6%) and not being about to work out what to do when things are hard (9.6%, 95% CI: 9.0-10.3% vs. 7.5%, 95% CI: 6.9-8.1%). In all domains except for Friends and School, boys were significantly more likely to have at least one vulnerability, compared to girls.

Variation by ethnicity

Figures 5a-5d shows the percentages (and 95% confidence intervals) of primary school children with at least one vulnerability within each of the domains of child wellbeing, by ethnicity. Full results are given in Supplementary Materials, Tables S4a-4d.

Home, family, relationships (Figure 5a)

Compared to White British children, Pakistani, White Other and Black/Black British children were significantly less likely to say their family never get along well together. White Other and Other children were significantly more likely to not have a garden where they can play, compared to White British children. Compared to White British children, Pakistani children were significantly more likely, and Black/Black British children significantly less likely to have no park near home where they can play with friends. Compared to White British children, Pakistani children were significantly less likely to say they never play in a park.

Material resources (Figure 5b)

There were no significant ethnic differences in the proportion of children who did not have a warm winter coat. Compared to White British children, Pakistani, Indian and Gypsy/Traveller children, as well as children of Mixed ethnicity were significantly more likely to report not having three meals a day. Pakistani, White Other, Indian and Black/Black British children were significantly less likely to report having no internet at home than White British children. Compared to White British children, Pakistani, White Other, Bangladeshi and Gypsy/Irish Traveller children were significantly more likely to worry all the time about how much money their families have.

For peer review only - http://bmjopen.bmj.com/site/about/guidelines.xhtml

<u>Friends and school Figure 5c</u>

Pakistani, White Other and Indian children were significantly less likely to report not liking school than White British children, and Bangladeshi and Black/British were significantly less likely to report not having many friends. Compared to White British children, White Other and Gypsy/Irish Traveller children were significantly less likely to say they were bullied some or all of the time, and Pakistani children and Gypsy/Irish traveller children were significantly more likely to say they were mean to others all the time. There were no significant ethnic differences in the proportion of children who felt left out by others all the time.

<u>Subjective wellbeing Figure 5d</u>

There were no significant ethnic differences in the proportion of children who said they were never happy or were always sad or who said they keep worries to themselves. Compared to White British children, only Pakistani children were significantly more likely to say they were always ill or unwell. Compared to White British children, Bangladeshi children were significantly less likely, and Gypsy/Irish traveller children more likely, to say they cannot work out what to do when things are hard.

For peer review only - http://bmjopen.bmj.com/site/about/guidelines.xhtml

Discussion

Statement of Principal Findings

Although most primary school children aged 7-10 in Bradford have good levels of wellbeing on most indicators across multiple domains, fewer than 10% have no vulnerabilities at all, and a worrying 10% have more than one vulnerability in all the four domains we studied. Variations in vulnerabilities by ethnicity were complex, with children in Black, Asian and Minority Ethnic groups sometimes reporting more vulnerabilities and sometimes fewer than White British children. We considered six vulnerabilities to be of particular concern during the current Covid-19 pandemic and associated national and local lockdowns (family never gets along well together; no garden where child can play; no nearby park where they can play; not having 3 meals a day; no internet at home; worried about money all the time). Before the pandemic, the majority – two-thirds – of Bradford children had one or more of these vulnerabilities. The domain with the highest proportion of children reporting vulnerabilities was Friends and School with 1 in 20 children reporting that they are mean to other children all the time, more than 1 in 10 reporting feeling left out all the time, not having many friends and not liking school, and the majority of children reporting being bullied some or all of the time.

We found that boys reported significantly more vulnerabilities for all indicators in the Home, Family and Family Relationships domain, as well as for the majority of indicators in the Material Resources, Friends and School, and Subjective Wellbeing domains. This contrasts with the finding of few consistent gender differences in wellbeing and a trend towards worse wellbeing in girls in the UK State of the Nation 2019: Children and Young People's Wellbeing report[4], and the general picture in the research literature of worse wellbeing for adolescent girls[15 16] and inconsistent gender differences among adults.[17 18] These differences may be due to the younger age of our sample, and suggest that worse wellbeing among adolescent girls might not be inevitable if prevention efforts were started before secondary education.

We found complex variations in vulnerabilities by ethnicity, with children in Black, Asian and Minority Ethnic groups sometimes reporting more vulnerabilities and sometimes fewer than White British children. For example, compared to children of Pakistani heritage, White British children were more likely to say that their family never gets along well and to have no access to the internet at home, whereas children with Pakistani heritage were more likely

For peer review only - http://bmjopen.bmj.com/site/about/guidelines.xhtml

than White British children to say they had no park near their home where they can play with friends, to report not having three meals a day and to worry all the time about how much money their families have. The UK State of the Nation 2019: Children and Young People's Wellbeing report drew attention to the importance of "*moving beyond the average*" in understanding wellbeing but reported "no discernible differences in wellbeing by…ethnicity" whilst recognising that small sample sizes might have obscured differences and reporting statistics only for 5 broadly defined ethnic groups. We provide a detailed description of wellbeing for 9 ethnic groups, so that nuances in ethnic variation can be better understood, including for smaller ethnic groups. For example, we found that Gypsy/Irish Traveller children were less likely than White British children to say they were bullied some or all of the time, but more likely to say they were mean to others, can never work out what to do when things are hard, worry all the time about how much money their family has, and not have 3 meals a day.

Strengths and weaknesses of the study

To our knowledge, ours is the only large contemporary study of wellbeing in primary school aged children in England, with sufficient numbers of children belonging to some of the UK's smaller Black, Asian and Minority Ethnic communities to be able to describe their wellbeing. Our approach to school recruitment, opt-out consent and whole classroom testing, along with Born in Bradford's longstanding trusting relationships with schools and communities ensured a very high response rate. Although our study is based in a single city, it is likely to be generalizable to other UK cities with high levels of ethnic diversity and deprivation.

The fact that our measures of wellbeing are self-reported by children is both a strength and a potential weakness. It has been argued that, for adults and children alike, the subjective experience of quality of life matters, and that, as feelings have a demonstrable objective neuropsychological reality and are correlated with objective health, social and economic outcomes, they are worthy of measurement and inference. [19 20] Clearly, our measures of vulnerability not only include things that can only be measured subjectively, such as happiness or feeling left out, but also things that arguably would be better measured objectively, e.g., having access to a garden or three meals a day. However, it is possible that even for measures such as these, children's perceptions that they lack material resources may be important even if they actually have the resources.

For peer review only - http://bmjopen.bmj.com/site/about/guidelines.xhtml

Our data collection objectives and approach to categorising vulnerabilities in wellbeing is very different to the Adverse Childhood Experiences (ACEs) approach[21] and the definition and measurement of extreme child vulnerabilities collated and reported by the Children's Commissioner for England (https://www.childrenscommissioner.gov.uk/chldrn/). ACEs are traumatic experiences that include abuse and neglect, parental mental illness, substance use and divorce. The child vulnerabilities reported by the Children's Commissioner include living in a household where an adult has a drug or alcohol dependence, where an adult has experienced violence or abuse from a partner, where an adult has a clinically diagnosable mental health condition or more than one of these factors. Both ACEs and the Children's Commissioner approaches identify factors that put children at immediate, as well as long-term, risk of harm. Our approach was to collect data on more common risk factors with known long-term consequences for health and wellbeing, to better understand children's subjective experiences across the whole population.

In our school-based whole classroom surveys, time constraints and study design prevented data collection and analyses that would have allowed us to go beyond description and investigate the causes of vulnerabilities in child wellbeing. For BiB children only, we examined associations between neighbourhood area deprivation (Index of Multiple Deprivation) and vulnerabilities. We found no significant associations for any domain except for more vulnerabilities in Material Resources at higher levels of deprivation, and as lack of material resources is definitional for deprivation do not report those analyses here. We plan future data collection with this sample to allow us to examine how vulnerabilities at primary school ages shape trajectories of wellbeing across time.

Implications for providers of services to children and policymakers

Our findings suggest that, prior to the Covid-19 pandemic, a substantial number of children were experiencing sub-optimal wellbeing with potential lifelong consequences, with implications for all providers of services to children, as well as local and national policymakers. As the economic impact of the pandemic is increasing child poverty, income insecurity, food and housing insecurity, parental mental health challenges and family violence, the need for attention to the identification and amelioration of vulnerability is heightened.[22] The International Society for Social Pediatrics and Child Health has called for the adoption of "a Child Rights Based Approach (CRBA) to respond to the COVID-19 pandemic, and to advance a future in which the health, development and well-being of

For peer review only - http://bmjopen.bmj.com/site/about/guidelines.xhtml

children and youth are prioritised with explicit strategies to reduce health inequities and advance social justice" (https://www.issop.org/2020/06/01/issop-covid-19-declaration/). Such an approach can underpin and inform services and policy making at all levels.

As the population was encouraged to 'lockdown' at home and schools closed for almost all children (and attendance was very low for those who were eligible to go to school) for varying periods, the response to the pandemic will have affected children's wellbeing (as well as their educational trajectories) differently, depending on their circumstances and context. Children who found school a nurturing refuge from difficult home situations will have suffered from school shutdowns, whereas others who found school a challenging environment may have been happy to be at home for a prolonged period; most children have probably experienced both positive and negative impacts. Although all schools work hard to create safe and supportive environments for children, the high levels of bullying and social isolation that we report suggests that more needs to be done to make this a reality for all children. In addition, the high prevalence of children with no access to the internet at home and no access to a garden or park mean that many children will have at times been unable to study or be physically active.

Locally, our research team has engaged with our local authority, public health, NHS and voluntary sector colleagues to identify principles to mitigate the impact of lockdown exit on vulnerable groups (https://www.bradfordresearch.nhs.uk/wp-content/uploads/2020/04/CSAG-Briefing-Paper-Vulnerable-Groups-and-Recovery-230420.pdf). We have proposed additional support for children and young people in the lockdown exit and recovery planning, including assessing the impact of decision making on children, prioritising children's needs, providing extra support for vulnerable children, and monitoring impacts on children's lives and wellbeing.

Conclusions and implications for public health

Among primary school children aged 7-10 in a deprived Northern city in the UK, fewer than 10% have no vulnerabilities in home, family and family relationships, material resources, friends and school and subjective wellbeing, while 10% have more than one vulnerability in all of these domains. These children are at risk of poor health, social and economic trajectories throughout their lives that could be mitigated by policy focused at structural

For peer review only - http://bmjopen.bmj.com/site/about/guidelines.xhtml

factors, such as poverty eradication, and child services targeted at reducing inequalities and supporting the most vulnerable.

For peer review only

References

1. UNICEF Innocenti Research Centre. Child poverty in perspective: An overview of child well-being in rich countries. Florence: Innocenti Report Card, 2007.

2. Layard R, Dunn J. A Good Childhood: Searching for values in a competitive age. London: Penguin 2009.

3. The Children's Society. The Good Childhood Report. London: The Children's Society, 2012-2019.

4. Department for Education. State of the Nation 2019: Children and Young People's Wellbeing. London: Department for Education, UK Government, 2019.

5. Office for National Statistics. 2011 Census: Key Statistics and Quick Statistics for Local Authorities in the United Kingdom. 2013, 2013.

6. Public Health England. Public Health Outcomes Framework: Yorkshire and Humber 2020 [Available from: https://tinyurl.com/y2y8yo84 accessed 25 July 2020.

7. Health ISfSPaC. Declaration: Advancing Health Equity and Social Justice in Response to COVID-19: https://www.issop.org/cmdownloads/issop_covid-declaration/, 2020.

8. Bradford Institute for Health Research. Overview: BIHR Covid-19 Scientific Advisory Group 2020 [Available from: https://www.bradfordresearch.nhs.uk/wp-content/uploads/2020/05/C-SAG-Overview-210520.pdf accessed 25 July 2020.

9. Raynor P, Born in Bradford Collaborative G. Born in Bradford, a cohort study of babies born in Bradford, and their parents: Protocol for the recruitment phase. *BMC public health* 2008;8(1):327.

10. Bird PK, McEachan RRC, Mon-Williams M, et al. Growing up in Bradford: protocol for the age 7-11 follow up of the Born in Bradford birth cohort. *BMC public health* 2019;19(1):939. doi: 10.1186/s12889-019-7222-2 [published Online First: 2019/07/14]

11. Shire KA, Mon-Williams M, Hill LJB, et al. The Born in Bradford Primary School Years project: In school assessment of 7-11 year old's cognitive and sensorimotor abilities. *Wellcome Open Research* In preparation

12. National Centre for Social Research. Child of the new century: age 7 survey: http://doc.ukdataservice.ac.uk/doc/6411/mrdoc/pdf/mcs4_child_self_completion.pdf, 2008.

13. Wellbeing ISoCs. Children's Worlds: ISCWeB: https://isciweb.org/wp-content/uploads/2019/12/Childrens-Worlds_Q08_100713_Website.pdf, 2017.

For peer review only - http://bmjopen.bmj.com/site/about/guidelines.xhtml

14. Avon Longitudinal Study of Parents and Children. Child completed questionnaires: http://www.bristol.ac.uk/alspac/researchers/our-data/questionnaires/child-completed-questionnaires/, Accessed: 30/12/2020.

15. Public Health England. Wellbeing of adolescent girls: An analysis of data from the Health Behaviour in School-aged Children (HBSC) survey for England, 2014 London: Public Health England, 2017.

16. Deighton J, Lereya T, Patalay P, et al. Mental health problems in young people aged 11-14: Results from the first HeadStart annual survey of 30,000 children. London: CAMHS Press 2018.

17. Batz C, & Tay, L. . Gender differences in subjective well-being. In: E. Diener SO, & L. Tay, ed. Handbook of well-being. Salt Lake City, UT: DEF Publishers 2018.

18. Batz-Barbarich C, Tay L, Kuykendall L, et al. A Meta-Analysis of Gender Differences in Subjective Well-Being: Estimating Effect Sizes and Associations With Gender Inequality. *Psychological science* 2018;29(9):1491-503. doi: 10.1177/0956797618774796

19. Bradshaw J. The well-being of children in the UK. Bristol: Policy Press 2016.

20. Ben-Arieh A. Developing indicators for child well-being in a changing context. In: McAuley R, Rose W, eds. Child well-being: Understanding children's lives. London: Jessica Kingley Publishers 2010:129-42.

21. Felitti VJ, Anda RF, Nordenberg D, et al. Relationship of childhood abuse and household dysfunction to many of the leading causes of death in adults. The Adverse Childhood Experiences (ACE) Study. *Am J Prev Med* 1998;14(4):245-58. doi: 10.1016/s0749-3797(98)00017-8 [published Online First: 1998/06/23]

22. Dickerson J KB, Lockyer B, Bridges S, Cartwright C, Willan K, Shire, K, Crossley K, Sheldon TA, Lawlor DA, Wright J, McEachan RRC, Pickett KE. Experiences of lockdown during the Covid-19 pandemic: descriptive findings from a survey of families in the Born in Bradford study [version 1; peer review: 1 approved, 1 approved with reservations]. . *Wellcome Open Research* 2020;5(228):(https://doi.org/10.12688/wellcomeopenres.6317.1).

For peer review only - http://bmjopen.bmj.com/site/about/guidelines.xhtml

Ethics approval

Ethical approval has been obtained from the National Health Service Health Research Authority Yorkshire and the Humber (Bradford Leeds) Research Ethics Committee (reference: 16/YH/0062).

Transparency statement

Kate Pickett (the manuscript's guarantor) affirms that the manuscript is an honest, accurate, and transparent account of the study being reported; that no important aspects of the study have been omitted; and that any discrepancies from the study as originally planned have been explained.

Competing interests

No authors declare competing interests.

Contributions

KEP, DAL, JWr RRCM, PKB, MM, NS conceived the study and obtained funding. BK, BH, MA, KS curated the data, conducted analyses and contributed to the methodology. KEP drafted the first manuscript. All authors read and approved the final version of the manuscript.

Acknowledgements

This study was funded by a joint grant from the UK Medical Research Council and UK Economic and Social Science Research Council (MR/N024397/1), with support from a Wellcome Trust infrastructure grant (WT101597MA) and the National Institute for Health Research under its Applied Research Collaboration Yorkshire and Humber (NIHR200166). The funders had no role in the design of the study, the collection, analysis, or interpretation of the data; the writing of the manuscript, or the decision to submit the manuscript for publication. All authors are independent of the funders, had full access to all of the data (including statistical reports and tables) in the study and can take responsibility for the integrity of the data and the accuracy of the data analysis. Views expressed in this paper are those of the authors and not necessarily those of any funder.

Born in Bradford is only possible because of the enthusiasm and commitment of the Children and Parents in BiB. We are grateful to all the participants, health professionals, schools, and researchers who have made Born in Bradford happen. Research results are

For peer review only

disseminated through https://borninbradford.nhs.uk/our-findings/ and actively through various engagement activities to parents, children and young people and stakeholders.

Patient and Participant Involvement

BiB is committed to the co-production of research. Our child wellbeing survey was shared with the Parent Governors group for feedback during development, and piloted with Bradford school children.

Summary

What is already known on this topic

- Child wellbeing in the UK is low in comparison to other rich countries
- Little is known about the wellbeing of children under the age of 10 in the UK
- Little is known about ethnic variations in child wellbeing in the UK

What this study adds

- Although most 7-10 year old children in our study have good levels of wellbeing, fewer than 10% have no vulnerabilities at all, a worrying 10% have at least one vulnerability in all the domains we studied, and two thirds have vulnerabilities of concern during the Covid-19 lockdowns
- In contrast to studies of older children, boys had more wellbeing vulnerabilities, although girls were more likely to have no friends, report being bullied and feeling left out.
- Ethnic variations in vulnerabilities in wellbeing were complex, with children from ethnic minorities reporting fewer of some vulnerabilities than White British children and more of others; our study is the first to report a wide range of vulnerabilities across a large number of ethnic groups in this age range

For peer review only - http://bmjopen.bmj.com/site/about/guidelines.xhtml

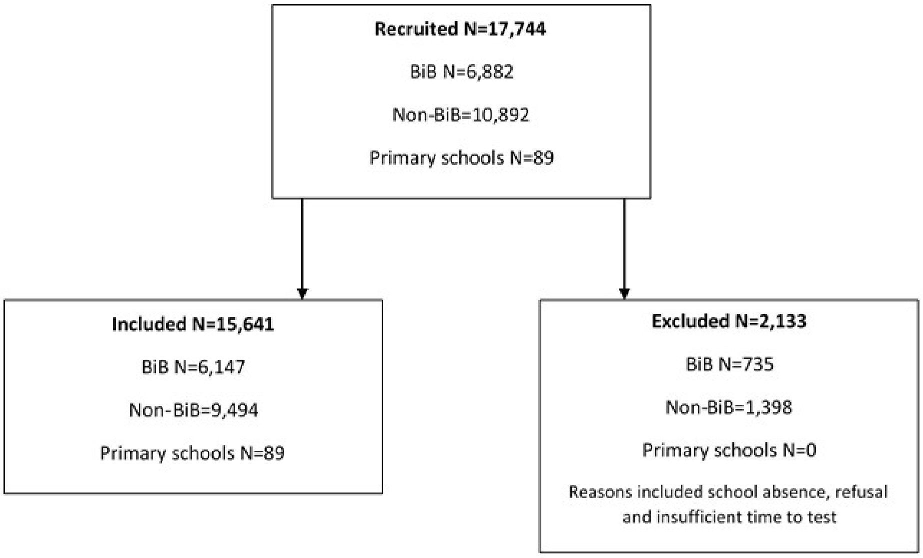

Figure 1: Recruitment of schools, Born in Bradford cohort members and non-Born in Bradford children to the BiB Primary School Years wellbeing survey

184x149mm (96 x 96 DPI)

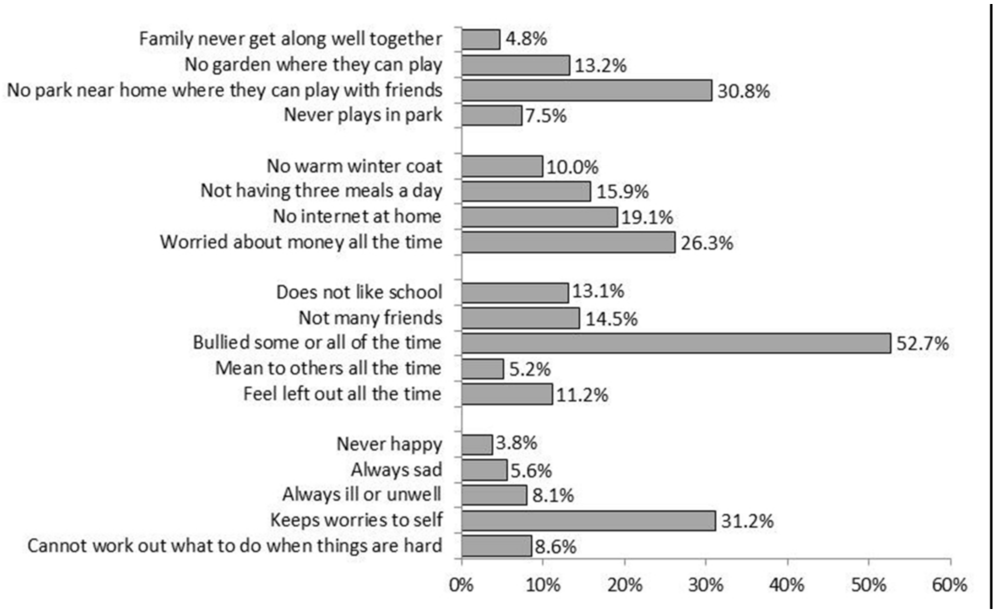

Figure 2: Percent of primary school children with each vulnerability across domains of wellbeing*
* For 15,641 children: each item has missing data of 3% or less, with the exception of items "No winter coat", "Not having three meals a day" and "No internet at home" which have around 10% missing data. For more details, and confidence intervals, see Supplementary Materials: Table S1.

1079x653mm (96 x 96 DPI)

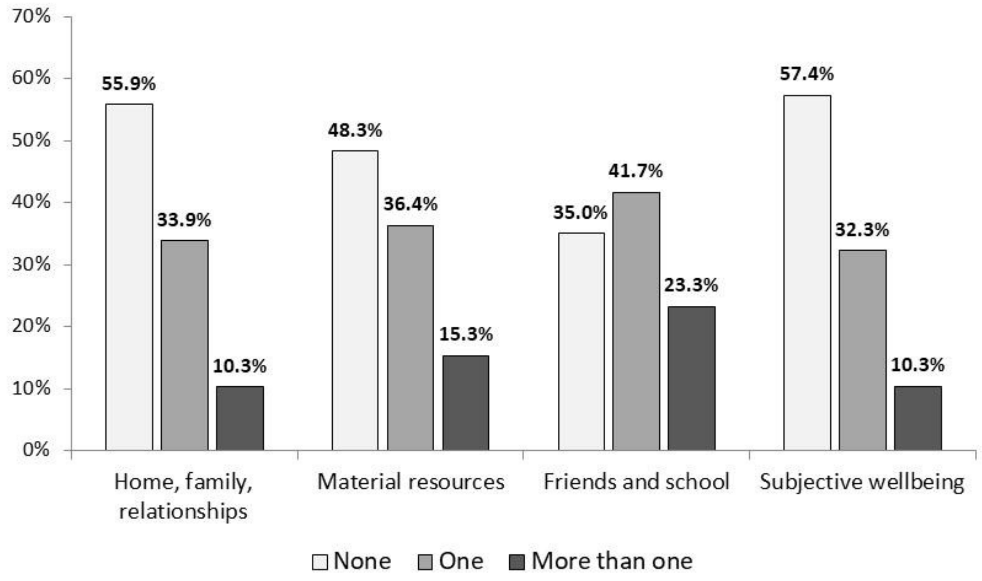

Figure 3: Percentage of primary school children with zero, one, or more than one vulnerability in each domain of wellbeing*

*For 15,641 children: the percentage with missing data for at least one item in each domain is between 5% and 16%. For more details, and confidence intervals, see Supplementary Materials: Table S2.

172x103mm (120 x 120 DPI)

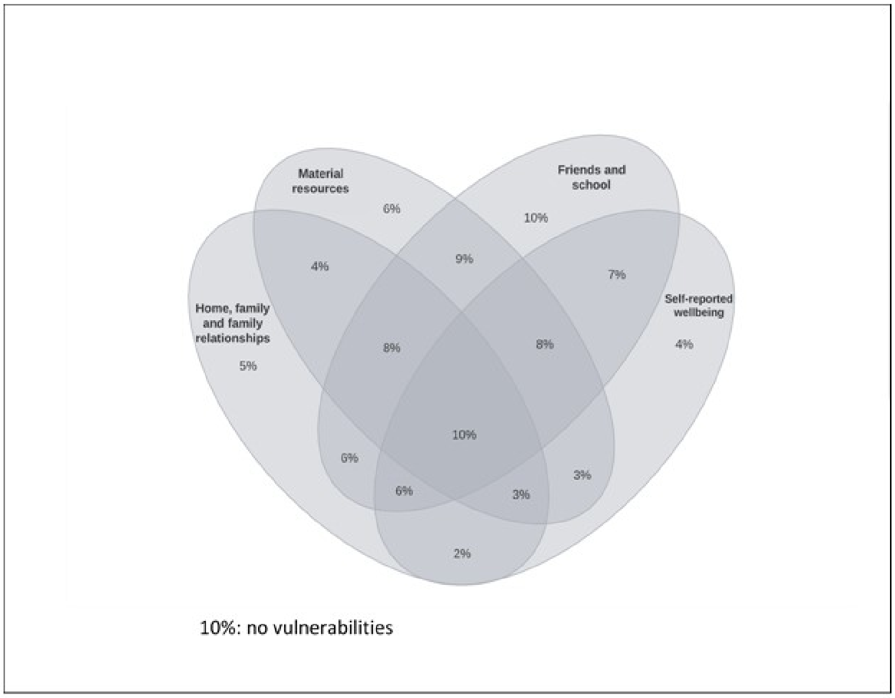

Figure 4: Percentage of children with vulnerabilities within and across domains (N=15,641)

184x149mm (96 x 96 DPI)

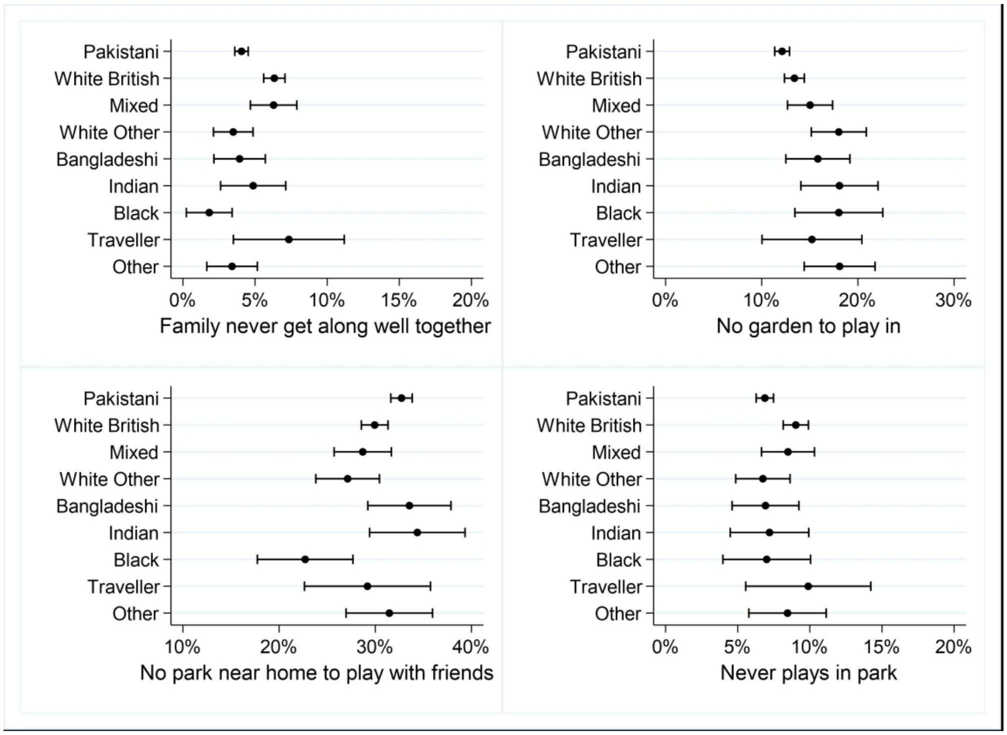

Figure 5a: Percentage of primary school children with at least one vulnerability in the Home, Family and Family Relationships domain

875x636mm (96 x 96 DPI)

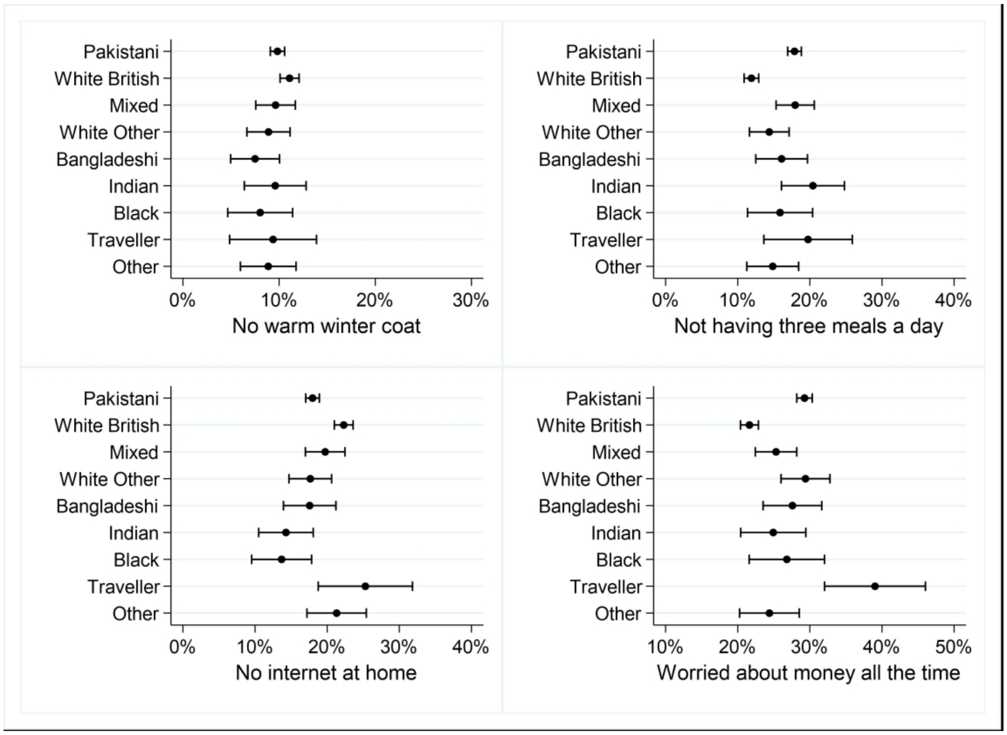

Figure 5b: Percentage of primary school children with at least one vulnerability in the Material Resources domain

875x636mm (96 x 96 DPI)

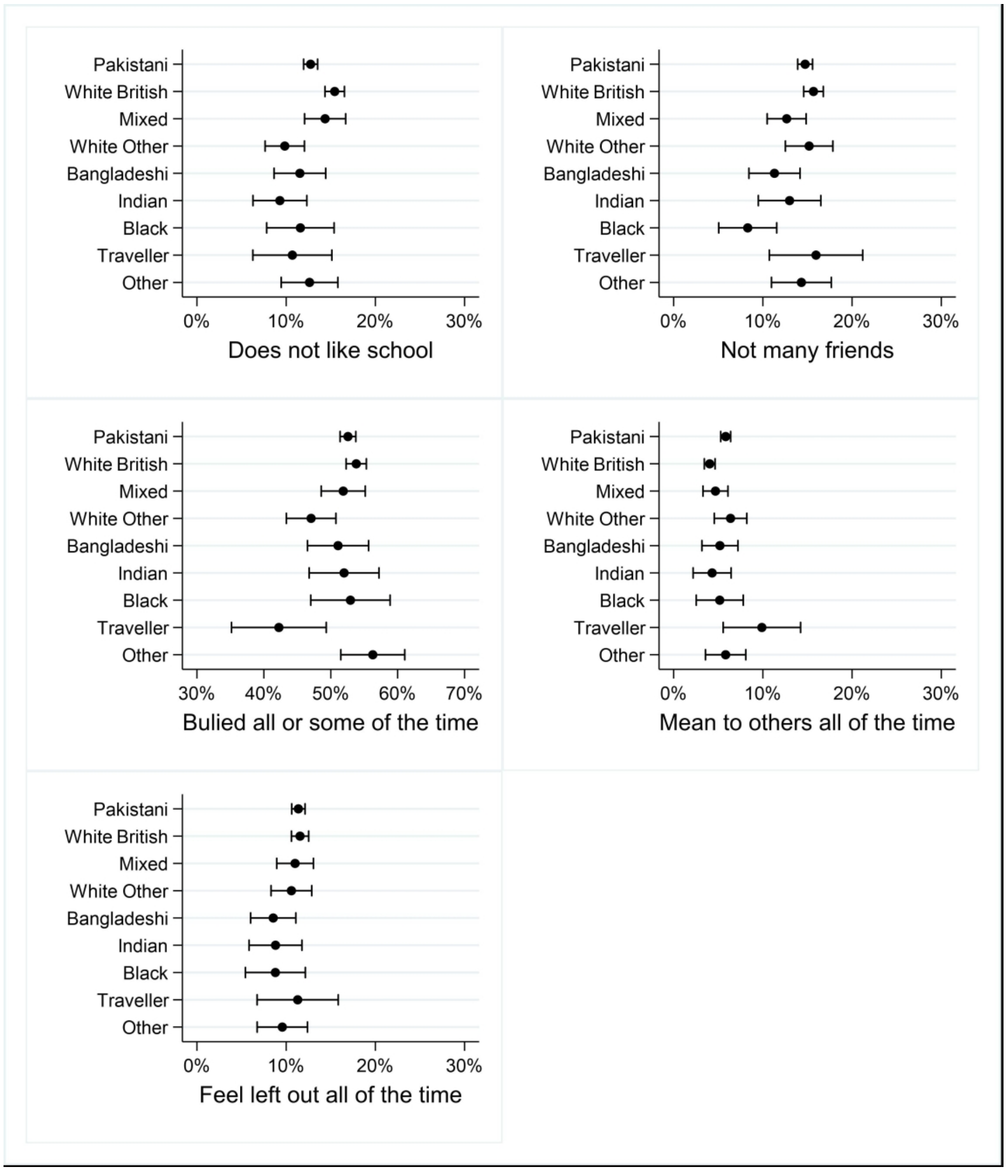

Figure 5c: Percentage of primary school children with at least one vulnerability in the Friends and School domain

875x1017mm (96 x 96 DPI)

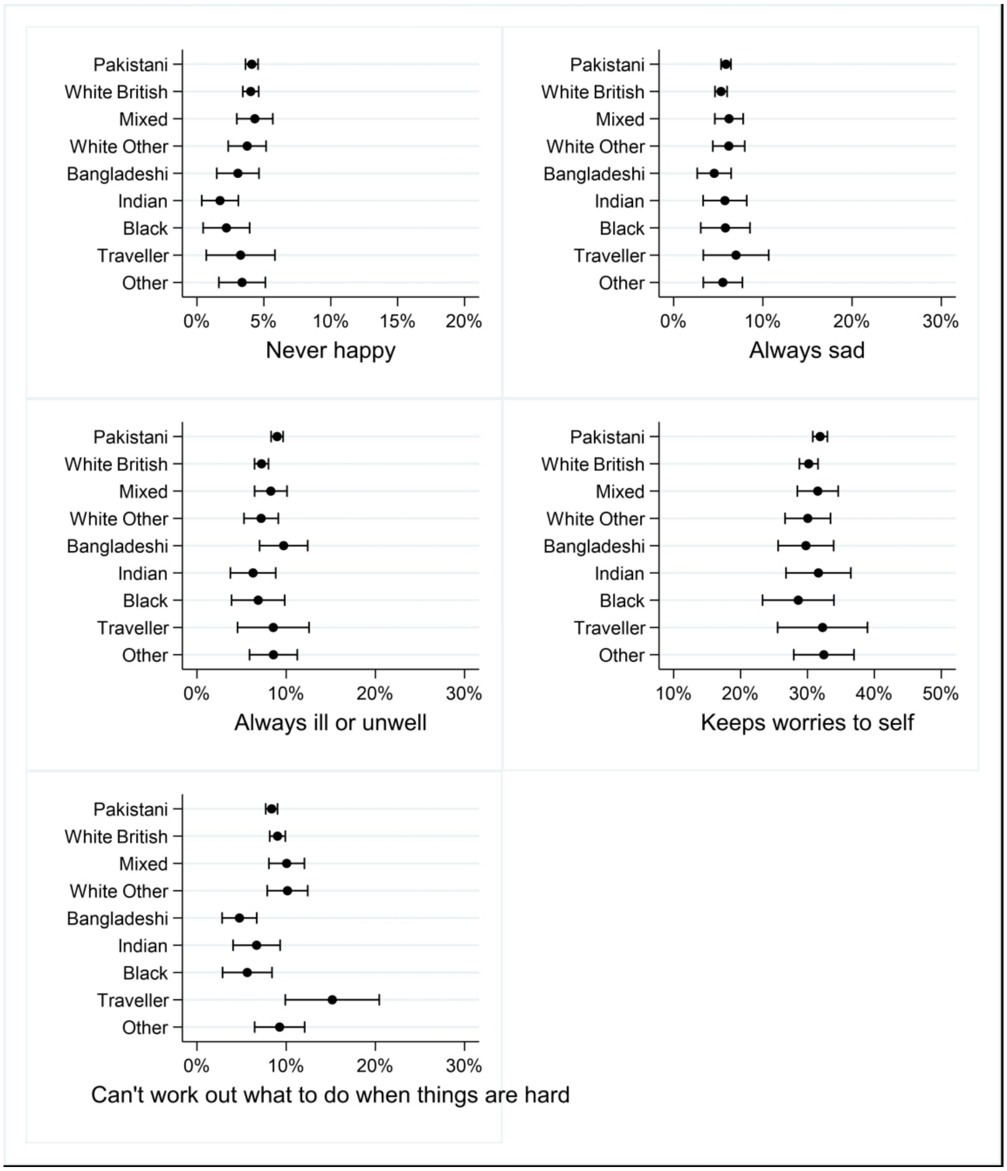

Figure 5d: Percentage of primary school children with at least one vulnerability in the Subjective Wellbeing domain

875x1017mm (96 x 96 DPI)

Supplementary Materials: Vulnerabilities in child wellbeing among primary school children: a cross sectional study in Bradford, UK

1: STROBE Statement—Checklist of items that should be included in reports of ***cross-sectional studies***

	Item No	Recommendation
**Title and abstract**	1	(*a*) Indicate the study's design with a commonly used term in the title or the abstract SEE TITLE
		(*b*) Provide in the abstract an informative and balanced summary of what was done and what was found SEE ABSTRACT
**Introduction**		
Background/rationale	2	Explain the scientific background and rationale for the investigation being reported<br>SEE INTRODUCTION, PAGE 4
Objectives	3	State specific objectives, including any prespecified hypotheses<br>N/A
**Methods**		
Study design	4	Present key elements of study design early in the paper<br>SEE METHODS, PAGES -7
Setting	5	Describe the setting, locations, and relevant dates, including periods of recruitment, exposure, follow-up, and data collection<br>SEE SETTINGS AND PARTICIPANTS, PAGE 5
Participants	6	(*a*) Give the eligibility criteria, and the sources and methods of selection of participants<br>SEE SETTINGS AND PARTICIPANTS, PAGE 5
Variables	7	Clearly define all outcomes, exposures, predictors, potential confounders, and effect modifiers. Give diagnostic criteria, if applicable<br>SEE DESIGN AND PROCEDURES, PAGE 6
Data sources/ measurement	8*	For each variable of interest, give sources of data and details of methods of assessment (measurement). Describe comparability of assessment methods if there is more than one group<br>SEE DESIGN AND PROCEDURES, PAGE 6
Bias	9	Describe any efforts to address potential sources of bias<br>SEE FIGURE 1, PAGE 5
Study size	10	Explain how the study size was arrived at<br>SEE SETTINGS AND PARTICIPANTS, PAGE 5
Quantitative variables	11	Explain how quantitative variables were handled in the analyses. If applicable, describe which groupings were chosen and why<br>SEE DESIGN AND PROCEDURES, PAGE 6
Statistical methods	12	(*a*) Describe all statistical methods, including those used to

For peer review only

		control for confounding
		(*b*) Describe any methods used to examine subgroups and interactions
		(*c*) Explain how missing data were addressed
		(*d*) If applicable, describe analytical methods taking account of sampling strategy
		(*e*) Describe any sensitivity analyses<br>SEE DATA ANALYSIS, PAGE 7
**Results**		
Participants	13*	(a) Report numbers of individuals at each stage of study—eg numbers potentially eligible, examined for eligibility, confirmed eligible, included in the study, completing follow-up, and analysed
		(b) Give reasons for non-participation at each stage
		(c) Consider use of a flow diagram<br>SEE METHODS, FIGURE 1, PAGE 5 AND ALL RESULTS TABLES
Descriptive data	14*	(a) Give characteristics of study participants (eg demographic, clinical, social) and information on exposures and potential confounders<br>SEE TABLE 1, PAGE 8
		(b) Indicate number of participants with missing data for each variable of interest<br>SEE ALL TABLES AND FIGURES
Outcome data	15*	Report numbers of outcome events or summary measures<br>SEE FIGURE 1, PAGE 9
Main results	16	(*a*) Give unadjusted estimates and, if applicable, confounder-adjusted estimates and their precision (eg, 95% confidence interval). Make clear which confounders were adjusted for and why they were included<br>SEE ALL TABLES AND FIGURES
		(*b*) Report category boundaries when continuous variables were categorized<br>N/A
		(*c*) If relevant, consider translating estimates of relative risk into absolute risk for a meaningful time period<br>N/A
Other analyses	17	Report other analyses done—eg analyses of subgroups and interactions, and sensitivity analyses<br>SEE DISCUSSION RE: UNREPORTED ANALYSIS OF IMD, PAGE 18
**Discussion**		
Key results	18	Summarise key results with reference to study objectives<br>SEE STATEMENT OF PRINCIPAL FINDINGS, PAGE 16
Limitations	19	Discuss limitations of the study, taking into account sources of potential bias or imprecision. Discuss both direction and magnitude of any potential bias

For peer review only

		SEE STRENGTHS AND WEAKNESSES OF THE STUDY, PAGE 17
Interpretation	20	Give a cautious overall interpretation of results considering objectives, limitations, multiplicity of analyses, results from similar studies, and other relevant evidence<br>SEE IMPLICATIONS SECTION, PAGE 18
Generalisability	21	Discuss the generalisability (external validity) of the study results<br>SEE STRENGTHS AND WEAKNESSES OF THE STUDY, PAGE 17
**Other information**		
Funding	22	Give the source of funding and the role of the funders for the present study and, if applicable, for the original study on which the present article is based<br>SEE FUNDING STATEMENT

For peer review only

BORNINBRADFORD

2. Me and My Life wellbeing survey

Me and my life

Please tick the box next to your answer

Please try to answer all the questions. There are no right or wrong answers. We want to know what you think.

Your answers will not be shown to anyone that you know (including mums and dads).

If you do not want to answer a question you can miss it out.

If you need any help, you can ask your teacher or the person who gave you this quiz.

Thank you!

For peer review only

1. Which of these would you most like to eat today?

Pizza 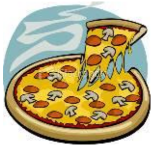 ☐

Curry 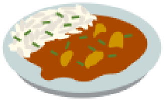 ☐

Pasta with sauce 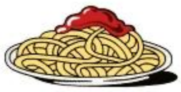 ☐

Sandwich 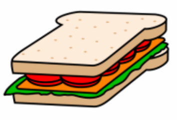 ☐

Where I live

2. How much do you like the home where you live?

I like it a lot 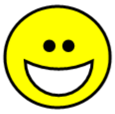 ☐

I like it a bit 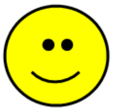 ☐

I don't like it 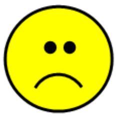 ☐

3. How often do you play in a park?

Very often ☐

Sometimes ☐

Never ☐

For peer review only

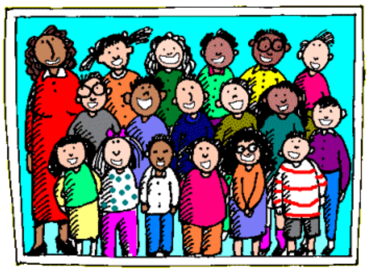

My family and the people I live with

4. How often do you have fun with your family at the weekend?

- All of the time ☐
- Some of the time ☐
- Never ☐

5. How often does your family get along well together?

- All of the time ☐
- Some of the time ☐
- Never ☐

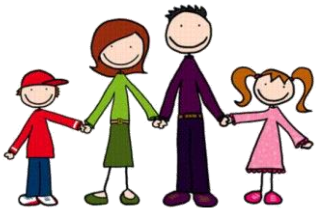

6. How often do you get along with your brothers, sisters and other children you live with?

- All of the time ☐
- Some of the time ☐
- Never ☐

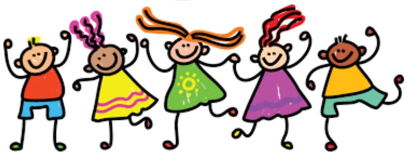

I don't have brothers or sisters or live with other children ☐

For peer review only

7. What languages do you speak with your family at home?

You can tick **more than one** answer if you like.

- English ☐
- Urdu ☐
- Mirpuri/Punjabi ☐
- Slovakian ☐
- Arabic ☐
- Bangla/Bengali ☐
- Hindko ☐
- Pushto ☐
- Other ☐

For peer review only

A bit about me

8. Here is a list of things that some children have.

Do you have each of these things?

		Yes<br>I have it	No<br>I don't have it
Some money to spend on myself	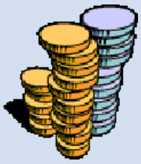	☐	☐
Clothes that you think your friends like	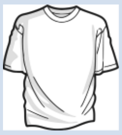	☐	☐
A warm winter coat	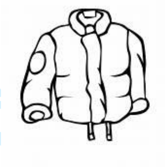	☐	☐
Your own mobile phone	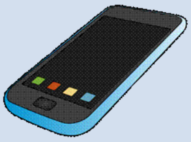	☐	☐
A computer, laptop or tablet with internet at home	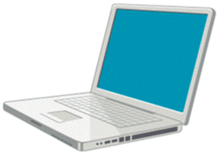	☐	☐
Three meals every day	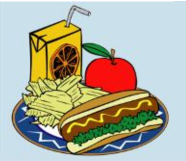	☐	☐

9. Do you have each of these things?

		**Yes** I have it	**No** I don't have it
**Family trips or days out at least once a month, such as to the seaside or a theme park**	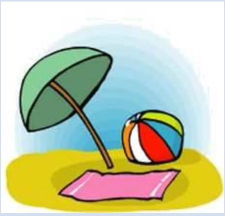	☐	☐
**At least one family holiday away from home each year**	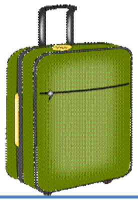	☐	☐

10. Does your home have a garden where you can play?

Yes ☐

No ☐

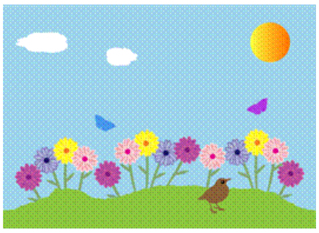

11. Does your family have a car?

Yes ☐

No ☐

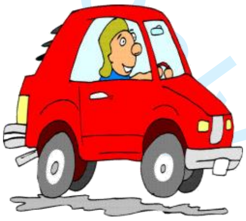

12. Do you have a park near your home where you can play with your friends?

Yes ☐

No ☐

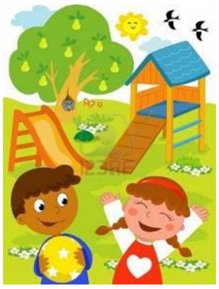

For peer review only

13. How often do you worry about how much money your family has?

All of the time ☐

Some of the time ☐

Never ☐

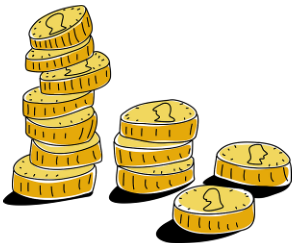

14. How often do you feel happy?

All of the time ☐

Some of the time ☐

Never ☐

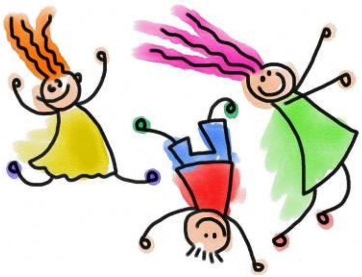

15. How often do you feel sad?

All of the time ☐

Some of the time ☐

Never ☐

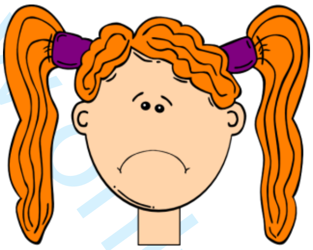

16. How often are you ill or unwell?

All of the time ☐

Some of the time ☐

Never ☐

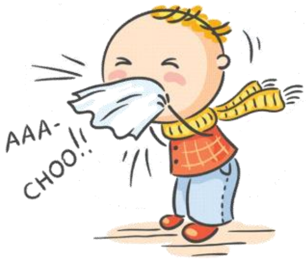

For peer review only

17. How often do you feel healthy?

All of the time ☐

Some of the time ☐

Never ☐

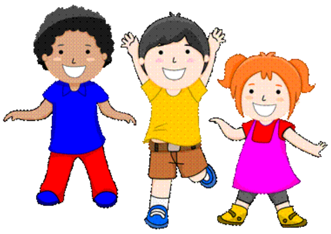

18. What do you do if you are worried about something?

You can tick **more than one** answer if you like.

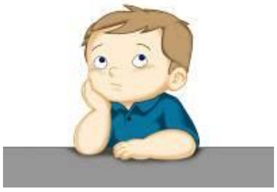

I keep it to myself ☐

I tell a friend ☐

I tell my Mum, Dad or the person who looks after me ☐

I tell a teacher ☐

19. When I find something really hard I can work out what to do next.

All of the time ☐

Some of the time ☐

Never ☐

For peer review only

My friends and other children

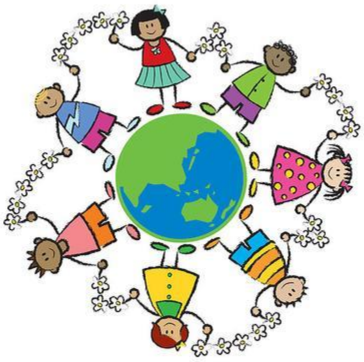

20. How many friends do you have?

Lots ☐

Some ☐

Not many ☐

21. Do you have any best friends?

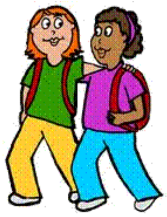

Yes ☐

No ☐

22. How much do you like playing with your friends?

I like it a lot 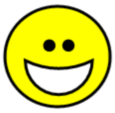 ☐

I like it a bit 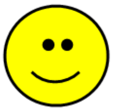 ☐

I don't like it 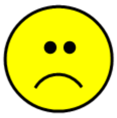 ☐

For peer review only

23. How often do other children bully you?

All of the time ☐

Some of the time ☐

Never ☐

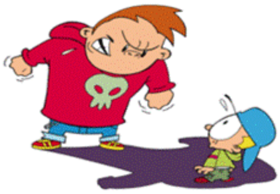

24. How often are you mean to other children at school?

All of the time ☐

Some of the time ☐

Never ☐

25. How often do you feel left out of things by other children?

All of the time ☐

Some of the time ☐

Never ☐

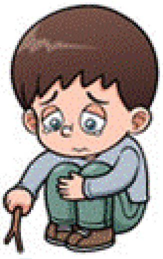

For peer review only

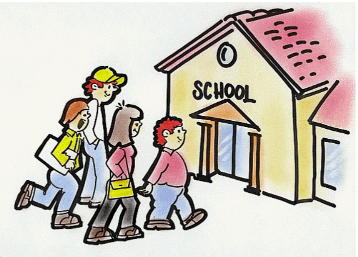

My school

26. How much do you like school?

I like it a lot 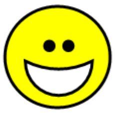 ☐

I like it a bit 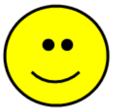 ☐

I don't like it 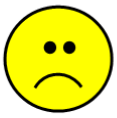 ☐

After school and weekend activities

27. Do you go to any of these clubs or activities after school or at weekends?

		Yes	No
**Sports** (such as football, cricket, swimming, karate)	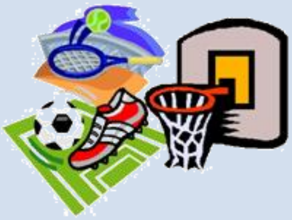	☐	☐
**Music lessons** (such as choir, piano lessons)	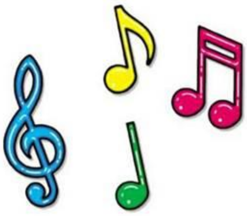	☐	☐
**Dance lessons**	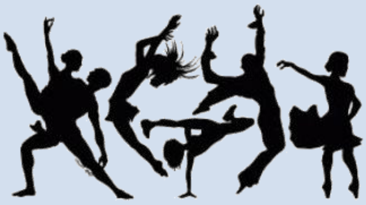	☐	☐
**Any other club** (such as art, drama, film, cooking)		☐	☐

For peer review only

Religion

28. **What is your religion?**

- Christian ☐
- Muslim ☐
- Hindu ☐
- Sikh ☐
- Other religion ☐
- None/No religion ☐
- Don't know ☐

If you are Muslim:

Do you go to Mosque or Madrassa?

- Yes ☐
- No ☐

If yes: How often do you go to Mosque or Madrassa?

- Most days a week ☐
- Some days a week ☐
- Less than once a week ☐

For peer review only

If you are Christian:

Do you go to Church or Sunday school?

Yes ☐

No ☐

If yes: How often do you go to Church or Sunday school?

Most days a week ☐

Some days a week ☐

Less than once a week ☐

If you are Hindu/Sikh:

Do you go to a Temple?

Yes ☐

No ☐

If yes: How often do you go to a Temple?

Most days a week ☐

Some days a week ☐

Less than once a week ☐

For peer review only

If you are another religion:

Do you go to a place of worship?

Yes ☐

No ☐

If yes: How often do you go to a place of worship?

Most days a week ☐

Some days a week ☐

Less than once a week ☐

For peer review only

When I grow up

29. When you grow up, would you like to be:

		Yes, I'd like it a lot	Yes, I'd like it a bit	No, not at all
A cook	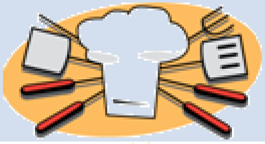	☐	☐	☐
A sports star	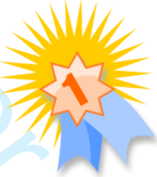	☐	☐	☐
A train driver	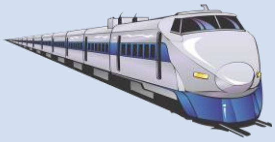	☐	☐	☐
A pop star	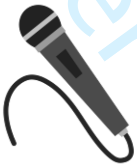	☐	☐	☐
Someone who looks after children at home	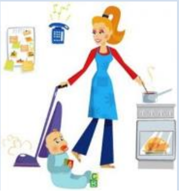	☐	☐	☐
A doctor or a nurse	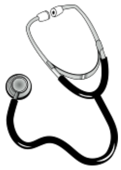	☐	☐	☐
Someone who works in a shop	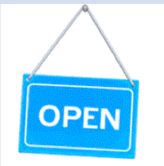	☐	☐	☐
A teacher	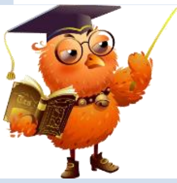	☐	☐	☐
Someone who works with computers	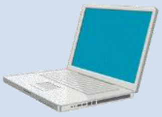	☐	☐	☐

30. What would you most like to be when you grow up?
You can write any job you like!

……

……

31. How much do you like:

		I like it a lot	I like it a bit	I don't like it/ I don't do it
Eating Asian food, such as curry	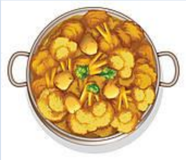	☐	☐	☐
Eating English food, such as fish and chips	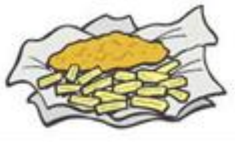	☐	☐	☐
Wearing Asian clothes such as Salwar kameez or kurta	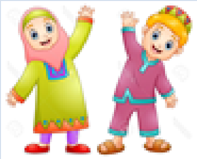	☐	☐	☐
Wearing clothes such as jeans, t-shirts or dresses	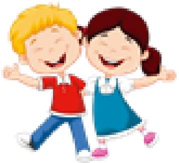	☐	☐	☐

For peer review only

32.

		Yes	No
Do you and your family celebrate Eid?	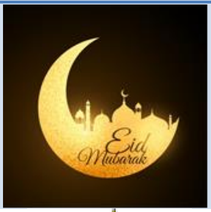	☐	☐
Do you and your family celebrate Christmas?	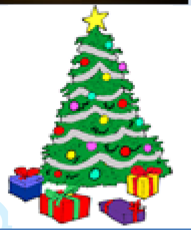	☐	☐

Thank you for your time!

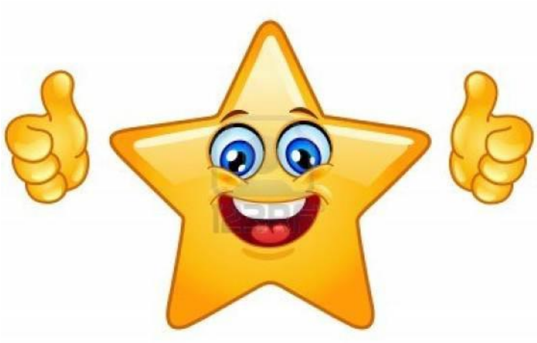

Researcher/Teacher only:
Tick if the child was assisted to complete the questionnaire. ☐

Table S1: Vulnerability across domains of wellbeing

Table S1a: Response to items within 'Home, Family and Family Relationships' domain

For peer review only

How often does your family get along well	Number	Percentage (95% CI)
Never	729	4.8% (4.4%-5.1%)
Some of the time	6,845	44.8% (44.1%-45.6%)
All of the time	7,689	50.4% (49.6%-51.2%)
Missing	378	
Total	15,641	100.0%

Does your home have a garden where you can play?	Number	Percentage (95% CI)
No	2,036	13.2% (12.7%-13.8%)
Yes	13,357	86.8% (86.2%-87.3%)
Missing	248	
Total	15,641	100.0%

Do you have a park near your home where you can play with your friends?	Number	Percentage (95% CI)
No	4,711	30.8% (30.1%-31.5%)
Yes	10,584	69.2% (68.5%-69.9%)
Missing	346	
Total	15,641	100.0%

How often do you play in a park?	Number	Percentage (95% CI)
Never	1,137	7.5% (7.1%-7.9%)
Sometimes	11,701	76.9% (76.2%-77.5%)
Very often	2,386	15.7% (15.1%-16.3%)
Missing	417	
Total	15,641	100.0%

For peer review only

Supplementary Table S1b: Response to items within 'Material Resources' domain

Do you have a warm winter coat?	Number	Percentage (95% CI)
No	1,402	10% (9.5%-10.5%)
Yes	12,594	90% (89.5%-90.5%)
Missing	1,645	
Total	15,641	100.0%

Do you have three meals every day?	Number	Percentage (95% CI)
No	2,243	15.9% (15.3%-16.5%)
Yes	11,891	84.1% (83.5%-84.7%)
Missing	1,507	
Total	15,641	100.0%

Do you have a computer/laptop/tablet with internet at home?	Number	Percentage (95% CI)
No	2,733	19.1% (18.5%-19.8%)
Yes	11,563	80.9% (80.2%-81.5%)
Missing	1,345	
Total	15,641	100.0%

How often do you worry about how much money your family has?	Number	Percentage (95% CI)
Never	5,264	34.1% (33.3%-34.8%)
Some of the time	6,124	39.7% (38.9%-40.4%)
All of the time	4,054	26.3% (25.6%-27%)
Missing	199	
Total	15,641	100.0%

For peer review only

Supplementary Table S1c: Response to items within 'Friends and School' domain

How much do you like your school?	Number	Percentage (95% CI)
Don't like it	2,032	13.1% (12.6%-13.6%)
Like it a bit	4,691	30.2% (29.5%-31%)
Like it a lot	8,797	56.7% (55.9%-57.5%)
Missing	121	
Total	15,641	100.0%

How many friends do you have?	Number	Percentage (95% CI)
Not many	2,257	14.5% (14%-15.1%)
Some	3,647	23.5% (22.8%-24.2%)
Lots	9,623	62% (61.2%-62.7%)
Missing	114	
Total	15,641	100.0%

How often do other children bully you?	Number	Percentage (95% CI)
Never	7,276	47.3% (46.6%-48.1%)
Some of the time	6,413	41.7% (41%-42.5%)
All of the time	1,679	10.9% (10.4%-11.4%)
Missing	273	
Total	15,641	100.0%

How often are you mean to other children at school?	Number	Percentage (95% CI)
Never	10,806	70.9% (70.2%-71.6%)
Some of the time	3,645	23.9% (23.2%-24.6%)
All of the time	794	5.2% (4.9%-5.6%)
Missing	396	
Total	15,641	100.0%

How often do you feel left out by other children?	Number	Percentage (95% CI)
Never	5,598	36.2% (35.5%-37%)
Some of the time	8,129	52.6% (51.8%-53.4%)
All of the time	1,729	11.2% (10.7%-11.7%)
Missing	185	
Total	15,641	100.0%

For peer review only

Supplementary Table S1d: Response to items within 'Subjective Wellbeing' domain

How often do you feel happy?	Number	Percentage (95% CI)
Never	585	3.8% (3.5%-4.1%)
Some of the time	8,306	54.2% (53.5%-55%)
All of the time	6,420	41.9% (41.2%-42.7%)
Missing	330	
Total	15,641	100.0%

How often do you feel sad?	Number	Percentage (95% CI)
Never	3,837	24.9% (24.2%-25.6%)
Some of the time	10,715	69.5% (68.8%-70.2%)
All of the time	863	5.6% (5.2%-6%)
Missing	226	
Total	15,641	100.0%

How often are you ill or unwell?	Number	Percentage (95% CI)
Never	2,216	14.4% (13.8%-14.9%)
Some of the time	11,966	77.6% (76.9%-78.2%)
All of the time	1,247	8.1% (7.7%-8.5%)
Missing	212	
Total	15,641	100.0%

Keep worries to self	Number	Percentage (95% CI)
No	10,655	68.8% (68.1%-69.5%)
Yes	4,828	31.2% (30.5%-31.9%)
Missing	158	
Total	15,641	100.0%

When I find something really hard I can work out what to do next	Number	Percentage (95% CI)
Never	1,299	8.6% (8.1%-9%)
Some of the time	9,429	62.3% (61.5%-63.1%)
All of the time	4,409	29.1% (28.4%-29.9%)
Missing	504	
Total	15,641	100.0%

For peer review only

Table S2: Percentage primary school children with zero, one, or more than one vulnerability in each domain of wellbeing

Home, family, relationships	Number	Percentage (95% CI)
None	8,085	55.9% (55%-56.7%)
One	4,902	33.9% (33.1%-34.6%)
More than one	1,489	10.3% (9.8%-10.8%)
Missing	1,165	
Total	15,641	100.0%

Material resources	Number	Percentage (95% CI)
None	6,331	48.3% (47.5%-49.2%)
One	4,763	36.4% (35.5%-37.2%)
More than one	2,008	15.3% (14.7%-16%)
Missing	2,539	
Total	15,641	100.0%

Friends and school	Number	Percentage (95% CI)
None	5,204	35% (34.3%-35.8%)
One	6,189	41.7% (40.9%-42.5%)
More than one	3,455	23.3% (22.6%-24%)
Missing	793	
Total	15,641	100.0%

Subjective wellbeing	Number	Percentage (95% CI)
None	8,312	57.4% (56.6%-58.2%)
One	4,676	32.3% (31.5%-33.1%)
More than one	1,496	10.3% (9.8%-10.8%)
Missing	1,157	
Total	15,641	100.0%

For peer review only

Supplementary Table S3: Associations of gender with vulnerabilities in child wellbeing domains

Supplementary Table S3a – Home, family, family relations

	Family never get along well together (N = 15,263 non-missing)		No garden where they can play (N = 15,393 non-missing)		No park near home where they can play with friends (N = 15,295 non-missing)		Never plays in park (N = 15,224 non-missing)	
**Gender**	**N**	**Percentage (95% CI)**	**N**	**Percentage (95% CI)**	**N**	**Percentage (95% CI)**	**N**	**Percentage (95% CI)**
Female (N = 7,647)	296	4.0% (3.5%-4.4%)	891	11.8% (11.1%-12.5%)	2225	11.8% (11.1%-12.5%)	456	6.1% (5.6%-6.7%)
Male (N = 7,994)	433	5.6% (5.1%-6.1%)	1145	14.6% (13.8%-15.4%)	2486	14.6% (13.8%-15.4%)	681	8.8% (8.2%-9.4%)

Supplementary Table S3b – Material resources

	No warm winter coat (N = 13,996 non-missing)		Not having three meals a day (N = 14,134 non-missing)		No internet at home (N = 14,296 non-missing)		Worried about money all the time (N = 15,442 non-missing)	
**Gender**	**N**	**Percentage (95% CI)**	**N**	**Percentage (95% CI)**	**N**	**Percentage (95% CI)**	**N**	**Percentage (95% CI)**
Female (N = 7,647)	616	8.9% (8.2%-9.6%)	983	14.0% (13.3%-14.9%)	1383	19.7% (18.8%-20.6%)	1771	23.4% (22.5%-24.4%)
Male (N = 7,994)	786	11.1% (10.4%-11.9%)	1260	17.7% (16.8%-18.6%)	1350	18.6% (17.7%-19.5%)	2283	28.9% (28.0%-30.0%)

For peer review only - http://bmjopen.bmj.com/site/about/guidelines.xhtml

Supplementary Table S3c – Friends and school

	Does not like school (N = 15,520 non-missing)		Not many friends (N = 15,527 non-missing)		Bullied some or all of the time (N = 15,368 non-missing)		Mean to others all the time (N = 15,245 non-missing)		Feel left out all the time (N = 15,456 non-missing)	
**Gender**	**N**	**Percentage (95% CI)**	**N**	**Percentage (95% CI)**	**N**	**Percentage (95% CI)**	**N**	**Percentage (95% CI)**	**N**	**Percentage (95% CI)**
Female (N = 7,647)	606	8.0% (7.4%-8.6%)	1269	16.7% (15.9%-17.6%)	4192	55.8% (54.7%-56.9%)	252	3.4% (3.0%-3.8%)	916	12.1% (11.4%-12.8%)
Male (N = 7,994)	1426	18.0% (17.1%-18.8%)	988	12.5% (11.8%-13.2%)	3900	49.7% (48.6%-50.8%)	542	7.0% (6.4%-7.6%)	813	10.3% (9.7%-11.0%)

Supplementary Table S3d – Subjective wellbeing

	Never happy (N = 15,331 non-missing)		Always sad (N = 15,415 non-missing)		Always ill or unwell (N = 15,429 non-missing)		Keeps worries to self (N = 15,483 non-missing)		Cannot work out what to do when things are hard (N = 15,137 non-missing)	
**Gender**	**N**	**Percentage (95% CI)**	**N**	**Percentage (95% CI)**	**N**	**Percentage (95% CI)**	**N**	**Percentage (95% CI)**	**N**	**Percentage (95% CI)**
Female (N = 7,647)	179	2.4% (2.1%-2.8%)	412	5.5% (5.0%-6.0%)	581	7.7% (7.1%-8.3%)	2166	28.5% (27.5%-29.6%)	555	7.5% (6.9%-8.1%)
Male (N = 7,994)	406	5.2% (4.7%-5.7%)	451	5.7% (5.2%-6.3%)	666	8.5% (7.9%-9.1%)	2662	33.7% (32.7%-34.8%)	744	9.6% (9.0%-10.3%)

For peer review only - http://bmjopen.bmj.com/site/about/guidelines.xhtml

Supplementary Table S3e: Associations of gender for each child wellbeing domain

	Home, family and family relationships (N = 14,476 non-missing)		Material resources (N = 13,102 non-missing)		Friends and school (N = 14,848 non-missing)		Subjective wellbeing (N = 14,484 non-missing)	
**Gender**	**N**	**Percentage (95% CI)**	**N**	**Percentage (95% CI)**	**N**	**Percentage (95% CI)**	**N**	**Percentage (95% CI)**
Female (N = 7,647)	7,117	41.7% (40.6%-42.9%)	6,520	48.8% (47.6%-50.0%)	7,284	65.0% (63.9%-66.1%)	7,148	39.5% (38.4%-40.7%)
Male (N = 7,994)	7,359	46.5% (45.3%-47.6%)	6,582	54.5% (53.3%-55.7%)	7,564	64.9% (63.8%-66.0%)	7,336	45.6% (44.5%-46.7%)

For peer review only

Supplementary Table S4: Associations of ethnicity with vulnerabilities in child wellbeing domains

Supplementary Table S4a – Home, family, family relations

	Family never get along well together (N = 15,263 non-missing)		No garden where they can play (N = 15,393 non-missing)		No park near home where they can play with friends (N = 15,295 non-missing)		Never plays in park (N = 15,224 non-missing)	
**Ethnicity**	**N**	**Percentage (95% CI)**	**N**	**Percentage (95% CI)**	**N**	**Percentage (95% CI)**	**N**	**Percentage (95% CI)**
Pakistani (N = 7,031)	278	4.1% (3.6%-4.6%)	837	12.1% (11.4%-12.9%)	2243	32.7% (31.6%-33.9%)	470	6.9% (6.3%-7.5%)
White British (N = 4,247)	265	6.3% (5.6%-7.1%)	562	13.4% (12.4%-14.5%)	1247	29.9% (28.6%-31.3%)	374	9.0% (8.2%-9.9%)
Mixed (N = 900)	55	6.3% (4.9%-8.1%)	134	15.0% (12.8%-17.5%)	254	28.7% (25.8%-31.8%)	75	8.5% (6.8%-10.5%)
White Other (N = 707)	24	3.5% (2.3%-5.2%)	125	18.0% (15.3%-21%)	188	27.1% (23.9%-30.6%)	46	6.7% (5.1%-8.9%)
Bangladeshi (N = 471)	18	3.9% (2.5%-6.2%)	73	15.8% (12.8%-19.5%)	154	33.6% (29.4%-38.0%)	32	6.9% (4.9%-9.6%)
Indian (N = 357)	17	4.9% (3.0%-7.7%)	64	18.1% (14.4%-22.4%)	121	34.4% (29.6%-39.5%)	25	7.2% (4.9%-10.4%)
Black/ Black British (N = 277)	5	1.8% (0.8%-4.3%)	49	18.0% (13.9%-23%)	62	22.7% (18.1%-28.1%)	19	7.0% (4.5%-10.7%)
Gypsy/ Irish traveller (N = 190)	13	7.3% (4.3%-12.2%)	28	15.2% (10.7%-21.2%)	54	29.2% (23.1%-36.1%)	18	9.9% (6.3%-15.2%)
Other (N = 425)	14	3.4% (2.0%-5.7%)	76	18.1% (14.7%-22.1%)	129	31.5% (27.2%-36.1%)	35	8.5% (6.1%-11.5%)

Supplementary Table S4b – Material resources

Ethnicity	No warm winter coat (N = 13,996 non-missing) N	No warm winter coat Percentage (95% CI)	Not having three meals a day (N = 14,134 non-missing) N	Not having three meals a day Percentage (95% CI)	No internet at home (N = 14,296 non-missing) N	No internet at home Percentage (95% CI)	Worried about money all the time (N = 15,442 non-missing) N	Worried about money all the time Percentage (95% CI)
Pakistani (N = 7,031)	609	9.8% (9.1%-10.6%)	1116	17.9% (16.9%-18.8%)	1141	18.0% (17.0%-18.9%)	2031	29.3% (28.2%-30.3%)
White British (N = 4,247)	432	11.1% (10.1%-12.1%)	470	11.9% (10.9%-12.9%)	880	22.3% (21.0%-23.6%)	912	21.6% (20.4%-22.9%)
Mixed (N = 900)	76	9.6% (7.8%-11.9%)	145	18.0% (15.5%-20.8%)	159	19.7% (17.1%-22.6%)	223	25.3% (22.5%-28.3%)
White Other (N = 707)	55	8.9% (6.9%-11.4%)	90	14.4% (11.8%-17.3%)	113	17.7% (14.9%-20.8%)	204	29.4% (26.1%-32.9%)
Bangladeshi (N = 471)	31	7.5% (5.3%-10.5%)	65	16.1% (12.8%-20.0%)	74	17.6% (14.2%-21.5%)	128	27.6% (23.7%-31.8%)
Indian (N = 357)	31	9.6% (6.8%-13.3%)	67	20.4% (16.4%-25.1%)	47	14.3% (10.9%-18.5%)	88	24.9% (20.7%-29.7%)
Black/ Black British (N = 277)	20	8.0% (5.2%-12.1%)	40	15.9% (11.9%-20.9%)	36	13.7% (10%-18.4%)	74	26.8% (21.9%-32.3%)
Gypsy/ Irish traveller (N = 190)	15	9.4% (5.7%-15%)	32	19.8% (14.3%-26.6%)	43	25.3% (19.3%-32.4%)	73	39.0% (32.3%-46.2%)
Other (N = 425)	33	8.9% (6.4%-12.2%)	56	14.9% (11.6%-18.8%)	81	21.3% (17.5%-25.7%)	101	24.4% (20.5%-28.8%)

For peer review only

Supplementary Table S4c – Friends and school

	Does not like school (N = 15,520 non-missing)		Not many friends (N = 15,527 non-missing)		Bullied some or all of the time (N = 15,368 non-missing)		Mean to others all the time (N = 15,245 non-missing)		Feel left out all the time (N = 15,456 non-missing)	
**Ethnicity**	**N**	**Percentage (95% CI)**	**N**	**Percentage (95% CI)**	**N**	**Percentage (95% CI)**	**N**	**Percentage (95% CI)**	**N**	**Percentage (95% CI)**
Pakistani (N = 7,031)	889	12.7% (12.0%-13.5%)	1029	14.7% (13.9%-15.6%)	3630	52.6% (51.4%-53.7%)	400	5.8% (5.3%-6.4%)	790	11.4% (10.6%-12.1%)
White British (N = 4,247)	652	15.4% (14.4%-16.6%)	662	15.7% (14.6%-16.8%)	2253	53.8% (52.3%-55.3%)	167	4.0% (3.5%-4.7%)	486	11.6% (10.6%-12.6%)
Mixed (N = 900)	128	14.4% (12.2%-16.8%)	113	12.7% (10.6%-15%)	459	51.9% (48.6%-55.1%)	41	4.7% (3.5%-6.3%)	98	11.0% (9.1%-13.2%)
White Other (N = 707)	69	9.8% (7.8%-12.3%)	106	15.2% (12.7%-18%)	328	47.1% (43.4%-50.8%)	44	6.4% (4.8%-8.5%)	74	10.6% (8.5%-13.1%)
Bangladeshi (N = 471)	54	11.5% (8.9%-14.8%)	53	11.3% (8.7%-14.5%)	235	51.1% (46.5%-55.6%)	24	5.2% (3.5%-7.6%)	40	8.5% (6.3%-11.4%)
Indian (N = 357)	33	9.3% (6.7%-12.8%)	46	13.0% (9.9%-16.9%)	183	52.0% (46.8%-57.2%)	15	4.3% (2.6%-7.0%)	31	8.8% (6.3%-12.3%)
Black/ Black British (N = 277)	32	11.6% (8.3%-15.9%)	23	8.3% (5.6%-12.2%)	144	52.9% (47%-58.8%)	14	5.2% (3.1%-8.5%)	24	8.8% (6.0%-12.8%)
Gypsy/ Irish traveller (N = 190)	20	10.7% (7.0%-16.0%)	30	16.0% (11.4%-21.9%)	79	42.2% (35.4%-49.4%)	18	9.9% (6.3%-15.2%)	21	11.3% (7.5%-16.7%)
Other (N = 425)	53	12.6% (9.8%-16.2%)	60	14.3% (11.3%-18%)	233	56.3% (51.5%-61.0%)	24	5.8% (3.9%-8.5%)	40	9.6% (7.1%-12.8%)

For peer review only

Supplementary Table S4d – Subjective wellbeing

	Never happy (N = 15,331 non-missing)		Always sad (N = 15,415 non-missing)		Always ill or unwell (N = 15,429 non-missing)		Keeps worries to self (N = 15,483 non-missing)		Cannot work out what to do when things are hard (N = 15,137 non-missing)	
**Ethnicity**	**N**	**Percentage (95% CI)**	**N**	**Percentage (95% CI)**	**N**	**Percentage (95% CI)**	**N**	**Percentage (95% CI)**	**N**	**Percentage (95% CI)**
Pakistani (N = 7,031)	281	4.1% (3.7%-4.6%)	406	5.9% (5.3%-6.4%)	622	9% (8.3%-9.7%)	2222	31.9% (30.8%-33%)	570	8.4% (7.7%-9.1%)
White British (N = 4,247)	168	4% (3.5%-4.7%)	223	5.3% (4.7%-6%)	305	7.2% (6.5%-8.1%)	1268	30.2% (28.8%-31.6%)	372	9% (8.2%-9.9%)
Mixed (N = 900)	38	4.3% (3.2%-5.9%)	55	6.2% (4.8%-8%)	73	8.3% (6.6%-10.3%)	281	31.5% (28.6%-34.7%)	88	10.1% (8.2%-12.2%)
White Other (N = 707)	26	3.8% (2.6%-5.5%)	43	6.2% (4.6%-8.2%)	50	7.2% (5.5%-9.4%)	210	30% (26.8%-33.5%)	69	10.1% (8.1%-12.7%)
Bangladeshi (N = 471)	14	3.1% (1.8%-5.1%)	21	4.5% (3%-6.9%)	45	9.7% (7.3%-12.8%)	139	29.8% (25.8%-34.1%)	22	4.8% (3.2%-7.1%)
Indian (N = 357)	6	1.7% (0.8%-3.8%)	20	5.7% (3.7%-8.7%)	22	6.3% (4.2%-9.4%)	112	31.6% (27%-36.7%)	23	6.7% (4.5%-9.9%)
Black/ Black British (N = 277)	6	2.2% (1%-4.8%)	16	5.8% (3.6%-9.3%)	19	6.9% (4.4%-10.5%)	79	28.6% (23.6%-34.2%)	15	5.6% (3.4%-9.1%)
Gypsy/ Irish traveller (N = 190)	6	3.3% (1.5%-7.1%)	13	7% (4.1%-11.7%)	16	8.6% (5.3%-13.5%)	60	32.3% (25.9%-39.3%)	27	15.2% (10.6%-21.2%)
Other (N = 425)	14	3.4% (2%-5.6%)	23	5.5% (3.7%-8.2%)	36	8.6% (6.2%-11.7%)	135	32.5% (28.1%-37.1%)	38	9.3% (6.8%-12.5%)

For peer review only

Supplementary Table S7: Associations of Index of Multiple Deprivation for each child wellbeing domain

IMD 2019 Quintile (1 = most materially deprived)	Home, family and family relationships (N = 5,703 non-missing)		Material resources (N = 5,153 non-missing)		Friends and school (N = 5,869 non-missing)		Subjective wellbeing (N = 5,706 non-missing)	
	N	Percentage (95% CI)	N	Percentage (95% CI)	N	Percentage (95% CI)	N	Percentage (95% CI)
1 (N = 4,334)	1817	45.4% (43.8%-46.9%)	1907	53.5% (51.9%-55.1%)	2713	65.7% (64.2%-67.1%)	1726	43.1% (41.6%-44.6%)
2 (N = 1,303)	511	42.0% (39.2%-44.8%)	546	48.5% (45.6%-51.5%)	797	64.0% (61.3%-66.6%)	482	39.5% (36.8%-42.3%)
3 (N = 308)	103	35.5% (30.0%-41.0%)	119	43.9% (38.0%-49.8%)	182	61.1% (55.5%-66.6%)	113	39.6% (34.0%-45.3%)
4 (N = 139)	50	38.5% (30.1%-46.8%)	49	37.1% (28.9%-45.4%)	80	60.6% (52.3%-68.9%)	47	35.1% (27.0%-43.2%)
5 (N = 59)	23	39.0% (26.5%-51.4%)	19	33.9% (21.5%-46.3%)	33	56.9% (44.2%-69.6%)	19	32.8% (20.7%-44.8%)

BMJ Open

Vulnerabilities in child wellbeing among primary school children: a cross-sectional study in Bradford, UK

Journal:	*BMJ Open*
Manuscript ID	bmjopen-2021-049416.R1
Article Type:	Original research
Date Submitted by the Author:	07-Dec-2021
Complete List of Authors:	Pickett, Kate; University of York<br>Ajebon, Mildred; University of York<br>Hou, Bo; Bradford Institute for Health Research<br>Kelly, Brian; Bradford Institute for Health Research<br>Bird, Philippa K; Leeds Teaching Hospitals NHS Trust<br>Dickerson, Josie; Bradford Institute for Health Research<br>Shire, Katy; Bradford Institute for Health Research<br>Mon-Williams, Mark; University of Leeds, Psychology<br>Small, Neil; University of Bradford<br>McEachan, Rosemary; Bradford Institute for Health Research<br>Wright, John; Bradford Institute for Health Research<br>Lawlor, Deborah; University of Bristol
**Primary Subject Heading**:	Epidemiology
Secondary Subject Heading:	Public health
Keywords:	COVID-19, Community child health < PAEDIATRICS, SOCIAL MEDICINE

SCHOLARONE™
Manuscripts

I, the Submitting Author has the right to grant and does grant on behalf of all authors of the Work (as defined in the below author licence), an exclusive licence and/or a non-exclusive licence for contributions from authors who are: i) UK Crown employees; ii) where BMJ has agreed a CC-BY licence shall apply, and/or iii) in accordance with the terms applicable for US Federal Government officers or employees acting as part of their official duties; on a worldwide, perpetual, irrevocable, royalty-free basis to BMJ Publishing Group Ltd ("BMJ") its licensees and where the relevant Journal is co-owned by BMJ to the co-owners of the Journal, to publish the Work in this journal and any other BMJ products and to exploit all rights, as set out in our licence.

The Submitting Author accepts and understands that any supply made under these terms is made by BMJ to the Submitting Author unless you are acting as an employee on behalf of your employer or a postgraduate student of an affiliated institution which is paying any applicable article publishing charge ("APC") for Open Access articles. Where the Submitting Author wishes to make the Work available on an Open Access basis (and intends to pay the relevant APC), the terms of reuse of such Open Access shall be governed by a Creative Commons licence – details of these licences and which Creative Commons licence will apply to this Work are set out in our licence referred to above.

Other than as permitted in any relevant BMJ Author's Self Archiving Policies, I confirm this Work has not been accepted for publication elsewhere, is not being considered for publication elsewhere and does not duplicate material already published. I confirm all authors consent to publication of this Work and authorise the granting of this licence.

For peer review only

Vulnerabilities in child wellbeing among primary school children: a cross-sectional study in Bradford, UK

Kate E Pickett[1*](0000-0002-8066-8507), Mildred Ajebon[1](0000-0001-5353-8080), Bo Hou[2](0000-0001-5337-6560), Brian Kelly[2](0000-0003-1834-2992), Philippa K Bird[3](0000-0002-9601-7979), Josie Dickerson[2](0000-0003-0121-3406), Katy Shire[2] (0000-0002-2093-181X) Mark Mon-Williams[4] (0000-0001-7595-8545), Neil Small[5] (0000-0002-4426-3596), Rosemary McEachan[2] (0000-0003-1302-6675), John Wright[2] (0000-0001-9572-7293) Deborah A Lawlor[6-8] (0000-0002-6793-2262)

***Corresponding author: kate.pickett@york.ac.uk**

Addresses:

[1]Department of Health Sciences, Seebohm Rowntree Building Area 2, University of York, Heslington, York, YO105DD

[2]Bradford Institute for Health Research, Bradford Teaching Hospitals NHS Foundation Trust, Bradford Royal Infirmary, Duckworth Lane, Bradford, BD9 6RJ, UK

[3]Leeds Teaching Hospitals NHS Foundation Trust, St. James's University Hospital, Beckett Street, Leeds, West Yorkshire, LS9 7TF

[4] School of Psychology, University of Leeds, Leeds LS2 9JT

[5] Faculty of Health Studies, University of Bradford, West Yorkshire BD7 1DP UK

[6]MRC Integrative Epidemiology Unit, University of Bristol, Oakfield House, Oakfield Grove, Bristol, BS8 2BN, UK

[7]Population Health Science, Bristol Medical School, University of Bristol, UK

[8]Bristol National Institute for Health Research Biomedical Research Centre, UK

For peer review only - http://bmjopen.bmj.com/site/about/guidelines.xhtml

ABSTRACT

Objective: To describe the prevalence of factors related to wellbeing among primary school children in a deprived multi-ethnic community.

Design and participants: Cross-sectional survey of 15,641 children aged 7-10 years in Born in Bradford's Primary School Years study: whole-classroom samples in 89 Bradford primary schools between 2016 and 2019.

Main outcome measures: Prevalence estimates by ethnicity (%, 95% CI) of single and multiple vulnerabilities in child wellbeing within and across four domains (Home, Family, Relationships; Material Resources; Friends and School; Subjective Wellbeing).

Results Only 10% of children had no vulnerabilities in any domain of wellbeing; 10% had one or more vulnerabilities in all four domains. The highest prevalence estimates were for being bullied some or all of the time (52.7%, 51.9 to 53.4%), keeping worries to oneself (31.2%, 30.5 to 31.9%), having no park near home (30.8%, 30.1 to 31.5%) and worrying all the time about how much money their family has (26.3%, 25.6 to 27%).

Boys were consistently significantly more likely than girls to report all of the vulnerabilities in the Home, Family and Family Relationships domain, and the majority of indicators in the other domains, and in all domains except Friends and School, boys were significantly more likely to have at least one vulnerability. Girls were significantly more likely to report not having many friends (16.7%, 95% CI: 15.9 to 17.6% vs. 12.5%, 95% CI: 11.8 to 13.2%), being bullied some or all of the time (55.8%, 95% CI: 54.7 to 56.9% vs. 49.7%, 95% CI: 48.6 to 50.8%) and feeling left out all the time (12.1%, 95% CI: 11.4 to 12.8%) vs. (10.3%, 95% CI: 9.7 to 11.0%).

Variations in vulnerabilities by ethnicity were complex, with children in Black, Asian and Minority Ethnic groups sometimes reporting more vulnerabilities and sometimes fewer than White British children. For example, compared to children of Pakistani heritage, White British children were more likely to say that their family never gets along well (6.3%,5.6 to 7.1% vs. 4.1%,3.6 to 4.6%) and to have no access to the internet at home (22.3%,21 to 23.6% vs. 18%,17 to 18.9%). Children with Pakistani heritage were more likely than White British

For peer review only - http://bmjopen.bmj.com/site/about/guidelines.xhtml

children to say they had no park near their home where they can play with friends (32.7%,31.6 to 33.9% vs. 29.9%,28.6 to 31.3%), to report not having three meals a day (17.9%,16.9 to 18.8% vs. 11.9%,10.9 to 12.9%) and to worry all the time about how much money their families have (29.3%,28.2 to 30.3%) vs. (21.6%,20.4 to 22.9%). Gypsy/Irish Traveller children were less likely than White British children to say they were bullied some or all of the time (42.2%,35.4 to 49.4% vs. 53.8%,52.3 to 55.3%), but more likely to say they were mean to others all the time (9.9%,6.3 to 15.2% vs. 4%,3.5 to 4.7%) and can never work out what to do when things are hard (15.2%,10.6 to 21.2% vs. 9%, 8.2 to 9.9%).

We considered six vulnerabilities to be of particular concern during the Covid-19 pandemic and associated national and local lockdowns: family never gets along well together; no garden where child can play; no nearby park where they can play; not having three meals a day; no internet at home; worried about money all the time. Pre-pandemic, 37.4% (36.6 to 38.3%) of Bradford children had one of these vulnerabilities and a further 29.6% (28.9 to 30.4%) had more than one.

Conclusions

Although most primary school children aged 7-10 in our study had good levels of wellbeing on most indicators across multiple domains, fewer than 10% had no vulnerabilities at all, a worrying 10% had at least one vulnerability in all the four domains we studied and two thirds had vulnerabilities of particular concern during the Covid-19 lockdowns.

KEYWORDS

Children; subjective wellbeing; poverty; health inequalities; ethnicity; social determinants of health; cohorts; Born in Bradford; Covid-19

Summary

Strengths and limitations of this study

- A uniquely large study of contemporary child wellbeing in the UK with a high response rate and including a broad group of ethnicities, generalisable to other UK cities with high levels of ethnic diversity and deprivation
- Subjective measures of a wide range of experiences capture how children perceive their lives
- A descriptive study of prevalence of vulnerabilities in wellbeing, unable to examine causes

For peer review only

For peer review only - http://bmjopen.bmj.com/site/about/guidelines.xhtml

Introduction

In 2007, the UK ranked bottom of 21 countries in UNICEF's first report on child wellbeing in rich countries,[1] and from 2009 through 2019 the Good Childhood inquiry[2] and subsequent reports from the Children's Society[3] found increasing levels of unhappiness and mental distress among children and young people in the UK. The State of the Nation 2019 report on children and young people's wellbeing suggested a need to "understand wellbeing across different groups" and to "use a range of measures to understand their experience."[4]

Wellbeing declines as children and young people grow older[4] but little is known about wellbeing in primary school aged children in the UK as previous studies, including the Good Childhood surveys, Understanding Society and HeadStart, included children over 10 years old only. The impact of ethnicity on child wellbeing at any age is unclear and most previous UK studies have been unable to examine wellbeing across a wide range of ethnicities.[4]

The global Covid-19 pandemic and restrictions, such as lockdowns that closed schools, heightened concerns about child wellbeing across the world. The prevalence of pre-existing child vulnerabilities and risk factors that could potentially require mitigation and targeted intervention during and after the pandemic has been a concern, particularly in poor, ethnically diverse urban settings.[5]

To provide evidence to meet these knowledge gaps, we examined child wellbeing among primary school children, aged 7 to 10 years, in a city in the North of England. Bradford is the fifth largest metropolitan district in England with one of the youngest and most ethnically diverse populations.[6] Bradford also has some of the highest levels of poverty and ill-health in England; almost a quarter of Bradford children live in poverty and 24% are obese at age 10/11.[7] The aim of this paper is to describe the prevalence of vulnerabilities in child wellbeing by ethnicity and gender in a deprived community. Whilst this research was undertaken in the city of Bradford and has been used to support local policy during the Covid-19 pandemic,[8] we believe the results have wider relevance for deprived multi-ethnic urban settings.

Methods

Setting and Participants

For peer review only - http://bmjopen.bmj.com/site/about/guidelines.xhtml

Born in Bradford (BiB) is a cohort study of 13,500 children born between 2007-2011.[9 10] Between 2016 and 2019, BiB administered the Primary School Years study as a cross-sectional survey of wellbeing of children aged 7-10 years in 89 Bradford schools . We surveyed whole classrooms, including children previously recruited to the ongoing BiB cohort study as well as non-BiB children. The study protocol and detailed methods of school recruitment are described elsewhere[11 12] and the SROBE statement can be found in Supplementary Materials: 1. Schools were given information sheets and opt-out consent forms to give to parents of children in eligible year groups ahead of the school visit; one school asked for opt-in consent to be used and this was accommodated. Fewer than 5% of parents opted out of the study.

Design and Procedures

Ethical approval was obtained from the NHS Health Research Authority's Yorkshire and the Humber - Bradford Leeds Research Ethics Committee (reference: 16/YH/0062) on the 24th March 2016.

The wellbeing survey ("Me and My Life") was developed for this study (see Supplementary Materials: 2), drawing on questions asked of children in the Millennium Cohort Study,[13] the International Survey of Children's Wellbeing[14] and the Avon Longitudinal Study of Parents and Children.[15] The survey aimed to assess children's wellbeing in multiple domains, including: happiness, health, material wellbeing, relationships with family and friends, school experience, neighbourhood, aspirations and acculturation. 'Think aloud' testing with a small group of children was used to check understanding and face validity. During administration, whole classrooms completed the survey at the same time, supported by three research facilitators.

Schools provided class lists, including date of birth, gender and ethnicity. Child ethnicity is reported by parents on registration with a school. The ethnicity information provided from school records contained 192 categories. These were re-coded into 18 broad groups in line with the 2011 census categorisation of ethnic groups in the UK and for the analyses presented here collapsed into 10 categories: Pakistani, Bangladeshi, Indian, Black/Black British, White British, Mixed, Gypsy/Irish Traveller, Other White, Other, and Unknown. Where ethnicity was missing it was possible to supplement, for BiB children only, with data held by BiB, as BiB requests regular data updates of child data from the Local Authority (n=311).

For peer review only - http://bmjopen.bmj.com/site/about/guidelines.xhtml

To assess vulnerabilities in wellbeing, we grouped questions within four domains: (1) Home, Family and Family relationships, (2) Material Resources, (3) Friends and School, (4) Subjective (self-reported) Wellbeing. Within each domain, we defined 'vulnerabilities' on (a) the basis of research literature showing these to be well-established childhood risk factors for long-term health, wellbeing, educational attainment, and social mobility and (b) subsequent consultation with the vulnerabilities workstream of the Bradford Institute for Health Research Covid-19 Scientific Advisory Group (BIHR C-SAG) (https://www.bradfordresearch.nhs.uk/vulnerable-groups/). The risk factors categorised as vulnerabilities include, by domain:

• **Home, Family, Family Relationships**: family never gets along well together, no garden where child can play, no nearby park where they can play, never plays in park

• **Material Resources**: no warm winter coat, not having three meals a day, no internet at home, worries about money all the time

• **Friends and School**: doesn't like school, not many friends, bullied some or all of the time, mean to others all the time, feel left out all the time

• **Subjective Wellbeing**: never happy, always sad, ill/unwell all of the time, keeps worries to self, can never work out what to do when things are hard

Data Analysis

Socio-demographics and the prevalence of vulnerabilities in child wellbeing are presented as counts and/or percentages with 95% confidence intervals, for all children and by gender and ethnicity. We also describe the number and percent of children with multiple vulnerabilities within and across domains of wellbeing by gender and ethnicity. Children with missing data for any answer are excluded from the relevant analyses and number of missing reported in figures and tables.

Results

Figure 1 shows the number of schools and children included in the study.

Table 1: Socio-demographic characteristics of 15,641 Bradford primary school children

	**Number**	**Percent**

For peer review only - http://bmjopen.bmj.com/site/about/guidelines.xhtml

**Gender**		
Female	7647	48.9%
Male	7994	51.1%
**Age (years)**		
6	2	<0.01%
7	5264	33.7%
8	6901	44.1%
9	3175	20.3%
10	293	1.9%
11	1	<0.01%
12	3	<0.01%
13	2	<0.01%
Missing		
**Ethnicity**		
Pakistani	7031	45.0%
Bangladeshi	471	3.0%
Indian	357	2.3%
Black/Black British	277	1.8%
White British	4247	27.1%
Mixed	900	5.7%
Gypsy/Irish Traveller	190	1.2%
White Other	707	4.5%
Other	425	2.7%
Unknown	1036	6.6%

Table 1 shows the socio-demographics of the 15,641 survey respondents. Figure 2 presents the prevalence of each vulnerability across all domains for all children (see also Supplementary Materials, Table S1); Figure 3 presents the percentage of all children who have zero, one and more than one vulnerability within each domain (see also Supplementary Materials, Table S2).

Although the prevalence of vulnerability varied by item and domain, within each domain there is an item with notably high prevalence, with more than one in four children reporting vulnerability: no park near home (30.8%, 95% CI: 30.1-31.5%); worries about money all the

For peer review only - http://bmjopen.bmj.com/site/about/guidelines.xhtml

time (26.3%, 95% CI: 25.6-27.0%); bullied some or all of the time (52.7%, 95% CI: 51.9-53.4%); and keeping worries to self (31.2%, 95% CI: 30.5-31.9%). We considered six vulnerabilities in child wellbeing would be of particular concern during lockdowns and school closures associated with the Covid-19 pandemic: family never gets along well together; no garden where child can play; no nearby park where they can play; not having three meals a day; no internet at home; worried about money all the time. Before the pandemic 37.4% (36.6-38.3%) of children had one of these vulnerabilities and a further 29.6% (28.9-30.4%) had more than one.

Although the majority of children had no vulnerabilities in the Home, Family, Relationships domain (55.9%, 95% CI: 55.0-56.7%) and the Subjective Wellbeing domain (57.4%, 95% CI: 56.6-58.2%), the majority of children had one or more vulnerabilities in the Material Resources, and Friends and School domains. Only 10% (add 95% CI) of children had no vulnerabilities in any domain of wellbeing. Figure 4 is a Venn diagram that shows how vulnerabilities overlap across domains. 1,494 (10%) children had no vulnerabilities. Each oval in the Venn diagram includes all the children who had one or more vulnerabilities within that domain; so, for example, 2% of children had vulnerabilities in Home, Family, Relationships *and* Subjective Wellbeing but not in other domains. 1,519 children (10%) had one or more vulnerabilities in all four domains.

Variation by gender

Boys were consistently significantly more likely to report all of the vulnerabilities in the Home, Family and Family Relationships domain, although the gender differences were not large (see Supplementary Materials: Table 3 for full results, including 95% confidence intervals, for all domains by gender). There were no gender differences in having access to the internet at home, but boys were significantly more likely to report not having a winter coat (11.1%, 95% CI: 10.4-11.9% vs 8.9%, 95% CI: 8.2-9.6%), not having three meals a day (17.7%, 95% CI: 16.8-18.6%) vs 14.0%, 95% CI: 13.3-14.9%) and worrying about money all the time (28.9%, 95% CI: 28.0-30.0%) vs. (23.4%, 95% CI: 22.5-24.4%). Within the Friends and School domain, boys were significantly more likely to not like school (18.0%, 95% CI: 17.1-18.8% vs. 8%, 95% CI: 7.4-8.6%) and to be mean to others all the time (7.0%, 95% CI: 6.4-7.6% vs. 3.4%, 95% CI: 3.0-3.8%), while girls were significantly more likely to report not having many friends (16.7%, 95% CI: 15.9-17.6% vs. 12.5%, 95% CI: 11.8-13.2%), being bullied some or all of the time (55.8%, 95% CI: 54.7-56.9% vs. 49.7%, 95% CI: 48.6-

For peer review only - http://bmjopen.bmj.com/site/about/guidelines.xhtml

50.8%) and feeling left out all the time (12.1%, 95% CI: 11.4-12.8% vs. 10.3%, 95% CI: 9.7-11.0%). Within the Subjective Wellbeing domain there were no gender differences in being always sad or always ill or unwell, but boys were significantly likely to report never being happy (5.2%, 95% CI: 4.7-5.7% vs. 2.4%, 95% CI: 2.1-2.8%), keeping worries to themselves (33.7%, 95% CI: 32.7-34.8% vs. 28.5%, 95% CI: 27.5-29.6%) and not being about to work out what to do when things are hard (9.6%, 95% CI: 9.0-10.3% vs. 7.5%, 95% CI: 6.9-8.1%). In all domains except for Friends and School, boys were significantly more likely to have at least one vulnerability, compared to girls.

Variation by ethnicity

Figures 5-8 shows the percentages (and 95% confidence intervals) of primary school children with at least one vulnerability within each of the domains of child wellbeing, by ethnicity. Full results are given in Supplementary Materials, Tables S4a-4d.

<u>Home, Family, Relationships (Figure 5)</u>

Compared to White British children, Pakistani, White Other and Black/Black British children were significantly less likely to say their family never get along well together. White Other and Other children were significantly more likely to not have a garden where they can play, compared to White British children. Compared to White British children, Pakistani children were significantly more likely, and Black/Black British children significantly less likely to have no park near home where they can play with friends. Compared to White British children, Pakistani children were significantly less likely to say they never play in a park.

<u>Material Resources (Figure 6)</u>

There were no significant ethnic differences in the proportion of children who did not have a warm winter coat. Compared to White British children, Pakistani, Indian and Gypsy/Traveller children, as well as children of Mixed ethnicity were significantly more likely to report not having three meals a day. Pakistani, White Other, Indian and Black/Black British children were significantly less likely to report having no internet at home than White British children. Compared to White British children, Pakistani, White Other, Bangladeshi and Gypsy/Irish Traveller children were significantly more likely to worry all the time about how much money their families have.

<u>Friends and School (Figure 7)</u>

For peer review only - http://bmjopen.bmj.com/site/about/guidelines.xhtml

Pakistani, White Other and Indian children were significantly less likely to report not liking school than White British children, and Bangladeshi and Black/British were significantly less likely to report not having many friends. Compared to White British children, White Other and Gypsy/Irish Traveller children were significantly less likely to say they were bullied some or all of the time, and Pakistani children and Gypsy/Irish traveller children were significantly more likely to say they were mean to others all the time. There were no significant ethnic differences in the proportion of children who felt left out by others all the time.

Subjective Wellbeing (Figure 8)

There were no significant ethnic differences in the proportion of children who said they were never happy or were always sad or who said they keep worries to themselves. Compared to White British children, only Pakistani children were significantly more likely to say they were always ill or unwell. Compared to White British children, Bangladeshi children were significantly less likely, and Gypsy/Irish traveller children more likely, to say they cannot work out what to do when things are hard.

For peer review only

Discussion

Statement of Principal Findings

Although most primary school children aged 7-10 in Bradford had good levels of wellbeing on most indicators across multiple domains, fewer than 10% had no vulnerabilities at all, and a worrying 10% had more than one vulnerability in all the four domains we studied. Variations in vulnerabilities by ethnicity were complex, with children in Black, Asian and Minority Ethnic groups sometimes reporting more vulnerabilities and sometimes fewer than White British children. We considered six vulnerabilities to be of particular concern during the current Covid-19 pandemic and associated national and local lockdowns (family never gets along well together; no garden where child can play; no nearby park where they can play; not having three meals a day; no internet at home; worries about money all the time). Before the pandemic, the majority – two-thirds – of Bradford children had one or more of these vulnerabilities. The domain with the highest proportion of children reporting vulnerabilities was Friends and School with one in 20 children reporting that they are mean to other children all the time, more than one in 10 reporting feeling left out all the time, not having many friends and not liking school, and the majority of children reporting being bullied some or all of the time.

We found that boys reported significantly more vulnerabilities for all indicators in the Home, Family and Family Relationships domain, as well as for the majority of indicators in the Material Resources, Friends and School, and Subjective Wellbeing domains. This contrasts with the finding of few consistent gender differences in wellbeing and a trend towards worse wellbeing in girls in the UK State of the Nation 2019: Children and Young People's Wellbeing report,[4] and the general picture in the research literature of worse wellbeing for adolescent girls [16 17] and inconsistent gender differences among adults.[18 19] These differences may be related to the younger age of our sample compared to many other studies which focus on secondary school pupils and adolescents. Future Born in Bradford data collection will enable analysis of whether these gender differences in wellbeing persist after children transition to secondary school. Our findings highlight a need for greater attention to boys' wellbeing among primary age children in both research and local practice. There is evidence of beneficial effects of male-targeted health promotion interventions to improve wellbeing among male adolescents,[20] however the evidence base on interventions to support wellbeing among primary school aged boys remains limited. This finding also

For peer review only - http://bmjopen.bmj.com/site/about/guidelines.xhtml

suggests that worse wellbeing among adolescent girls might not be inevitable if prevention efforts were started before secondary education.

We found complex variations in vulnerabilities by ethnicity, with children in Black, Asian and Minority Ethnic groups sometimes reporting more vulnerabilities and sometimes fewer than White British children. For example, compared to children of Pakistani heritage, White British children were more likely to say that their family never gets along well and to have no access to the internet at home, whereas children with Pakistani heritage were more likely than White British children to say they had no park near their home where they can play with friends, to report not having three meals a day and to worry all the time about how much money their families have. The UK State of the Nation 2019: Children and Young People's Wellbeing report drew attention to the importance of "*moving beyond the average*" in understanding wellbeing but reported "no discernible differences in wellbeing by…ethnicity" whilst recognising that small sample sizes might have obscured differences and reporting statistics only for five broadly defined ethnic groups.[4] We provide a detailed description of wellbeing for nine ethnic groups, so that nuances in ethnic variation can be better understood, including for smaller ethnic groups. For example, we found that Gypsy/Irish Traveller children were less likely than White British children to say they were bullied some or all of the time, but more likely to say they were mean to others, can never work out what to do when things are hard, worry all the time about how much money their family has, and not have three meals a day.

Strengths and weaknesses of the study

To our knowledge, ours is the only large contemporary study of wellbeing in primary school aged children in England, with sufficient numbers of children belonging to some of the UK's smaller Black, Asian and Minority Ethnic communities to be able to describe their wellbeing. Our approach to school recruitment, opt-out consent and whole classroom testing, along with Born in Bradford's longstanding trusting relationships with schools and communities ensured a very high response rate. Although our study is based in a single city, it is likely to be generalizable to other UK cities with high levels of ethnic diversity and deprivation.

The fact that our measures of wellbeing are self-reported by children is both a strength and a potential weakness. It has been argued that, for adults and children alike, the subjective experience of quality of life matters, and that, as feelings have a demonstrable objective

For peer review only - http://bmjopen.bmj.com/site/about/guidelines.xhtml

neuropsychological reality and are correlated with objective health, social and economic outcomes, they are worthy of measurement and inference.[21 22] Clearly, our measures of vulnerability not only include things that can only be measured subjectively, such as happiness or feeling left out, but also things that arguably would be better measured objectively, e.g., having access to a garden or three meals a day. However, it is possible that even for measures such as these, children's perceptions that they lack material resources may be important even if they actually have the resources.

Our data collection objectives and approach to categorising vulnerabilities in wellbeing was to capture a broad range of children's circumstances and experiences that might underpin variation in developing life trajectories. This is distinct from the Adverse Childhood Experiences (ACEs) approach[23] and the definition and measurement of extreme child vulnerabilities collated and reported by the Children's Commissioner for England (https://www.childrenscommissioner.gov.uk/chldrn/), both of which measure factors that put children at immediate, as well as long-term, risk of harm. Our objective was to collect data on more common wellbeing outcomes that are also risk factors with known long-term consequences for health and wellbeing, to better understand children's subjective experiences across the whole population, and to measure wellbeing itself rather than social indicators, such as determinants of wellbeing, or well-becoming.[22]In our school-based whole classroom surveys, time constraints and study design prevented data collection and analyses that would have allowed us to go beyond description and investigate the causes of vulnerabilities in child wellbeing. For BiB children only, we examined associations between neighbourhood area deprivation (Index of Multiple Deprivation) and vulnerabilities. We found no significant associations for any domain except for more vulnerabilities in Material Resources at higher levels of deprivation, and as lack of material resources is definitional for deprivation do not report those analyses here (See Supplementary Materials, Table S5). We plan future data collection with this sample to allow us to examine how vulnerabilities at primary school ages shape trajectories of wellbeing across time.

Implications for providers of services to children and policymakers

Implications for services and schools: Our findings showed the importance of friendships and school environments as key stressors for children. Schools are well placed to identify concerns and intervene early to support children's wellbeing. There is a growing evidence

For peer review only - http://bmjopen.bmj.com/site/about/guidelines.xhtml

base on ‘what works’ to support wellbeing and mental health in schools,[24] however most studies have focused on adolescent age groups.

Wellbeing is also increasingly recognised as a central aim and focus of education, for example through the Well Schools Movement (https://www.well-school.org/), and the roll-out of mental health support in schools.[25] As visible, trusted spaces in communities, schools can also be employed to engage and connect with families and enhance access to support where required.[26] Although all schools work hard to create safe and supportive environments for children, the high levels of bullying and social isolation that we report suggests that more needs to be done to make this a reality for all children, and to address inequalities in wellbeing by ethnicity. This could be facilitated through better resourcing for schools to support wellbeing and create safe and supportive environments.

From a public health perspective, multi-sectoral approaches are required to integrate access to support across services, with schools at the centre. Pathways need to facilitate identification and provision of support across services and sectors covering the domains we have identified as key concerns for children’s wellbeing – including from schools to local welfare services, voluntary sector mental health support, health care and early help. In the context of limited evidence on interventions to reduce inequalities in children’s wellbeing by gender and ethnicity, local monitoring and evaluation needs to disaggregate data to understand how services can mitigate inequalities.

Implications for national and local policymakers: Our research also highlights the importance of structural factors, from household finances and resources to green space. Action on the wider and structural determinants of wellbeing is critical to reduce inequalities in children’s wellbeing, including the wide-ranging inequalities by ethnicity. Bradford is the 13th most deprived local authority in England (Indices of deprivation 2019), and many of the schools studied fall within the most deprived neighbourhoods in England. National policies to prevent children growing up in poverty and deprived neighbourhoods include uplifts to child benefit and abolishment of the two-child limit, improved access to free school meals and improvements to the National Living Wage.[27] There is a need to direct further resources to schools, especially in deprived areas, to facilitate work to support wellbeing and reduce inequalities. At a local level, local authorities, schools and other

For peer review only - http://bmjopen.bmj.com/site/about/guidelines.xhtml

services can take action to ensure families can access advice services, limit the costs of the school day and school holidays, and facilitate access to high quality green space.

Implications in the context of the Covid-19 pandemic: Our findings suggest that, prior to the Covid-19 pandemic, a substantial number of children were experiencing sub-optimal wellbeing with potential lifelong consequences, with implications for all providers of services to children, as well as local and national policymakers. As the economic impact of the pandemic is increasing child poverty, income insecurity, food and housing insecurity, parental mental health challenges and family violence, the need for attention to the identification and amelioration of vulnerability is heightened.[28] The International Society for Social Pediatrics and Child Health has called for the adoption of "a Child Rights Based Approach (CRBA) to respond to the Covid-19 pandemic, and to advance a future in which the health, development and well-being of children and youth are prioritised with explicit strategies to reduce health inequities and advance social justice" (https://www.issop.org/2020/06/01/issop-covid-19-declaration/). Such an approach can underpin and inform services and policy making at all levels.

As the population was encouraged to 'lockdown' at home and schools closed for almost all children (and attendance was very low for those who were eligible to go to school) for varying periods, the response to the pandemic will have affected children's wellbeing (as well as their educational trajectories[29]) differently, depending on their circumstances and context. Children who found school a nurturing refuge from difficult home situations will have suffered from school shutdowns, whereas others who found school a challenging environment may have been happy to be at home for a prolonged period; most children have probably experienced both positive and negative impacts.[30 31] In addition, our subsequent research has confirmed that the high prevalence of children with no access to the internet at home and no access to a garden or park has meant that many children have at times been unable to study[30] or be physically active.[32]

Locally, our research team has engaged with our local authority, public health, NHS and voluntary sector colleagues to identify principles to mitigate the impact of the pandemic on vulnerable groups (https://www.bradfordresearch.nhs.uk/wp-content/uploads/2020/04/CSAG-Briefing-Paper-Vulnerable-Groups-and-Recovery-

For peer review only - http://bmjopen.bmj.com/site/about/guidelines.xhtml

230420.pdf). We have proposed additional support for children and young people in post-pandemic recovery planning, including assessing the impact of decision making on children, prioritising children's needs, providing extra support for vulnerable children, and monitoring impacts on children's lives and wellbeing.

Conclusions and implications for public health

Among primary school children aged 7-10 in a deprived Northern city in the UK, fewer than 10% had no vulnerabilities in Home, Family and Family Relationships, Material Resources, Friends and School and Subjective Wellbeing, while 10% had more than one vulnerability in all of these domains. These children are at risk of poor health, social and economic trajectories throughout their lives. For public health, our findings highlight the emergence of inequalities in child wellbeing at an early age, and the need for early intervention to improve children's lives and reduce impacts later in life. Multi-sectoral approaches are required to address the wide range of domains, across health, schools and wider local services, with integrated pathways to identify and support children. Inequalities in child wellbeing could be mitigated by national policy and local practice focused at structural factors, such as poverty eradication, and school-based approaches to identify and support wellbeing using both universal approaches and targeted support for the most vulnerable.

For peer review only - http://bmjopen.bmj.com/site/about/guidelines.xhtml

References

1. UNICEF Innocenti Research Centre. Child poverty in perspective: An overview of child well-being in rich countries. Florence: Innocenti Report Card, 2007

2. Layard R, Dunn J. A Good Childhood: Searching for values in a competitive age. London: Penguin 2009.

3. The Children's Society. The Good Childhood Report. London: The Children's Society, 2012-2019

4. Department for Education. State of the Nation 2019: Children and Young People's Wellbeing. London: Department for Education, UK Government, 2019

5. International Society for Social Pediatrics and Child Health. Declaration: Advancing Health Equity and Social Justice in Response to COVID-19: https://www.issop.org/cmdownloads/issop_covid-declaration/, 2020

6. Office for National Statistics. 2011 Census: Key Statistics and Quick Statistics for Local Authorities in the United Kingdom. 2013, 2013

7. Public Health England. Public Health Outcomes Framework: Yorkshire and Humber 2020 [Available from: https://tinyurl.com/y2y8yo84 accessed 25 July 2020.

8. Bradford Institute for Health Research. Overview: BIHR Covid-19 Scientific Advisory Group 2020 [Available from: https://www.bradfordresearch.nhs.uk/wp-content/uploads/2020/05/C-SAG-Overview-210520.pdf accessed 25 July 2020.

9. Raynor P, Born in Bradford Collaborative G. Born in Bradford, a cohort study of babies born in Bradford, and their parents: Protocol for the recruitment phase. *BMC public health* 2008;8(1):327.

10. Wright J, Small N, Raynor P, et al. Cohort profile: The Born in Bradford multi-ethnic family cohort study. *International journal of epidemiology* 2012 doi: 10.1093/ije/dys112 [published Online First: 2012/10/16]

For peer review only - http://bmjopen.bmj.com/site/about/guidelines.xhtml

11. Bird PK, McEachan RRC, Mon-Williams M, et al. Growing up in Bradford: protocol for the age 7-11 follow up of the Born in Bradford birth cohort. *BMC public health* 2019;19(1):939. doi: 10.1186/s12889-019-7222-2 [published Online First: 2019/07/14]

12. Shire KA, Mon-Williams M, Hill LJB, et al. The Born in Bradford Primary School Years project: In school assessment of 7-11 year old's cognitive and sensorimotor abilities. *Wellcome Open Research* In preparation

13. National Centre for Social Research. Child of the new century: age 7 survey: http://doc.ukdataservice.ac.uk/doc/6411/mrdoc/pdf/mcs4_child_self_completion.pdf, 2008

14. International Survey of Children's Wellbeing. Children's Worlds: ISCWeB: https://isciweb.org/wp-content/uploads/2019/12/Childrens-Worlds_Q08_100713_Website.pdf, 2017

15. Avon Longitudinal Study of Parents and Children. Child completed questionnaires: http://www.bristol.ac.uk/alspac/researchers/our-data/questionnaires/child-completed-questionnaires/, Accessed: 30/12/2020

16. Public Health England. Wellbeing of adolescent girls: An analysis of data from the Health Behaviour in School-aged Children (HBSC) survey for England, 2014 London: Public Health England, 2017

17. Deighton J, Lereya T, Patalay P, et al. Mental health problems in young people aged 11-14: Results from the first HeadStart annual survey of 30,000 children. London: CAMHS Press 2018.

18. Batz C, & Tay, L. . Gender differences in subjective well-being. In: E. Diener SO, & L. Tay, ed. Handbook of well-being. Salt Lake City, UT: DEF Publishers 2018.

19. Batz-Barbarich C, Tay L, Kuykendall L, et al. A Meta-Analysis of Gender Differences in Subjective Well-Being: Estimating Effect Sizes and Associations With Gender Inequality. *Psychological Science* 2018;29(9):1491-503. doi: 10.1177/0956797618774796

20. Gwyther K, Swann R, Casey K, et al. Developing young men's wellbeing through community and school-based programs: A systematic review. *PloS one* 2019;14(5):e0216955. doi: 10.1371/journal.pone.0216955

For peer review only - http://bmjopen.bmj.com/site/about/guidelines.xhtml

21. Bradshaw J. The well-being of children in the UK. Bristol: Policy Press 2016.

22. Ben-Arieh A. Developing indicators for child well-being in a changing context. In: McAuley R, Rose W, eds. Child well-being: Understanding children's lives. London: Jessica Kingley Publishers 2010:129-42.

23. Felitti VJ, Anda RF, Nordenberg D, et al. Relationship of childhood abuse and household dysfunction to many of the leading causes of death in adults. The Adverse Childhood Experiences (ACE) Study. *Am J Prev Med* 1998;14(4):245-58. doi: 10.1016/s0749-3797(98)00017-8 [published Online First: 1998/06/23]

24. Clarke A, Sorgenfrei M, Mulcahy J, et al. Adolescent mental health: A systematic review on the effectiveness of school-based interventions: Early Intervention Foundation, 2021.https://www.eif.org.uk/report/adolescent-mental-health-a-systematic-review-on-the-effectiveness-of-school-based-interventions

25. Department for Education. Promoting and supporting mental health and wellbeing in schools and colleges: UK Government, 2021.https://www.gov.uk/guidance/mental-health-and-wellbeing-support-in-schools-and-colleges

26. Mon-Williams M, Ayadurai C, Bond C, et al. Schools and education. In: Pickett K. T-RD, et al, ed. The Child of the North: Building a fairer future after COVID-19: Northern Health Science Alliance and N8 Research Partnership 2021. https://www.thenhsa.co.uk/app/uploads/2021/12/Child-of-the-North-Report-FINAL.pdf

27. Bradshaw J, Wickham S, Alexiou A, et al. Child poverty, inequality and deprivation. In: Pickett K. T-RD, et al, ed. The Child of the North: Building a fairer future after COVID-19: Northern Health Science Alliance and N8 Research Partnership 2021. https://www.thenhsa.co.uk/app/uploads/2021/12/Child-of-the-North-Report-FINAL.pdf

28. Dickerson J KB, Lockyer B, Bridges S, Cartwright C, Willan K, Shire, K, Crossley K, Sheldon TA, Lawlor DA, Wright J, McEachan RRC, Pickett KE. Experiences of lockdown during the Covid-19 pandemic: descriptive findings from a survey of families in the Born in Bradford study [version 2; peer review: 2 approved]. *Wellcome Open Research* 2020;5(228):(https://doi.org/10.12688/wellcomeopenres.6317.1).

For peer review only - http://bmjopen.bmj.com/site/about/guidelines.xhtml

29. Waterman A, Shire K. The impact of Covid-19 on learning and wellbeing. Bradford: Centre for Applied Educational Research, 2020.https://caer.org.uk/wp-content/uploads/2020/11/CAERschoolsurveyCovid.pdf

30. Dickerson J, Hou B, Lockyer B, et al. 'I think I am falling behind': Findings of the Born in Bradford Covid19 Children Survey. Bradford: Bradford Institute for Health Research, 2020.https://www.bradfordresearch.nhs.uk/wp-content/uploads/2020/11/BiB-Covid-19-Child-Briefing-Summary-V1.0-August-2020.pdf

31. Moss R EC, Lockyer B, Iqbal H, Kelly B, McIvor C, Crossley K, Sheard L, Islam S, Razaq R, Zarate M, Anser Z, Bridges S, Smith H, Lawlor D, Willan K, Rahman A, McEachan R, Wright J, Bryant M, Pickett K, Dickerson J. "Falling behind at school": Summary of the free-text findings from Phase 2 (Oct – Dec 2020) of the Born in Bradford Covid-19 Child Survey. Bradford: Bradford Institute for Health Research, 2021.https://www.bradfordresearch.nhs.uk/wp-content/uploads/2021/10/Ph-2-Child-Qual-v0.8-FINAL.pdf

32. Bingham DD, Daly-Smith A, Hall J, et al. Covid-19 lockdown: Ethnic differences in children's self-reported physical activity and the importance of leaving the home environment; a longitudinal and cross-sectional study from the Born in Bradford birth cohort study. *International Journal of Behavioral Nutrition and Physical Activity* 2021;18(1):117. doi: 10.1186/s12966-021-01183-y

For peer review only

For peer review only - http://bmjopen.bmj.com/site/about/guidelines.xhtml

Ethics approval

Ethical approval has been obtained from the National Health Service Health Research Authority Yorkshire and the Humber (Bradford Leeds) Research Ethics Committee (reference: 16/YH/0062).

Transparency statement

Kate Pickett (the manuscript's guarantor) affirms that the manuscript is an honest, accurate, and transparent account of the study being reported; that no important aspects of the study have been omitted; and that any discrepancies from the study as originally planned have been explained.

Competing interests

No authors declare competing interests.

Contributions

KEP, DAL, JWr, RM, MM-W, NS obtained funding for the study, and with PKB and JD conceived the research questions and analyses. BK, BH, MA, KS cleaned and curated the data, conducted analyses and contributed to the methodology. KEP drafted the first manuscript; KEP and PKB revised the manuscript. All authors read and approved the final version of the manuscript.

Data Availability

Underlying data

Scientists are encouraged and able to use BiB data, which are available through a system of managed open access. The steps below describe how to apply for access to BiB data.

Before you contact BiB, please make sure you have read our Guidance for Collaborators. Our BiB Executive review proposals on a monthly basis and we will endeavour to respond to your request as soon as possible. You can find out about the different datasets which are available here. If you are unsure if we have the data that you need please contact a member of the BiB team (borninbradford@bthft.nhs.uk).

Once you have formulated your request please complete the 'Expression of Interest' form available here and send to the BiB Programme Director (rosie.mceachan@bthft.nhs.uk).

For peer review only - http://bmjopen.bmj.com/site/about/guidelines.xhtml

If your request is approved, we will ask you to sign a collaboration agreement and, if your request involves biological samples, we will ask you to complete a material transfer agreement.

Extended data

This project contains the following extended data:

- Supplementary Materials

Data are available under the terms of the Creative Commons Zero "No rights reserved" data waiver (CC0 1.0 Public domain dedication).

Acknowledgements

This study was funded by a joint grant from the UK Medical Research Council and UK Economic and Social Science Research Council (MR/N024397/1), with support from a Wellcome Trust infrastructure grant (WT101597MA) and the National Institute for Health Research under its Applied Research Collaboration Yorkshire and Humber (NIHR200166). The funders had no role in the design of the study, the collection, analysis, or interpretation of the data; the writing of the manuscript, or the decision to submit the manuscript for publication. All authors are independent of the funders, had full access to all of the data (including statistical reports and tables) in the study and can take responsibility for the integrity of the data and the accuracy of the data analysis. Views expressed in this paper are those of the authors and not necessarily those of any funder.

Born in Bradford is only possible because of the enthusiasm and commitment of the Children and Parents in BiB. We are grateful to all the participants, health professionals, schools, and researchers who have made Born in Bradford happen. Research results are disseminated through https://borninbradford.nhs.uk/our-findings/ and actively through various engagement activities to parents, children and young people and stakeholders.

For peer review only - http://bmjopen.bmj.com/site/about/guidelines.xhtml

Patient and Participant Involvement

BiB is committed to the co-production of research. Our child wellbeing survey was shared with the Parent Governors group for feedback during development, and piloted with Bradford school children.

Summary

What is already known on this topic

- Child wellbeing in the UK is low in comparison to other rich countries
- Little is known about the wellbeing of children under the age of 10 in the UK
- Little is known about ethnic variations in child wellbeing in the UK

What this study adds

- Although most 7-10 year old children in our study had good levels of wellbeing, fewer than 10% had no vulnerabilities at all, a worrying 10% had at least one vulnerability in all the domains we studied, and two thirds had vulnerabilities of concern during the Covid-19 lockdowns
- In contrast to studies of older children, boys had more wellbeing vulnerabilities, although girls were more likely to have no friends, report being bullied and feeling left out.
- Ethnic variations in vulnerabilities in wellbeing were complex, with children from ethnic minorities reporting fewer of some vulnerabilities than White British children and more of others; our study is the first to report a wide range of vulnerabilities across a large number of ethnic groups in this age range

For peer review only - http://bmjopen.bmj.com/site/about/guidelines.xhtml

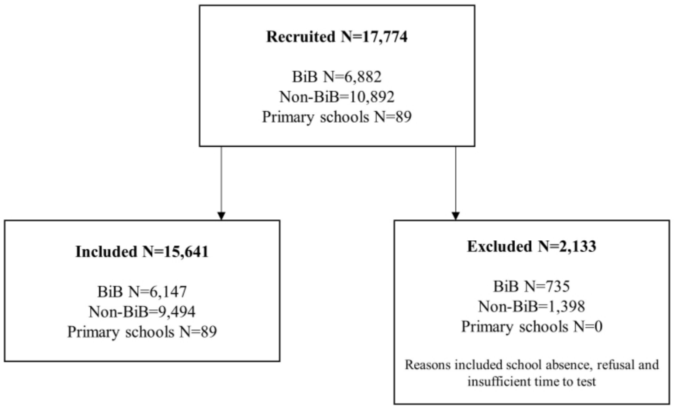

Figure 1: Recruitment of schools, Born in Bradford cohort members and non-Born in Bradford children to the BiB Primary School Years wellbeing survey

254x190mm (300 x 300 DPI)

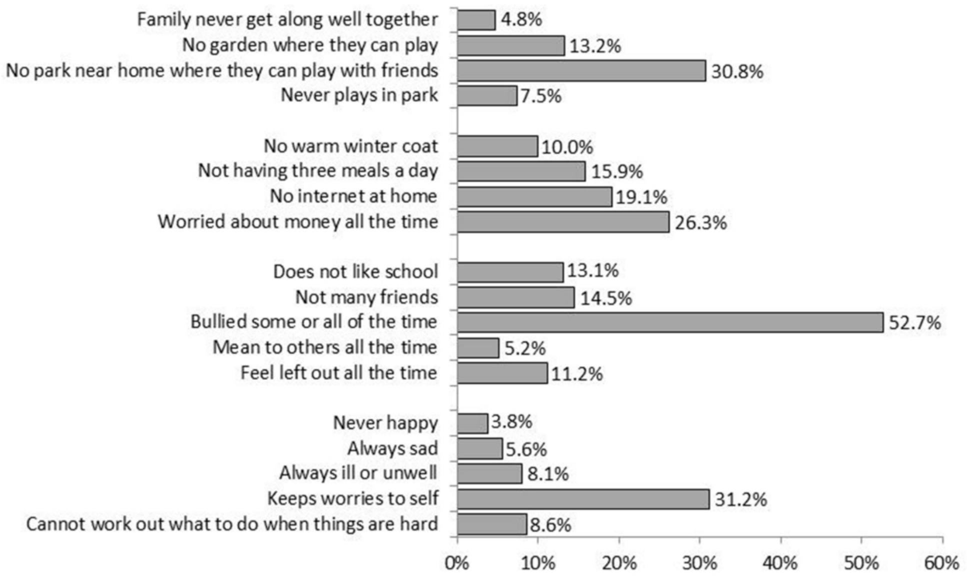

Figure 2: Percent of primary school children with each vulnerability across domains of wellbeing

254x190mm (300 x 300 DPI)

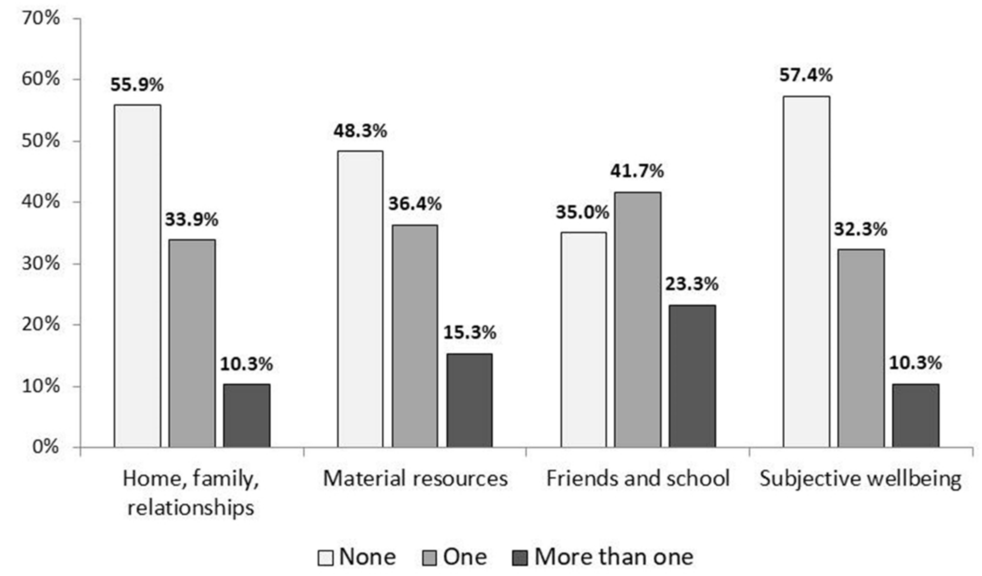

Figure 3: Percent of primary school children with zero, one or more than vulnerability in each domain of wellbeing

254x190mm (300 x 300 DPI)

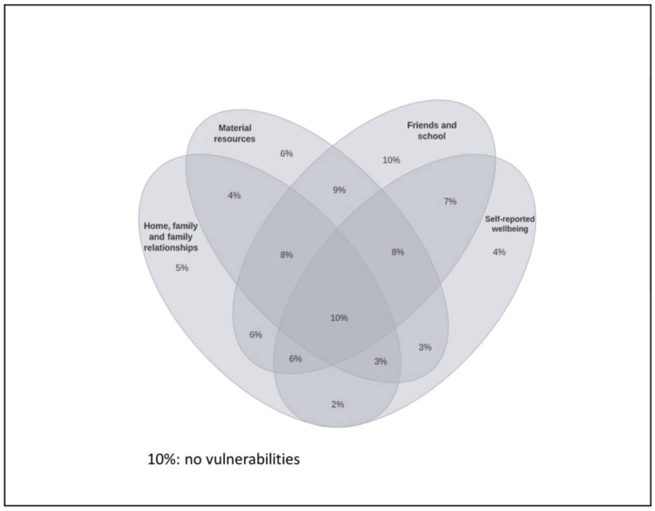

Figure 4: Percent of children with vulnerabilities within and across domains

254x190mm (300 x 300 DPI)

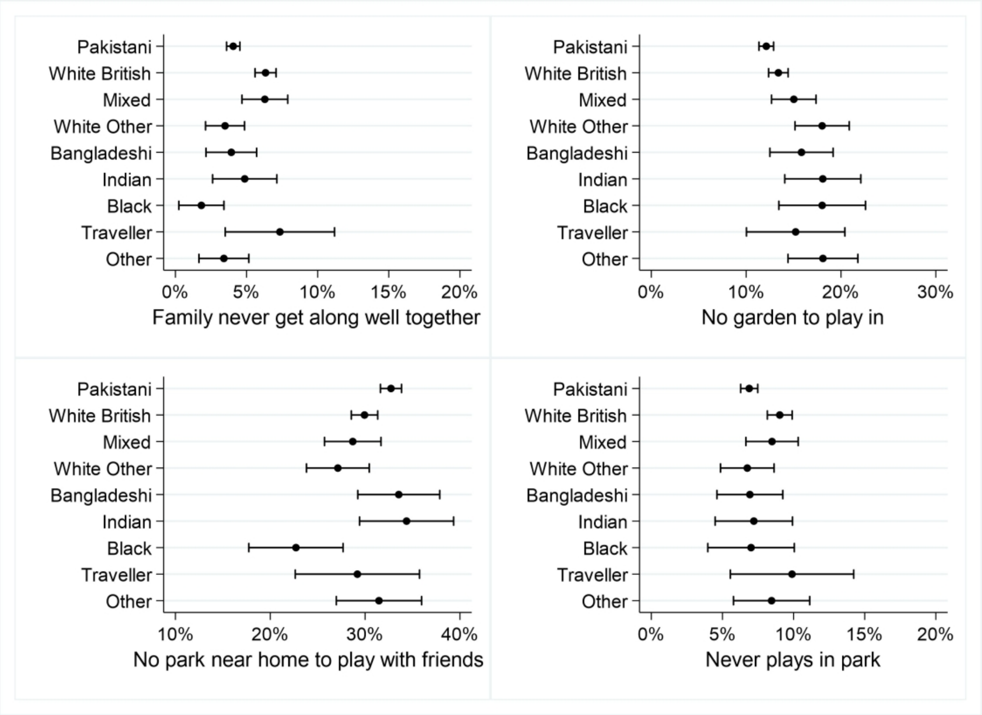

Figure 5: Percent of primary school children with at least one vulnerability in the Home, Family and Family Relationships domain

254x190mm (300 x 300 DPI)

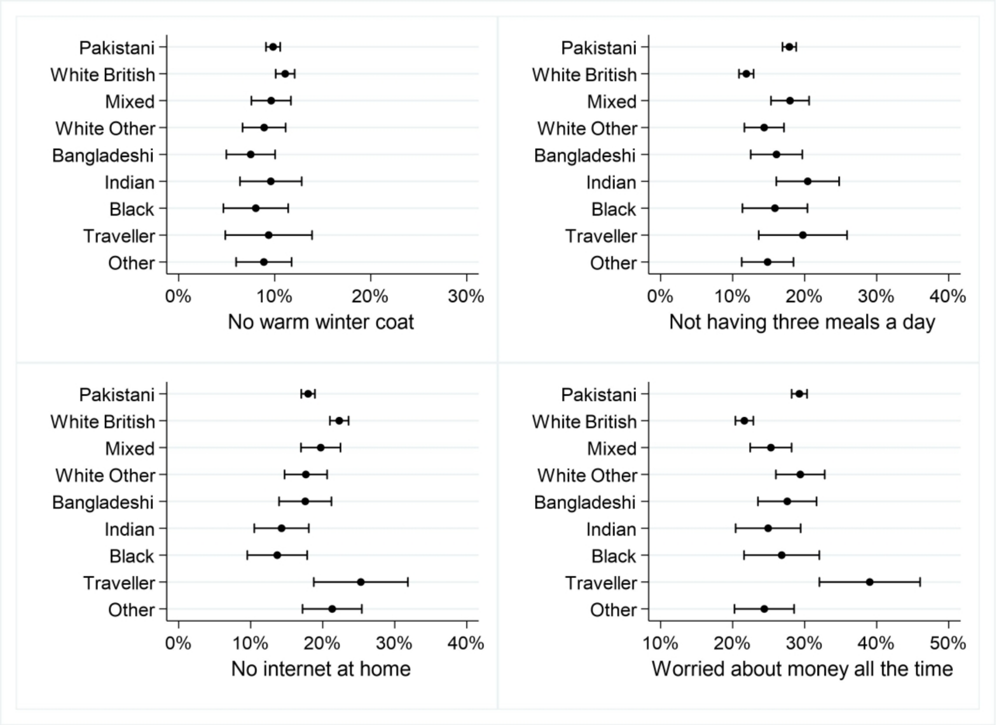

Figure 6: Percent of primary school children with at least one vulnerability in the Material Resources domain

254x190mm (300 x 300 DPI)

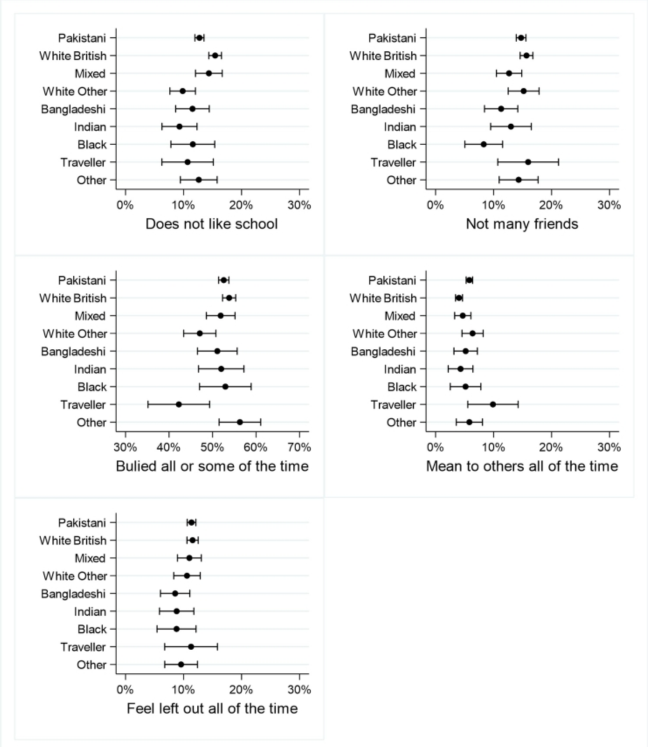

Figure 7: Percent of primary school children with at least one vulnerability in the Friends and School domain

254x190mm (300 x 300 DPI)

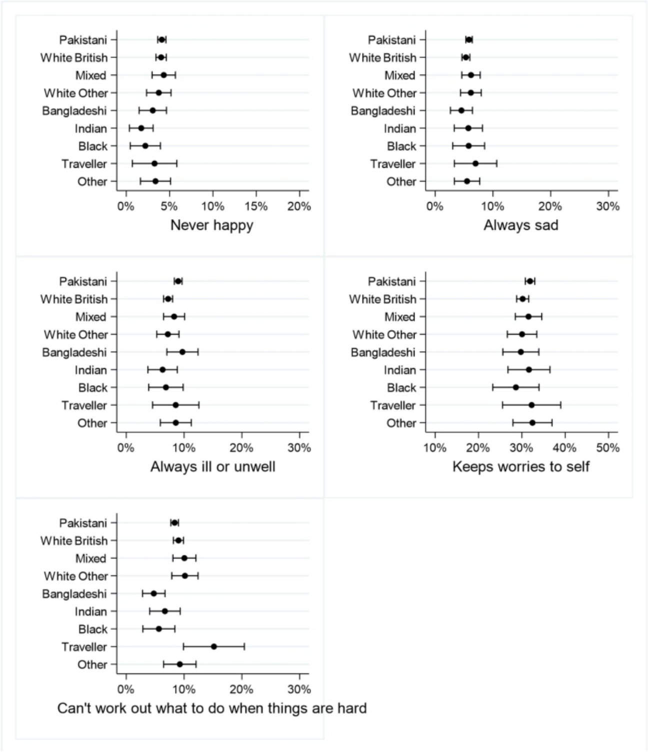

Figure 8: Percent of primary school children with at least one vulnerability in the Subjective Wellbeing domain

254x190mm (300 x 300 DPI)

Supplementary Materials: Vulnerabilities in child wellbeing among primary school children: a cross sectional study in Bradford, UK

1: STROBE Statement—Checklist of items that should be included in reports of ***cross-sectional studies***

	Item No	Recommendation
**Title and abstract**	1	(*a*) Indicate the study's design with a commonly used term in the title or the abstract SEE TITLE
		(*b*) Provide in the abstract an informative and balanced summary of what was done and what was found SEE ABSTRACT
**Introduction**		
Background/rationale	2	Explain the scientific background and rationale for the investigation being reported<br>SEE INTRODUCTION, PAGE 4
Objectives	3	State specific objectives, including any prespecified hypotheses<br>N/A
**Methods**		
Study design	4	Present key elements of study design early in the paper<br>SEE METHODS, PAGES -7
Setting	5	Describe the setting, locations, and relevant dates, including periods of recruitment, exposure, follow-up, and data collection<br>SEE SETTINGS AND PARTICIPANTS, PAGE 5
Participants	6	(*a*) Give the eligibility criteria, and the sources and methods of selection of participants<br>SEE SETTINGS AND PARTICIPANTS, PAGE 5
Variables	7	Clearly define all outcomes, exposures, predictors, potential confounders, and effect modifiers. Give diagnostic criteria, if applicable<br>SEE DESIGN AND PROCEDURES, PAGE 6
Data sources/ measurement	8*	For each variable of interest, give sources of data and details of methods of assessment (measurement). Describe comparability of assessment methods if there is more than one group<br>SEE DESIGN AND PROCEDURES, PAGE 6
Bias	9	Describe any efforts to address potential sources of bias<br>SEE FIGURE 1, PAGE 5
Study size	10	Explain how the study size was arrived at<br>SEE SETTINGS AND PARTICIPANTS, PAGE 5
Quantitative variables	11	Explain how quantitative variables were handled in the analyses. If applicable, describe which groupings were chosen and why<br>SEE DESIGN AND PROCEDURES, PAGE 6
Statistical methods	12	(*a*) Describe all statistical methods, including those used to

For peer review only

		control for confounding
		(*b*) Describe any methods used to examine subgroups and interactions
		(*c*) Explain how missing data were addressed
		(*d*) If applicable, describe analytical methods taking account of sampling strategy
		(*e*) Describe any sensitivity analyses<br>SEE DATA ANALYSIS, PAGE 7
**Results**		
Participants	13*	(a) Report numbers of individuals at each stage of study—eg numbers potentially eligible, examined for eligibility, confirmed eligible, included in the study, completing follow-up, and analysed
		(b) Give reasons for non-participation at each stage
		(c) Consider use of a flow diagram<br>SEE METHODS, FIGURE 1, PAGE 5 AND ALL RESULTS TABLES
Descriptive data	14*	(a) Give characteristics of study participants (eg demographic, clinical, social) and information on exposures and potential confounders<br>SEE TABLE 1, PAGE 8
		(b) Indicate number of participants with missing data for each variable of interest<br>SEE ALL TABLES AND FIGURES
Outcome data	15*	Report numbers of outcome events or summary measures<br>SEE FIGURE 1, PAGE 9
Main results	16	(*a*) Give unadjusted estimates and, if applicable, confounder-adjusted estimates and their precision (eg, 95% confidence interval). Make clear which confounders were adjusted for and why they were included<br>SEE ALL TABLES AND FIGURES
		(*b*) Report category boundaries when continuous variables were categorized<br>N/A
		(*c*) If relevant, consider translating estimates of relative risk into absolute risk for a meaningful time period<br>N/A
Other analyses	17	Report other analyses done—eg analyses of subgroups and interactions, and sensitivity analyses<br>SEE DISCUSSION RE: UNREPORTED ANALYSIS OF IMD, PAGE 18
**Discussion**		
Key results	18	Summarise key results with reference to study objectives<br>SEE STATEMENT OF PRINCIPAL FINDINGS, PAGE 16
Limitations	19	Discuss limitations of the study, taking into account sources of potential bias or imprecision. Discuss both direction and magnitude of any potential bias

For peer review only

		SEE STRENGTHS AND WEAKNESSES OF THE STUDY, PAGE 17
Interpretation	20	Give a cautious overall interpretation of results considering objectives, limitations, multiplicity of analyses, results from similar studies, and other relevant evidence<br>SEE IMPLICATIONS SECTION, PAGE 18
Generalisability	21	Discuss the generalisability (external validity) of the study results<br>SEE STRENGTHS AND WEAKNESSES OF THE STUDY, PAGE 17
**Other information**		
Funding	22	Give the source of funding and the role of the funders for the present study and, if applicable, for the original study on which the present article is based<br>SEE FUNDING STATEMENT

For peer review only

2. Me and My Life wellbeing survey

Me and my life

Please tick the box next to your answer

Please try to answer all the questions. There are no right or wrong answers. We want to know what you think.

Your answers will not be shown to anyone that you know (including mums and dads).

If you do not want to answer a question you can miss it out.

If you need any help, you can ask your teacher or the person who gave you this quiz.

Thank you!

For peer review only

1. Which of these would you most like to eat today?

Pizza 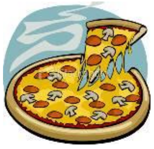 ☐

Curry 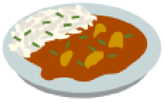 ☐

Pasta with sauce 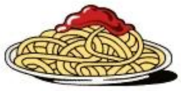 ☐

Sandwich 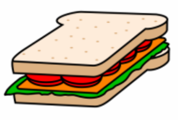 ☐

Where I live

2. How much do you like the home where you live?

I like it a lot 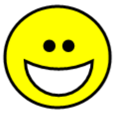 ☐

I like it a bit 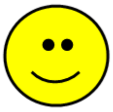 ☐

I don't like it 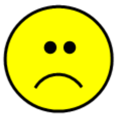 ☐

For peer review only

3. How often do you play in a park?

Very often ☐

Sometimes ☐

Never ☐

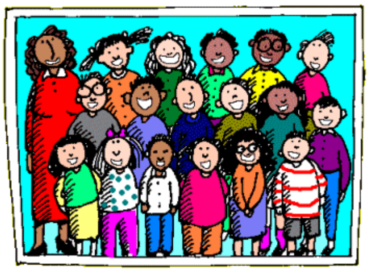

My family and the people I live with

4. How often do you have fun with your family at the weekend?

All of the time ☐

Some of the time ☐

Never ☐

5. How often does your family get along well together?

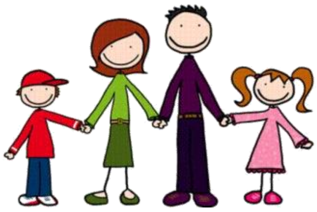

All of the time ☐

Some of the time ☐

Never ☐

6. How often do you get along with your brothers, sisters and other children you live with?

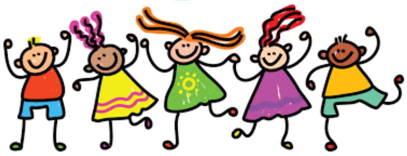

All of the time ☐

Some of the time ☐

Never ☐

I don't have brothers or sisters or live with other children ☐

For peer review only

7. What languages do you speak with your family at home?

You can tick **more than one** answer if you like.

- English ☐
- Urdu ☐
- Mirpuri/Punjabi ☐
- Slovakian ☐
- Arabic ☐
- Bangla/Bengali ☐
- Hindko ☐
- Pushto ☐
- Other ☐

For peer review only

A bit about me

8. Here is a list of things that some children have.

Do you have each of these things?

		Yes I have it	No I don't have it
Some money to spend on myself	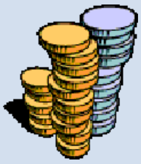	☐	☐
Clothes that you think your friends like	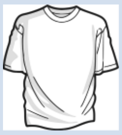	☐	☐
A warm winter coat	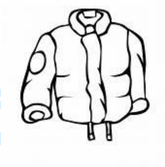	☐	☐
Your own mobile phone	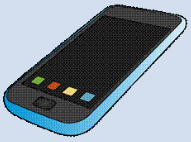	☐	☐
A computer, laptop or tablet with internet at home	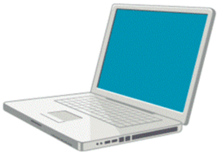	☐	☐
Three meals every day	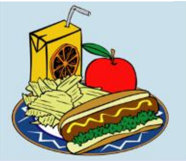	☐	☐

9. Do you have each of these things?

		Yes<br>I have it	No<br>I don't have it
**Family trips or days out at least once a month, such as to the seaside or a theme park**	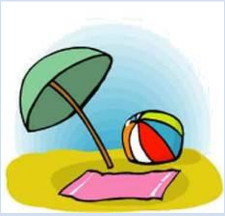	☐	☐
**At least one family holiday away from home each year**	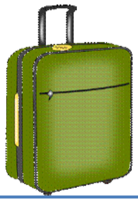	☐	☐

10. Does your home have a garden where you can play?

Yes ☐

No ☐

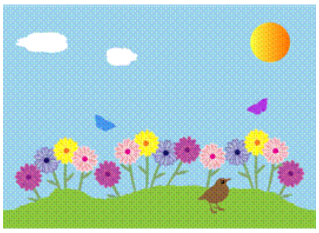

11. Does your family have a car?

Yes ☐

No ☐

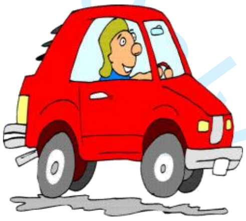

12. Do you have a park near your home where you can play with your friends?

Yes ☐

No ☐

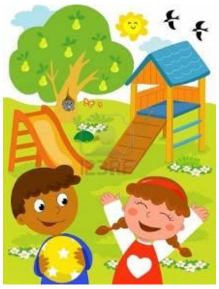

For peer review only

13. How often do you worry about how much money your family has?

All of the time ☐

Some of the time ☐

Never ☐

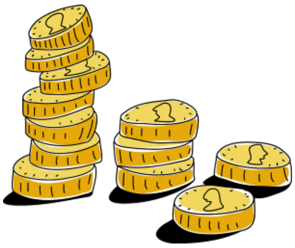

14. How often do you feel happy?

All of the time ☐

Some of the time ☐

Never ☐

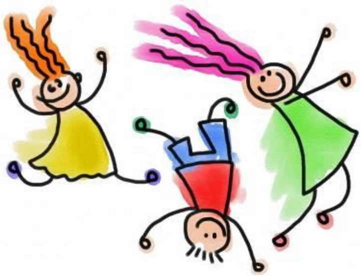

15. How often do you feel sad?

All of the time ☐

Some of the time ☐

Never ☐

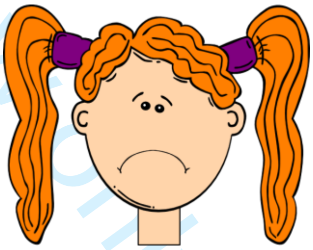

16. How often are you ill or unwell?

All of the time ☐

Some of the time ☐

Never ☐

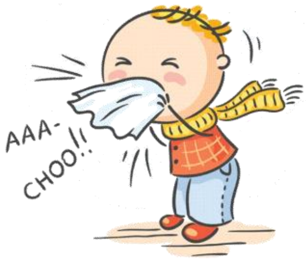

For peer review only

17. How often do you feel healthy?

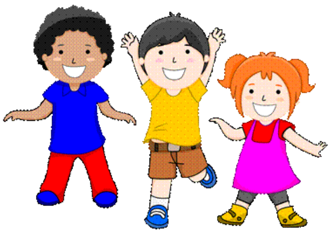

All of the time ☐

Some of the time ☐

Never ☐

18. What do you do if you are worried about something?

You can tick **<u>more than one</u>** answer if you like.

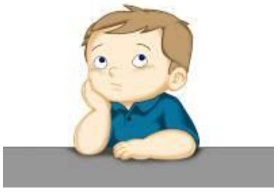

I keep it to myself ☐

I tell a friend ☐

I tell my Mum, Dad or the person who looks after me ☐

I tell a teacher ☐

19. When I find something really hard I can work out what to do next.

All of the time ☐

Some of the time ☐

Never ☐

For peer review only

My friends and other children

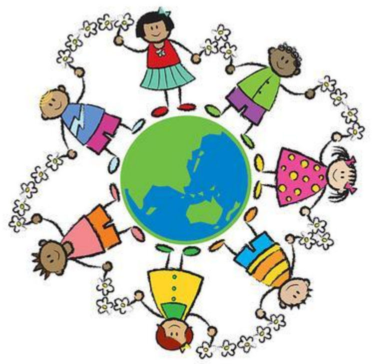

20. How many friends do you have?

Lots ☐

Some ☐

Not many ☐

21. Do you have any best friends?

Yes ☐

No ☐

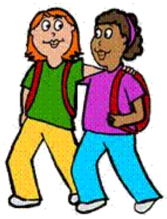

22. How much do you like playing with your friends?

I like it a lot 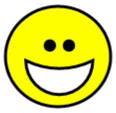 ☐

I like it a bit 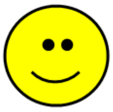 ☐

I don't like it 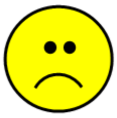 ☐

For peer review only

23. How often do other children bully you?

All of the time ☐

Some of the time ☐

Never ☐

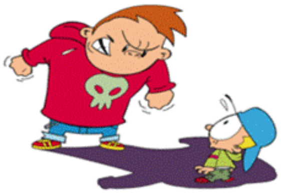

For peer review only

24. How often are you mean to other children at school?

All of the time ☐

Some of the time ☐

Never ☐

25. How often do you feel left out of things by other children?

All of the time ☐

Some of the time ☐

Never ☐

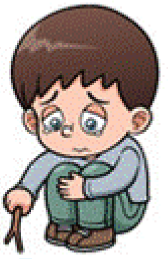

My school

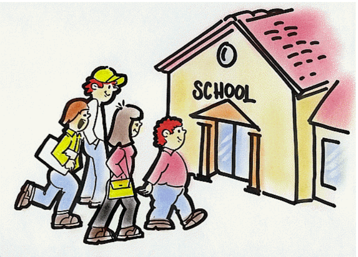

26. How much do you like school?

I like it a lot	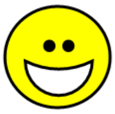	☐
I like it a bit	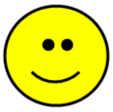	☐
I don't like it	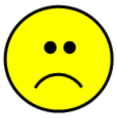	☐

After school and weekend activities

27. Do you go to any of these clubs or activities after school or at weekends?

		Yes	No
**Sports** (such as football, cricket, swimming, karate)	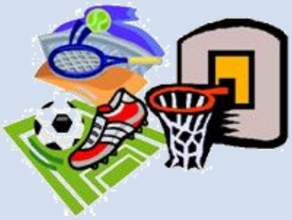	☐	☐
**Music lessons** (such as choir, piano lessons)	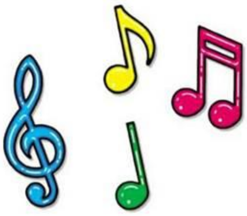	☐	☐
**Dance lessons**	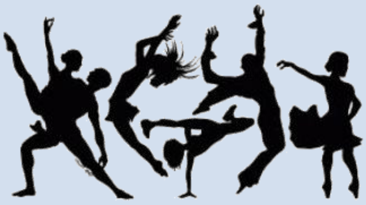	☐	☐
**Any other club** (such as art, drama, film, cooking)		☐	☐

For peer review only

Religion

28. **What is your religion?**

Christian ☐

Muslim ☐

Hindu ☐

Sikh ☐

Other religion ☐

None/No religion ☐

Don't know ☐

If you are Muslim:

Do you go to Mosque or Madrassa?

Yes ☐

No ☐

If yes: How often do you go to Mosque or Madrassa?

Most days a week ☐

Some days a week ☐

Less than once a week ☐

For peer review only

If you are Christian:

Do you go to Church or Sunday school?

Yes ☐

No ☐

If yes: How often do you go to Church or Sunday school?

Most days a week ☐

Some days a week ☐

Less than once a week ☐

If you are Hindu/Sikh:

Do you go to a Temple?

Yes ☐

No ☐

If yes: How often do you go to a Temple?

Most days a week ☐

Some days a week ☐

Less than once a week ☐

For peer review only

If you are another religion:

Do you go to a place of worship?

Yes ☐

No ☐

If yes: How often do you go to a place of worship?

Most days a week ☐

Some days a week ☐

Less than once a week ☐

For peer review only

When I grow up

29. When you grow up, would you like to be:

		Yes, I'd like it a lot	Yes, I'd like it a bit	No, not at all
A cook	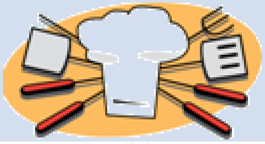	☐	☐	☐
A sports star	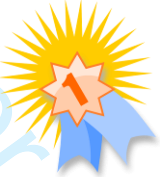	☐	☐	☐
A train driver	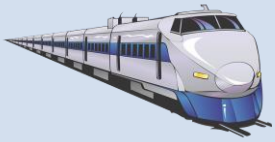	☐	☐	☐
A pop star	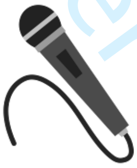	☐	☐	☐
Someone who looks after children at home	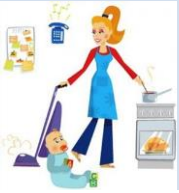	☐	☐	☐
A doctor or a nurse	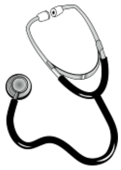	☐	☐	☐
Someone who works in a shop	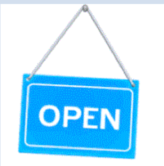	☐	☐	☐
A teacher	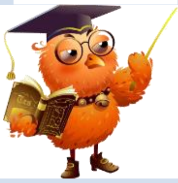	☐	☐	☐
Someone who works with computers	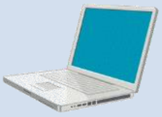	☐	☐	☐

30. What would you most like to be when you grow up?
You can write any job you like!

……

……

31. How much do you like:

		I like it a lot	I like it a bit	I don't like it/ I don't do it
Eating Asian food, such as curry	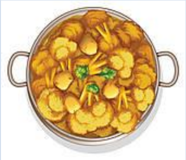	☐	☐	☐
Eating English food, such as fish and chips	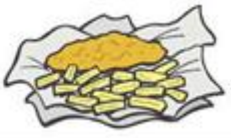	☐	☐	☐
Wearing Asian clothes such as Salwar kameez or kurta	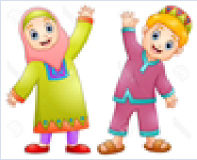	☐	☐	☐
Wearing clothes such as jeans, t-shirts or dresses	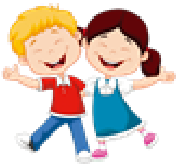	☐	☐	☐

For peer review only

32.

		Yes	No
Do you and your family celebrate Eid?	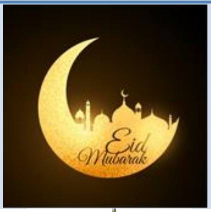	☐	☐
Do you and your family celebrate Christmas?	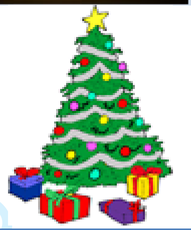	☐	☐

Thank you for your time!

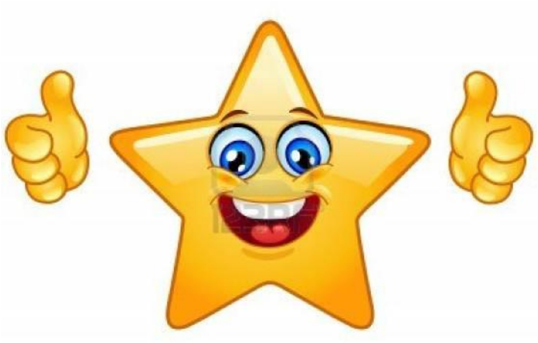

Researcher/Teacher only:
Tick if the child was assisted to complete the questionnaire. ☐

Table S1: Vulnerability across domains of wellbeing

Table S1a: Response to items within 'Home, Family and Family Relationships' domain

How often does your family get along well	Number	Percentage (95% CI)
Never	729	4.8% (4.4%-5.1%)
Some of the time	6,845	44.8% (44.1%-45.6%)
All of the time	7,689	50.4% (49.6%-51.2%)
Missing	378	
Total	15,641	100.0%

Does your home have a garden where you can play?	Number	Percentage (95% CI)
No	2,036	13.2% (12.7%-13.8%)
Yes	13,357	86.8% (86.2%-87.3%)
Missing	248	
Total	15,641	100.0%

Do you have a park near your home where you can play with your friends?	Number	Percentage (95% CI)
No	4,711	30.8% (30.1%-31.5%)
Yes	10,584	69.2% (68.5%-69.9%)
Missing	346	
Total	15,641	100.0%

How often do you play in a park?	Number	Percentage (95% CI)
Never	1,137	7.5% (7.1%-7.9%)
Sometimes	11,701	76.9% (76.2%-77.5%)
Very often	2,386	15.7% (15.1%-16.3%)
Missing	417	
Total	15,641	100.0%

For peer review only

Supplementary Table S1b: Response to items within 'Material Resources' domain

Do you have a warm winter coat?	Number	Percentage (95% CI)
No	1,402	10% (9.5%-10.5%)
Yes	12,594	90% (89.5%-90.5%)
Missing	1,645	
Total	15,641	100.0%

Do you have three meals every day?	Number	Percentage (95% CI)
No	2,243	15.9% (15.3%-16.5%)
Yes	11,891	84.1% (83.5%-84.7%)
Missing	1,507	
Total	15,641	100.0%

Do you have a computer/laptop/tablet with internet at home?	Number	Percentage (95% CI)
No	2,733	19.1% (18.5%-19.8%)
Yes	11,563	80.9% (80.2%-81.5%)
Missing	1,345	
Total	15,641	100.0%

How often do you worry about how much money your family has?	Number	Percentage (95% CI)
Never	5,264	34.1% (33.3%-34.8%)
Some of the time	6,124	39.7% (38.9%-40.4%)
All of the time	4,054	26.3% (25.6%-27%)
Missing	199	
Total	15,641	100.0%

For peer review only

Supplementary Table S1c: Response to items within 'Friends and School' domain

How much do you like your school?	Number	Percentage (95% CI)
Don't like it	2,032	13.1% (12.6%-13.6%)
Like it a bit	4,691	30.2% (29.5%-31%)
Like it a lot	8,797	56.7% (55.9%-57.5%)
Missing	121	
Total	15,641	100.0%

How many friends do you have?	Number	Percentage (95% CI)
Not many	2,257	14.5% (14%-15.1%)
Some	3,647	23.5% (22.8%-24.2%)
Lots	9,623	62% (61.2%-62.7%)
Missing	114	
Total	15,641	100.0%

How often do other children bully you?	Number	Percentage (95% CI)
Never	7,276	47.3% (46.6%-48.1%)
Some of the time	6,413	41.7% (41%-42.5%)
All of the time	1,679	10.9% (10.4%-11.4%)
Missing	273	
Total	15,641	100.0%

How often are you mean to other children at school?	Number	Percentage (95% CI)
Never	10,806	70.9% (70.2%-71.6%)
Some of the time	3,645	23.9% (23.2%-24.6%)
All of the time	794	5.2% (4.9%-5.6%)
Missing	396	
Total	15,641	100.0%

How often do you feel left out by other children?	Number	Percentage (95% CI)
Never	5,598	36.2% (35.5%-37%)
Some of the time	8,129	52.6% (51.8%-53.4%)
All of the time	1,729	11.2% (10.7%-11.7%)
Missing	185	
Total	15,641	100.0%

For peer review only

Supplementary Table S1d: Response to items within 'Subjective Wellbeing' domain

How often do you feel happy?	Number	Percentage (95% CI)
Never	585	3.8% (3.5%-4.1%)
Some of the time	8,306	54.2% (53.5%-55%)
All of the time	6,420	41.9% (41.2%-42.7%)
Missing	330	
Total	15,641	100.0%

How often do you feel sad?	Number	Percentage (95% CI)
Never	3,837	24.9% (24.2%-25.6%)
Some of the time	10,715	69.5% (68.8%-70.2%)
All of the time	863	5.6% (5.2%-6%)
Missing	226	
Total	15,641	100.0%

How often are you ill or unwell?	Number	Percentage (95% CI)
Never	2,216	14.4% (13.8%-14.9%)
Some of the time	11,966	77.6% (76.9%-78.2%)
All of the time	1,247	8.1% (7.7%-8.5%)
Missing	212	
Total	15,641	100.0%

Keep worries to self	Number	Percentage (95% CI)
No	10,655	68.8% (68.1%-69.5%)
Yes	4,828	31.2% (30.5%-31.9%)
Missing	158	
Total	15,641	100.0%

When I find something really hard I can work out what to do next	Number	Percentage (95% CI)
Never	1,299	8.6% (8.1%-9%)
Some of the time	9,429	62.3% (61.5%-63.1%)
All of the time	4,409	29.1% (28.4%-29.9%)
Missing	504	
Total	15,641	100.0%

For peer review only

Table S2: Percentage primary school children with zero, one, or more than one vulnerability in each domain of wellbeing

Home, family, relationships	Number	Percentage (95% CI)
None	8,085	55.9% (55%-56.7%)
One	4,902	33.9% (33.1%-34.6%)
More than one	1,489	10.3% (9.8%-10.8%)
Missing	1,165	
Total	15,641	100.0%

Material resources	Number	Percentage (95% CI)
None	6,331	48.3% (47.5%-49.2%)
One	4,763	36.4% (35.5%-37.2%)
More than one	2,008	15.3% (14.7%-16%)
Missing	2,539	
Total	15,641	100.0%

Friends and school	Number	Percentage (95% CI)
None	5,204	35% (34.3%-35.8%)
One	6,189	41.7% (40.9%-42.5%)
More than one	3,455	23.3% (22.6%-24%)
Missing	793	
Total	15,641	100.0%

Subjective wellbeing	Number	Percentage (95% CI)
None	8,312	57.4% (56.6%-58.2%)
One	4,676	32.3% (31.5%-33.1%)
More than one	1,496	10.3% (9.8%-10.8%)
Missing	1,157	
Total	15,641	100.0%

For peer review only

Supplementary Table S3: Associations of gender with vulnerabilities in child wellbeing domains

Supplementary Table S3a – Home, family, family relations

	**Family never get along well together** (N = 15,263 non-missing)		**No garden where they can play** (N = 15,393 non-missing)		**No park near home where they can play with friends** (N = 15,295 non-missing)		**Never plays in park** (N = 15,224 non-missing)	
**Gender**	**N**	**Percentage (95% CI)**	**N**	**Percentage (95% CI)**	**N**	**Percentage (95% CI)**	**N**	**Percentage (95% CI)**
Female (N = 7,647)	296	4.0% (3.5%-4.4%)	891	11.8% (11.1%-12.5%)	2225	11.8% (11.1%-12.5%)	456	6.1% (5.6%-6.7%)
Male (N = 7,994)	433	5.6% (5.1%-6.1%)	1145	14.6% (13.8%-15.4%)	2486	14.6% (13.8%-15.4%)	681	8.8% (8.2%-9.4%)

Supplementary Table S3b – Material resources

	**No warm winter coat** (N = 13,996 non-missing)		**Not having three meals a day** (N = 14,134 non-missing)		**No internet at home** (N = 14,296 non-missing)		**Worried about money all the time** (N = 15,442 non-missing)	
**Gender**	**N**	**Percentage (95% CI)**	**N**	**Percentage (95% CI)**	**N**	**Percentage (95% CI)**	**N**	**Percentage (95% CI)**
Female (N = 7,647)	616	8.9% (8.2%-9.6%)	983	14.0% (13.3%-14.9%)	1383	19.7% (18.8%-20.6%)	1771	23.4% (22.5%-24.4%)
Male (N = 7,994)	786	11.1% (10.4%-11.9%)	1260	17.7% (16.8%-18.6%)	1350	18.6% (17.7%-19.5%)	2283	28.9% (28.0%-30.0%)

Supplementary Table S3c – Friends and school

	Does not like school (N = 15,520 non-missing)		Not many friends (N = 15,527 non-missing)		Bullied some or all of the time (N = 15,368 non-missing)		Mean to others all the time (N = 15,245 non-missing)		Feel left out all the time (N = 15,456 non-missing)	
**Gender**	**N**	**Percentage (95% CI)**	**N**	**Percentage (95% CI)**	**N**	**Percentage (95% CI)**	**N**	**Percentage (95% CI)**	**N**	**Percentage (95% CI)**
Female (N = 7,647)	606	8.0% (7.4%-8.6%)	1269	16.7% (15.9%-17.6%)	4192	55.8% (54.7%-56.9%)	252	3.4% (3.0%-3.8%)	916	12.1% (11.4%-12.8%)
Male (N = 7,994)	1426	18.0% (17.1%-18.8%)	988	12.5% (11.8%-13.2%)	3900	49.7% (48.6%-50.8%)	542	7.0% (6.4%-7.6%)	813	10.3% (9.7%-11.0%)

Supplementary Table S3d – Subjective wellbeing

	Never happy (N = 15,331 non-missing)		Always sad (N = 15,415 non-missing)		Always ill or unwell (N = 15,429 non-missing)		Keeps worries to self (N = 15,483 non-missing)		Cannot work out what to do when things are hard (N = 15,137 non-missing)	
**Gender**	**N**	**Percentage (95% CI)**	**N**	**Percentage (95% CI)**	**N**	**Percentage (95% CI)**	**N**	**Percentage (95% CI)**	**N**	**Percentage (95% CI)**
Female (N = 7,647)	179	2.4% (2.1%-2.8%)	412	5.5% (5.0%-6.0%)	581	7.7% (7.1%-8.3%)	2166	28.5% (27.5%-29.6%)	555	7.5% (6.9%-8.1%)
Male (N = 7,994)	406	5.2% (4.7%-5.7%)	451	5.7% (5.2%-6.3%)	666	8.5% (7.9%-9.1%)	2662	33.7% (32.7%-34.8%)	744	9.6% (9.0%-10.3%)

Supplementary Table S3e: Associations of gender for each child wellbeing domain

	Home, family and family relationships (N = 14,476 non-missing)		Material resources (N = 13,102 non-missing)		Friends and school (N = 14,848 non-missing)		Subjective wellbeing (N = 14,484 non-missing)	
**Gender**	**N**	**Percentage (95% CI)**	**N**	**Percentage (95% CI)**	**N**	**Percentage (95% CI)**	**N**	**Percentage (95% CI)**
Female (N = 7,647)	7,117	41.7% (40.6%-42.9%)	6,520	48.8% (47.6%-50.0%)	7,284	65.0% (63.9%-66.1%)	7,148	39.5% (38.4%-40.7%)
Male (N = 7,994)	7,359	46.5% (45.3%-47.6%)	6,582	54.5% (53.3%-55.7%)	7,564	64.9% (63.8%-66.0%)	7,336	45.6% (44.5%-46.7%)

For peer review only

Supplementary Table S4: Associations of ethnicity with vulnerabilities in child wellbeing domains

Supplementary Table S4a – Home, family, family relations

	Family never get along well together (N = 15,263 non-missing)		No garden where they can play (N = 15,393 non-missing)		No park near home where they can play with friends (N = 15,295 non-missing)		Never plays in park (N = 15,224 non-missing)	
**Ethnicity**	**N**	**Percentage (95% CI)**	**N**	**Percentage (95% CI)**	**N**	**Percentage (95% CI)**	**N**	**Percentage (95% CI)**
Pakistani (N = 7,031)	278	4.1% (3.6%-4.6%)	837	12.1% (11.4%-12.9%)	2243	32.7% (31.6%-33.9%)	470	6.9% (6.3%-7.5%)
White British (N = 4,247)	265	6.3% (5.6%-7.1%)	562	13.4% (12.4%-14.5%)	1247	29.9% (28.6%-31.3%)	374	9.0% (8.2%-9.9%)
Mixed (N = 900)	55	6.3% (4.9%-8.1%)	134	15.0% (12.8%-17.5%)	254	28.7% (25.8%-31.8%)	75	8.5% (6.8%-10.5%)
White Other (N = 707)	24	3.5% (2.3%-5.2%)	125	18.0% (15.3%-21%)	188	27.1% (23.9%-30.6%)	46	6.7% (5.1%-8.9%)
Bangladeshi (N = 471)	18	3.9% (2.5%-6.2%)	73	15.8% (12.8%-19.5%)	154	33.6% (29.4%-38.0%)	32	6.9% (4.9%-9.6%)
Indian (N = 357)	17	4.9% (3.0%-7.7%)	64	18.1% (14.4%-22.4%)	121	34.4% (29.6%-39.5%)	25	7.2% (4.9%-10.4%)
Black/ Black British (N = 277)	5	1.8% (0.8%-4.3%)	49	18.0% (13.9%-23%)	62	22.7% (18.1%-28.1%)	19	7.0% (4.5%-10.7%)
Gypsy/ Irish traveller (N = 190)	13	7.3% (4.3%-12.2%)	28	15.2% (10.7%-21.2%)	54	29.2% (23.1%-36.1%)	18	9.9% (6.3%-15.2%)
Other (N = 425)	14	3.4% (2.0%-5.7%)	76	18.1% (14.7%-22.1%)	129	31.5% (27.2%-36.1%)	35	8.5% (6.1%-11.5%)

For peer review only

Supplementary Table S4b – Material resources

Ethnicity	No warm winter coat (N = 13,996 non-missing)		Not having three meals a day (N = 14,134 non-missing)		No internet at home (N = 14,296 non-missing)		Worried about money all the time (N = 15,442 non-missing)	
	N	Percentage (95% CI)	N	Percentage (95% CI)	N	Percentage (95% CI)	N	Percentage (95% CI)
Pakistani (N = 7,031)	609	9.8% (9.1%-10.6%)	1116	17.9% (16.9%-18.8%)	1141	18.0% (17.0%-18.9%)	2031	29.3% (28.2%-30.3%)
White British (N = 4,247)	432	11.1% (10.1%-12.1%)	470	11.9% (10.9%-12.9%)	880	22.3% (21.0%-23.6%)	912	21.6% (20.4%-22.9%)
Mixed (N = 900)	76	9.6% (7.8%-11.9%)	145	18.0% (15.5%-20.8%)	159	19.7% (17.1%-22.6%)	223	25.3% (22.5%-28.3%)
White Other (N = 707)	55	8.9% (6.9%-11.4%)	90	14.4% (11.8%-17.3%)	113	17.7% (14.9%-20.8%)	204	29.4% (26.1%-32.9%)
Bangladeshi (N = 471)	31	7.5% (5.3%-10.5%)	65	16.1% (12.8%-20.0%)	74	17.6% (14.2%-21.5%)	128	27.6% (23.7%-31.8%)
Indian (N = 357)	31	9.6% (6.8%-13.3%)	67	20.4% (16.4%-25.1%)	47	14.3% (10.9%-18.5%)	88	24.9% (20.7%-29.7%)
Black/ Black British (N = 277)	20	8.0% (5.2%-12.1%)	40	15.9% (11.9%-20.9%)	36	13.7% (10%-18.4%)	74	26.8% (21.9%-32.3%)
Gypsy/ Irish traveller (N = 190)	15	9.4% (5.7%-15%)	32	19.8% (14.3%-26.6%)	43	25.3% (19.3%-32.4%)	73	39.0% (32.3%-46.2%)
Other (N = 425)	33	8.9% (6.4%-12.2%)	56	14.9% (11.6%-18.8%)	81	21.3% (17.5%-25.7%)	101	24.4% (20.5%-28.8%)

For peer review only

Supplementary Table S4c – Friends and school

Ethnicity	N	Does not like school (N = 15,520 non-missing) Percentage (95% CI)	N	Not many friends (N = 15,527 non-missing) Percentage (95% CI)	N	Bullied some or all of the time (N = 15,368 non-missing) Percentage (95% CI)	N	Mean to others all the time (N = 15,245 non-missing) Percentage (95% CI)	N	Feel left out all the time (N = 15,456 non-missing) Percentage (95% CI)
Pakistani (N = 7,031)	889	12.7% (12.0%-13.5%)	1029	14.7% (13.9%-15.6%)	3630	52.6% (51.4%-53.7%)	400	5.8% (5.3%-6.4%)	790	11.4% (10.6%-12.1%)
White British (N = 4,247)	652	15.4% (14.4%-16.6%)	662	15.7% (14.6%-16.8%)	2253	53.8% (52.3%-55.3%)	167	4.0% (3.5%-4.7%)	486	11.6% (10.6%-12.6%)
Mixed (N = 900)	128	14.4% (12.2%-16.8%)	113	12.7% (10.6%-15%)	459	51.9% (48.6%-55.1%)	41	4.7% (3.5%-6.3%)	98	11.0% (9.1%-13.2%)
White Other (N = 707)	69	9.8% (7.8%-12.3%)	106	15.2% (12.7%-18%)	328	47.1% (43.4%-50.8%)	44	6.4% (4.8%-8.5%)	74	10.6% (8.5%-13.1%)
Bangladeshi (N = 471)	54	11.5% (8.9%-14.8%)	53	11.3% (8.7%-14.5%)	235	51.1% (46.5%-55.6%)	24	5.2% (3.5%-7.6%)	40	8.5% (6.3%-11.4%)
Indian (N = 357)	33	9.3% (6.7%-12.8%)	46	13.0% (9.9%-16.9%)	183	52.0% (46.8%-57.2%)	15	4.3% (2.6%-7.0%)	31	8.8% (6.3%-12.3%)
Black/ Black British (N = 277)	32	11.6% (8.3%-15.9%)	23	8.3% (5.6%-12.2%)	144	52.9% (47%-58.8%)	14	5.2% (3.1%-8.5%)	24	8.8% (6.0%-12.8%)
Gypsy/ Irish traveller (N = 190)	20	10.7% (7.0%-16.0%)	30	16.0% (11.4%-21.9%)	79	42.2% (35.4%-49.4%)	18	9.9% (6.3%-15.2%)	21	11.3% (7.5%-16.7%)
Other (N = 425)	53	12.6% (9.8%-16.2%)	60	14.3% (11.3%-18%)	233	56.3% (51.5%-61.0%)	24	5.8% (3.9%-8.5%)	40	9.6% (7.1%-12.8%)

For peer review only

Supplementary Table S4d – Subjective wellbeing

	Never happy (N = 15,331 non-missing)		Always sad (N = 15,415 non-missing)		Always ill or unwell (N = 15,429 non-missing)		Keeps worries to self (N = 15,483 non-missing)		Cannot work out what to do when things are hard (N = 15,137 non-missing)	
**Ethnicity**	**N**	**Percentage (95% CI)**	**N**	**Percentage (95% CI)**	**N**	**Percentage (95% CI)**	**N**	**Percentage (95% CI)**	**N**	**Percentage (95% CI)**
Pakistani (N = 7,031)	281	4.1% (3.7%-4.6%)	406	5.9% (5.3%-6.4%)	622	9% (8.3%-9.7%)	2222	31.9% (30.8%-33%)	570	8.4% (7.7%-9.1%)
White British (N = 4,247)	168	4% (3.5%-4.7%)	223	5.3% (4.7%-6%)	305	7.2% (6.5%-8.1%)	1268	30.2% (28.8%-31.6%)	372	9% (8.2%-9.9%)
Mixed (N = 900)	38	4.3% (3.2%-5.9%)	55	6.2% (4.8%-8%)	73	8.3% (6.6%-10.3%)	281	31.5% (28.6%-34.7%)	88	10.1% (8.2%-12.2%)
White Other (N = 707)	26	3.8% (2.6%-5.5%)	43	6.2% (4.6%-8.2%)	50	7.2% (5.5%-9.4%)	210	30% (26.8%-33.5%)	69	10.1% (8.1%-12.7%)
Bangladeshi (N = 471)	14	3.1% (1.8%-5.1%)	21	4.5% (3%-6.9%)	45	9.7% (7.3%-12.8%)	139	29.8% (25.8%-34.1%)	22	4.8% (3.2%-7.1%)
Indian (N = 357)	6	1.7% (0.8%-3.8%)	20	5.7% (3.7%-8.7%)	22	6.3% (4.2%-9.4%)	112	31.6% (27%-36.7%)	23	6.7% (4.5%-9.9%)
Black/ Black British (N = 277)	6	2.2% (1%-4.8%)	16	5.8% (3.6%-9.3%)	19	6.9% (4.4%-10.5%)	79	28.6% (23.6%-34.2%)	15	5.6% (3.4%-9.1%)
Gypsy/ Irish traveller (N = 190)	6	3.3% (1.5%-7.1%)	13	7% (4.1%-11.7%)	16	8.6% (5.3%-13.5%)	60	32.3% (25.9%-39.3%)	27	15.2% (10.6%-21.2%)
Other (N = 425)	14	3.4% (2%-5.6%)	23	5.5% (3.7%-8.2%)	36	8.6% (6.2%-11.7%)	135	32.5% (28.1%-37.1%)	38	9.3% (6.8%-12.5%)

Supplementary Table S5: Associations of Index of Multiple Deprivation for each child wellbeing domain

IMD 2019 Quintile (1 = most materially deprived)	Home, family and family relationships (N = 5,703 non-missing)		Material resources (N = 5,153 non-missing)		Friends and school (N = 5,869 non-missing)		Subjective wellbeing (N = 5,706 non-missing)	
	N	Percentage (95% CI)	N	Percentage (95% CI)	N	Percentage (95% CI)	N	Percentage (95% CI)
1 (N = 4,334)	1817	45.4% (43.8%-46.9%)	1907	53.5% (51.9%-55.1%)	2713	65.7% (64.2%-67.1%)	1726	43.1% (41.6%-44.6%)
2 (N = 1,303)	511	42.0% (39.2%-44.8%)	546	48.5% (45.6%-51.5%)	797	64.0% (61.3%-66.6%)	482	39.5% (36.8%-42.3%)
3 (N = 308)	103	35.5% (30.0%-41.0%)	119	43.9% (38.0%-49.8%)	182	61.1% (55.5%-66.6%)	113	39.6% (34.0%-45.3%)
4 (N = 139)	50	38.5% (30.1%-46.8%)	49	37.1% (28.9%-45.4%)	80	60.6% (52.3%-68.9%)	47	35.1% (27.0%-43.2%)
5 (N = 59)	23	39.0% (26.5%-51.4%)	19	33.9% (21.5%-46.3%)	33	56.9% (44.2%-69.6%)	19	32.8% (20.7%-44.8%)

BMJ Open

Vulnerabilities in child wellbeing among primary school children: a cross-sectional study in Bradford, UK

Journal:	*BMJ Open*
Manuscript ID	bmjopen-2021-049416.R2
Article Type:	Original research
Date Submitted by the Author:	23-Mar-2022
Complete List of Authors:	Pickett, Kate; University of York<br>Ajebon, Mildred; University of York<br>Hou, Bo; Bradford Institute for Health Research<br>Kelly, Brian; Bradford Institute for Health Research<br>Bird, Philippa K; Leeds Teaching Hospitals NHS Trust<br>Dickerson, Josie; Bradford Institute for Health Research<br>Shire, Katy; Bradford Institute for Health Research<br>McIvor, Claire; Bradford Institute for Health Research<br>Mon-Williams, Mark; University of Leeds, Psychology<br>Small, Neil; University of Bradford<br>McEachan, Rosemary; Bradford Institute for Health Research<br>Wright, John; Bradford Institute for Health Research<br>Lawlor, Deborah; University of Bristol
**Primary Subject Heading**:	Epidemiology
Secondary Subject Heading:	Public health
Keywords:	COVID-19, Community child health < PAEDIATRICS, SOCIAL MEDICINE

SCHOLARONE™
Manuscripts

I, the Submitting Author has the right to grant and does grant on behalf of all authors of the Work (as defined in the below author licence), an exclusive licence and/or a non-exclusive licence for contributions from authors who are: i) UK Crown employees; ii) where BMJ has agreed a CC-BY licence shall apply, and/or iii) in accordance with the terms applicable for US Federal Government officers or employees acting as part of their official duties; on a worldwide, perpetual, irrevocable, royalty-free basis to BMJ Publishing Group Ltd ("BMJ") its licensees and where the relevant Journal is co-owned by BMJ to the co-owners of the Journal, to publish the Work in this journal and any other BMJ products and to exploit all rights, as set out in our licence.

The Submitting Author accepts and understands that any supply made under these terms is made by BMJ to the Submitting Author unless you are acting as an employee on behalf of your employer or a postgraduate student of an affiliated institution which is paying any applicable article publishing charge ("APC") for Open Access articles. Where the Submitting Author wishes to make the Work available on an Open Access basis (and intends to pay the relevant APC), the terms of reuse of such Open Access shall be governed by a Creative Commons licence – details of these licences and which Creative Commons licence will apply to this Work are set out in our licence referred to above.

Other than as permitted in any relevant BMJ Author's Self Archiving Policies, I confirm this Work has not been accepted for publication elsewhere, is not being considered for publication elsewhere and does not duplicate material already published. I confirm all authors consent to publication of this Work and authorise the granting of this licence.

For peer review only

Vulnerabilities in child wellbeing among primary school children: a cross-sectional study in Bradford, UK

Kate E Pickett[1*](0000-0002-8066-8507), Mildred Ajebon[1](0000-0001-5353-8080), Bo Hou[2](0000-0001-5337-6560), Brian Kelly[2](0000-0003-1834-2992), Philippa K Bird[3](0000-0002-9601-7979), Josie Dickerson[2](0000-0003-0121-3406), Katy Shire[2] (0000-0002-2093-181X), Claire McIvor[2] (0000-0003-3032-8165), Mark Mon-Williams[4] (0000-0001-7595-8545), Neil Small[5] (0000-0002-4426-3596), Rosemary McEachan[2] (0000-0003-1302-6675), John Wright[2] (0000-0001-9572-7293) Deborah A Lawlor[6-8] (0000-0002-6793-2262)

***Corresponding author: kate.pickett@york.ac.uk**

Addresses:

[1]Department of Health Sciences, Seebohm Rowntree Building Area 2, University of York, Heslington, York, YO105DD

[2]Bradford Institute for Health Research, Bradford Teaching Hospitals NHS Foundation Trust, Bradford Royal Infirmary, Duckworth Lane, Bradford, BD9 6RJ, UK

[3]Leeds Teaching Hospitals NHS Foundation Trust, St. James's University Hospital, Beckett Street, Leeds, West Yorkshire, LS9 7TF

[4] School of Psychology, University of Leeds, Leeds LS2 9JT

[5] Faculty of Health Studies, University of Bradford, West Yorkshire BD7 1DP UK

[6]MRC Integrative Epidemiology Unit, University of Bristol, Oakfield House, Oakfield Grove, Bristol, BS8 2BN, UK

[7]Population Health Science, Bristol Medical School, University of Bristol, UK

[8]Bristol National Institute for Health Research Biomedical Research Centre, UK

For peer review only - http://bmjopen.bmj.com/site/about/guidelines.xhtml

ABSTRACT

Objective: To describe the prevalence of factors related to wellbeing among primary school children in a deprived multi-ethnic community in the UK.

Design and participants: Cross-sectional survey of 15,641 children aged 7-10 years in Born in Bradford's Primary School Years study: whole-classroom samples in 89 Bradford primary schools between 2016 and 2019.

Main outcome measures: Prevalence estimates by ethnicity (%, 95% CI) of single and multiple vulnerabilities in child wellbeing within and across four domains (Home, Family, Relationships; Material Resources; Friends and School; Subjective Wellbeing).

Results Only 10% of children had no vulnerabilities in any domain of wellbeing; 10% had one or more vulnerabilities in all four domains. The highest prevalence estimates were for being bullied some or all of the time (52.7%, 51.9 to 53.4%), keeping worries to oneself (31.2%, 30.5 to 31.9%), having no park near home (30.8%, 30.1 to 31.5%) and worrying all the time about how much money their family has (26.3%, 25.6 to 27%).

Boys were consistently significantly more likely than girls to report all of the vulnerabilities in the Home, Family and Family Relationships domain, and the majority of indicators in the other domains, and in all domains except Friends and School, boys were significantly more likely to have at least one vulnerability. Girls were significantly more likely to report not having many friends (16.7%, 95% CI: 15.9 to 17.6% vs. 12.5%, 95% CI: 11.8 to 13.2%), being bullied some or all of the time (55.8%, 95% CI: 54.7 to 56.9% vs. 49.7%, 95% CI: 48.6 to 50.8%) and feeling left out all the time (12.1%, 95% CI: 11.4 to 12.8%) vs. (10.3%, 95% CI: 9.7 to 11.0%).

Variations in vulnerabilities by ethnicity were complex, with children in Black, Asian and Minority Ethnic groups sometimes reporting more vulnerabilities and sometimes fewer than White British children. For example, compared to children of Pakistani heritage, White British children were more likely to say that their family never gets along well (6.3%,5.6 to 7.1% vs. 4.1%,3.6 to 4.6%) and to have no access to the internet at home (22.3%,21 to 23.6% vs. 18%,17 to 18.9%). Children with Pakistani heritage were more likely than White British

For peer review only - http://bmjopen.bmj.com/site/about/guidelines.xhtml

children to say they had no park near their home where they can play with friends (32.7%,31.6 to 33.9% vs. 29.9%,28.6 to 31.3%), to report not having three meals a day (17.9%,16.9 to 18.8% vs. 11.9%,10.9 to 12.9%) and to worry all the time about how much money their families have (29.3%,28.2 to 30.3%) vs. (21.6%,20.4 to 22.9%). Gypsy/Irish Traveller children were less likely than White British children to say they were bullied some or all of the time (42.2%,35.4 to 49.4% vs. 53.8%,52.3 to 55.3%), but more likely to say they were mean to others all the time (9.9%,6.3 to 15.2% vs. 4%,3.5 to 4.7%) and can never work out what to do when things are hard (15.2%,10.6 to 21.2% vs. 9%, 8.2 to 9.9%).

We considered six vulnerabilities to be of particular concern during the Covid-19 pandemic and associated national and local lockdowns: family never gets along well together; no garden where child can play; no nearby park where they can play; not having three meals a day; no internet at home; worried about money all the time. Pre-pandemic, 37.4% (36.6 to 38.3%) of Bradford children had one of these vulnerabilities and a further 29.6% (28.9 to 30.4%) had more than one.

Conclusions

Although most primary school children aged 7-10 in our study had good levels of wellbeing on most indicators across multiple domains, fewer than 10% had no vulnerabilities at all, a worrying 10% had at least one vulnerability in all the four domains we studied and two thirds had vulnerabilities of particular concern during the Covid-19 lockdowns.

KEYWORDS

Children; subjective wellbeing; poverty; health inequalities; ethnicity; social determinants of health; cohorts; Born in Bradford; Covid-19

Summary

Strengths and limitations of this study

- A uniquely large study of contemporary child wellbeing in the UK with a high response rate and including a broad group of ethnicities, generalisable to other UK cities with high levels of ethnic diversity and deprivation
- Subjective measures of a wide range of experiences capture how children perceive their lives
- A descriptive study of prevalence of vulnerabilities in wellbeing, unable to examine causes

For peer review only

Introduction

In 2007, the UK ranked bottom of 21 countries in UNICEF's first report on child wellbeing in rich countries,[1] and from 2009 through 2019 the Good Childhood inquiry[2] and subsequent reports from the Children's Society[3] found increasing levels of unhappiness and mental distress among children and young people in the UK. The State of the Nation 2019 report on children and young people's wellbeing suggested a need to "understand wellbeing across different groups" and to "use a range of measures to understand their experience."[4]

UK research suggests that wellbeing declines as children and young people grow older[4] but little is known about wellbeing in primary school aged children in the UK as previous studies, including the Good Childhood surveys, Understanding Society and HeadStart, included children over 10 years old only. The impact of ethnicity on child wellbeing at any age is unclear and most previous UK studies have been unable to examine wellbeing across a wide range of ethnicities.[4]

The global Covid-19 pandemic and restrictions, such as lockdowns that closed schools, heightened concerns about child wellbeing across the world. The prevalence of pre-existing child vulnerabilities and risk factors that could potentially require mitigation and targeted intervention during and after the pandemic has been a concern, particularly in poor, ethnically diverse urban settings.[5]

To provide evidence to meet these knowledge gaps, we examined child wellbeing among primary school children, aged 7 to 10 years, in a city in the North of England. Bradford is the fifth largest metropolitan district in England with one of the youngest and most ethnically diverse populations.[6] Bradford also has some of the highest levels of poverty and ill-health in England; almost a quarter of Bradford children live in poverty and 24% are obese at age 10/11.[7] The aim of this paper is to describe the prevalence of vulnerabilities in child wellbeing by ethnicity and sex in a deprived community. Whilst this research was undertaken in the city of Bradford and has been used to support local policy during the Covid-19 pandemic,[8] we believe the results have wider relevance for deprived multi-ethnic urban settings.

Methods

Setting and Participants

For peer review only - http://bmjopen.bmj.com/site/about/guidelines.xhtml

Born in Bradford (BiB) is a cohort study of 13,500 children born between 2007-2011.[9 10] Between 2016 and 2019, BiB administered the Primary School Years study as a cross-sectional survey of wellbeing of children aged 7-10 years in 89 Bradford schools . We surveyed whole classrooms, including children previously recruited to the ongoing BiB cohort study as well as non-BiB children. The study protocol and detailed methods of school recruitment are described elsewhere[11 12] and the SROBE statement can be found in Supplementary Materials: 1. Schools were given information sheets and opt-out consent forms to give to parents of children in eligible year groups ahead of the school visit; one school asked for opt-in consent to be used and this was accommodated. Fewer than 5% of parents opted out of the study.

Design and Procedures

Ethical approval was obtained from the NHS Health Research Authority's Yorkshire and the Humber - Bradford Leeds Research Ethics Committee (reference: 16/YH/0062) on the 24th March 2016.

The wellbeing survey ("Me and My Life") was developed for this study (see Supplementary Materials: 2), drawing on questions asked of children in the Millennium Cohort Study,[13] the International Survey of Children's Wellbeing[14] and the Avon Longitudinal Study of Parents and Children.[15] The survey aimed to assess children's wellbeing in multiple domains, including: happiness, health, material wellbeing, relationships with family and friends, school experience, neighbourhood, aspirations and acculturation. 'Think aloud' testing with a small group of children was used to check understanding and face validity. During administration, whole classrooms completed the survey at the same time, supported by three research facilitators.

Schools provided class lists, including date of birth, sex and ethnicity. Child ethnicity is reported by parents on registration with a school. The ethnicity information provided from school records contained 192 categories. These were re-coded into 18 broad groups in line with the 2011 census categorisation of ethnic groups in the UK and for the analyses presented here collapsed into 10 categories: Pakistani, Bangladeshi, Indian, Black/Black British, White British, Mixed, Gypsy/Irish Traveller, Other White, Other, and Unknown. Where ethnicity was missing it was possible to supplement, for BiB children only, with data held by BiB, as BiB requests regular data updates of child data from the Local Authority (n=311).

For peer review only - http://bmjopen.bmj.com/site/about/guidelines.xhtml

To assess vulnerabilities in wellbeing, we grouped questions within four domains: (1) Home, Family and Family relationships, (2) Material Resources, (3) Friends and School, (4) Subjective (self-reported) Wellbeing. Within each domain, we defined 'vulnerabilities' on (a) the basis of research literature showing these to be well-established childhood risk factors for long-term health, wellbeing, educational attainment, and social mobility and (b) subsequent consultation with the vulnerabilities workstream of the Bradford Institute for Health Research Covid-19 Scientific Advisory Group (BIHR C-SAG) (https://www.bradfordresearch.nhs.uk/vulnerable-groups/). The risk factors categorised as vulnerabilities include, by domain:

- **Home, Family, Family Relationships**: family never gets along well together, no garden where child can play, no nearby park where they can play, never plays in park

- **Material Resources**: no warm winter coat, not having three meals a day, no internet at home, worries about money all the time

- **Friends and School**: doesn't like school, not many friends, bullied some or all of the time, mean to others all the time, feel left out all the time

- **Subjective Wellbeing**: never happy, always sad, ill/unwell all of the time, keeps worries to self, can never work out what to do when things are hard

<u>Data Analysis</u>

Socio-demographics and the prevalence of vulnerabilities in child wellbeing are presented as counts and/or percentages with 95% confidence intervals, for all children and by sex and ethnicity. We also describe the number and percent of children with multiple vulnerabilities within and across domains of wellbeing by sex and ethnicity. Children with missing data for any answer are excluded from the relevant analyses and number of missing reported in figures and tables.

Results

Figure 1 shows the number of schools and children included in the study.

Table 1: Socio-demographic characteristics of 15,641 Bradford primary school children

	**Number**	**Percent**

**Sex**		
Female	7647	48.9%
Male	7994	51.1%
**Age (years)**		
6	2	<0.01%
7	5264	33.7%
8	6901	44.1%
9	3175	20.3%
10	293	1.9%
11	1	<0.01%
12	3	<0.01%
13	2	<0.01%
Missing		
**Ethnicity**		
Pakistani	7031	45.0%
Bangladeshi	471	3.0%
Indian	357	2.3%
Black/Black British	277	1.8%
White British	4247	27.1%
Mixed	900	5.7%
Gypsy/Irish Traveller	190	1.2%
White Other	707	4.5%
Other	425	2.7%
Unknown	1036	6.6%

Table 1 shows the socio-demographics of the 15,641 survey respondents. Figure 2 presents the prevalence of each vulnerability across all domains for all children (see also Supplementary Materials, Table S1); Figure 3 presents the percentage of all children who have zero, one and more than one vulnerability within each domain (see also Supplementary Materials, Table S2).

Although the prevalence of vulnerability varied by item and domain, within each domain there is an item with notably high prevalence, with more than one in four children reporting vulnerability: no park near home (30.8%, 95% CI: 30.1-31.5%); worries about money all the

For peer review only - http://bmjopen.bmj.com/site/about/guidelines.xhtml

time (26.3%, 95% CI: 25.6-27.0%); bullied some or all of the time (52.7%, 95% CI: 51.9-53.4%); and keeping worries to self (31.2%, 95% CI: 30.5-31.9%). We considered six vulnerabilities in child wellbeing would be of particular concern during lockdowns and school closures associated with the Covid-19 pandemic: family never gets along well together; no garden where child can play; no nearby park where they can play; not having three meals a day; no internet at home; worried about money all the time. Before the pandemic 37.4% (36.6-38.3%) of children had one of these vulnerabilities and a further 29.6% (28.9-30.4%) had more than one.

Although the majority of children had no vulnerabilities in the Home, Family, Relationships domain (55.9%, 95% CI: 55.0-56.7%) and the Subjective Wellbeing domain (57.4%, 95% CI: 56.6-58.2%), the majority of children had one or more vulnerabilities in the Material Resources, and Friends and School domains. Only 10% (add 95% CI) of children had no vulnerabilities in any domain of wellbeing. Figure 4 is a Venn diagram that shows how vulnerabilities overlap across domains. 1,494 (10%) children had no vulnerabilities. Each oval in the Venn diagram includes all the children who had one or more vulnerabilities within that domain; so, for example, 2% of children had vulnerabilities in Home, Family, Relationships *and* Subjective Wellbeing but not in other domains. 1,519 children (10%) had one or more vulnerabilities in all four domains.

Variation by sex

Boys were consistently significantly more likely to report all of the vulnerabilities in the Home, Family and Family Relationships domain, although the sex differences were not large (see Supplementary Materials: Table 3 for full results, including 95% confidence intervals, for all domains by sex). There were no sex differences in having access to the internet at home, but boys were significantly more likely to report not having a winter coat (11.1%, 95% CI: 10.4-11.9% vs 8.9%, 95% CI: 8.2-9.6%), not having three meals a day (17.7%, 95% CI: 16.8-18.6%) vs 14.0%, 95% CI: 13.3-14.9%) and worrying about money all the time (28.9%, 95% CI: 28.0-30.0%) vs. (23.4%, 95% CI: 22.5-24.4%). Within the Friends and School domain, boys were significantly more likely to not like school (18.0%, 95% CI: 17.1-18.8% vs. 8%, 95% CI: 7.4-8.6%) and to be mean to others all the time (7.0%, 95% CI: 6.4-7.6% vs. 3.4%, 95% CI: 3.0-3.8%), while girls were significantly more likely to report not having many friends (16.7%, 95% CI: 15.9-17.6% vs. 12.5%, 95% CI: 11.8-13.2%), being bullied some or all of the time (55.8%, 95% CI: 54.7-56.9% vs. 49.7%, 95% CI: 48.6-50.8%) and

For peer review only - http://bmjopen.bmj.com/site/about/guidelines.xhtml

feeling left out all the time (12.1%, 95% CI: 11.4-12.8% vs. 10.3%, 95% CI: 9.7-11.0%). Within the Subjective Wellbeing domain there were no sex differences in being always sad or always ill or unwell, but boys were significantly likely to report never being happy (5.2%, 95% CI: 4.7-5.7% vs. 2.4%, 95% CI: 2.1-2.8%), keeping worries to themselves (33.7%, 95% CI: 32.7-34.8% vs. 28.5%, 95% CI: 27.5-29.6%) and not being about to work out what to do when things are hard (9.6%, 95% CI: 9.0-10.3% vs. 7.5%, 95% CI: 6.9-8.1%). In all domains except for Friends and School, boys were significantly more likely to have at least one vulnerability, compared to girls.

Variation by ethnicity

Figures 5-8 shows the percentages (and 95% confidence intervals) of primary school children with at least one vulnerability within each of the domains of child wellbeing, by ethnicity. Full results are given in Supplementary Materials, Tables S4a-4d.

Home, Family, Relationships (Figure 5)

Compared to White British children, Pakistani, White Other and Black/Black British children were significantly less likely to say their family never get along well together. White Other and Other children were significantly more likely to not have a garden where they can play, compared to White British children. Compared to White British children, Pakistani children were significantly more likely, and Black/Black British children significantly less likely to have no park near home where they can play with friends. Compared to White British children, Pakistani children were significantly less likely to say they never play in a park.

Material Resources (Figure 6)

There were no significant ethnic differences in the proportion of children who did not have a warm winter coat. Compared to White British children, Pakistani, Indian and Gypsy/Traveller children, as well as children of Mixed ethnicity were significantly more likely to report not having three meals a day. Pakistani, White Other, Indian and Black/Black British children were significantly less likely to report having no internet at home than White British children. Compared to White British children, Pakistani, White Other, Bangladeshi and Gypsy/Irish Traveller children were significantly more likely to worry all the time about how much money their families have.

Friends and School (Figure 7)

For peer review only - http://bmjopen.bmj.com/site/about/guidelines.xhtml

Pakistani, White Other and Indian children were significantly less likely to report not liking school than White British children, and Bangladeshi and Black/British were significantly less likely to report not having many friends. Compared to White British children, White Other and Gypsy/Irish Traveller children were significantly less likely to say they were bullied some or all of the time, and Pakistani children and Gypsy/Irish traveller children were significantly more likely to say they were mean to others all the time. There were no significant ethnic differences in the proportion of children who felt left out by others all the time.

Subjective Wellbeing (Figure 8)

There were no significant ethnic differences in the proportion of children who said they were never happy or were always sad or who said they keep worries to themselves. Compared to White British children, only Pakistani children were significantly more likely to say they were always ill or unwell. Compared to White British children, Bangladeshi children were significantly less likely, and Gypsy/Irish traveller children more likely, to say they cannot work out what to do when things are hard.

For peer review only - http://bmjopen.bmj.com/site/about/guidelines.xhtml

Discussion

Statement of Principal Findings

Although most primary school children aged 7-10 in Bradford had good levels of wellbeing on most indicators across multiple domains, fewer than 10% had no vulnerabilities at all, and a worrying 10% had more than one vulnerability in all the four domains we studied. Variations in vulnerabilities by ethnicity were complex, with children in Black, Asian and Minority Ethnic groups sometimes reporting more vulnerabilities and sometimes fewer than White British children. We found that boys reported significantly more vulnerabilities for all indicators in the Home, Family and Family Relationships domain, as well as for the majority of indicators in the Material Resources, Friends and School, and Subjective Wellbeing domains. This contrasts with the finding of few consistent sex differences in wellbeing and a trend towards worse wellbeing in girls in the UK State of the Nation 2019: Children and Young People's Wellbeing report,[4] and the general picture in the research literature of worse wellbeing for adolescent girls [16 17] and inconsistent sex differences among adults.[18 19] These differences may be related to the younger age of our sample compared to many other studies which focus on secondary school pupils and adolescents. Future Born in Bradford data collection will enable analysis of whether these sex differences in wellbeing persist after children transition to secondary school. Our findings highlight a need for greater attention to boys' wellbeing among primary age children in both research and local practice. There is evidence of beneficial effects of male-targeted health promotion interventions to improve wellbeing among male adolescents,[20] however the evidence base on interventions to support wellbeing among primary school aged boys remains limited. This finding also suggests that worse wellbeing among adolescent girls might not be inevitable if prevention efforts were started before secondary education.

We found complex variations in vulnerabilities by ethnicity, with children in Black, Asian and Minority Ethnic groups sometimes reporting more vulnerabilities and sometimes fewer than White British children. For example, compared to children of Pakistani heritage, White British children were more likely to say that their family never gets along well and to have no access to the internet at home, whereas children with Pakistani heritage were more likely than White British children to say they had no park near their home where they can play with friends, to report not having three meals a day and to worry all the time about how much money their families have. The UK State of the Nation 2019: Children and Young

For peer review only - http://bmjopen.bmj.com/site/about/guidelines.xhtml

People's Wellbeing report drew attention to the importance of "*moving beyond the average*" in understanding wellbeing but reported "no discernible differences in wellbeing by…ethnicity" whilst recognising that small sample sizes might have obscured differences and reporting statistics only for five broadly defined ethnic groups.[4] We provide a detailed description of wellbeing for nine ethnic groups, so that nuances in ethnic variation can be better understood, including for smaller ethnic groups. For example, we found that Gypsy/Irish Traveller children were less likely than White British children to say they were bullied some or all of the time, but more likely to say they were mean to others, can never work out what to do when things are hard, worry all the time about how much money their family has, and not have three meals a day.

Strengths and weaknesses of the study

To our knowledge, ours is the only large contemporary study of wellbeing in primary school aged children in England, with sufficient numbers of children belonging to some of the UK's smaller Black, Asian and Minority Ethnic communities to be able to describe their wellbeing. Our approach to school recruitment, opt-out consent and whole classroom testing, along with Born in Bradford's longstanding trusting relationships with schools and communities ensured a very high response rate. With coverage of three quarters of primary schools and a response rate of 88% we do not believe that our findings are materially affected by selection or information bias with regards to ethnicity. In line with contemporary epidemiological good practice, we did not estimate multivariable models that included ethnicity as an independent variable to avoid difficulties inherent in interpreting the ethnicity coefficient in models controlling for other variables.[21] However, in line with current UK social science practice, we believe that the description of ethnic differences is appropriate in social policy oriented literature.[22] Although our study is based in a single city, it is likely to be generalizable to other UK cities with high levels of ethnic diversity and deprivation.

The fact that our measures of wellbeing are self-reported by children is both a strength and a potential weakness. It has been argued that, for adults and children alike, the subjective experience of quality of life matters, and that, as feelings have a demonstrable objective neuropsychological reality and are correlated with objective health, social and economic outcomes, they are worthy of measurement and inference.[23 24] Clearly, our measures of vulnerability not only include things that can only be measured subjectively, such as

For peer review only - http://bmjopen.bmj.com/site/about/guidelines.xhtml

happiness or feeling left out, but also things that arguably would be better measured objectively, e.g., having access to a garden or three meals a day. However, it is possible that even for measures such as these, children's perceptions that they lack material resources may be important even if they actually have the resources.

Our data collection objectives and approach to categorising vulnerabilities in wellbeing was to capture a broad range of children's circumstances and experiences that might underpin variation in developing life trajectories. This is distinct from the Adverse Childhood Experiences (ACEs) approach[25] and the definition and measurement of extreme child vulnerabilities collated and reported by the Children's Commissioner for England (https://www.childrenscommissioner.gov.uk/chldrn/), both of which measure factors that put children at immediate, as well as long-term, risk of harm. Our objective was to collect data on more common wellbeing outcomes that are also risk factors with known long-term consequences for health and wellbeing, to better understand children's subjective experiences across the whole population, and to measure wellbeing itself rather than social indicators, such as determinants of wellbeing, or well-becoming.[24]In our school-based whole classroom surveys, time constraints and study design prevented data collection and analyses that would have allowed us to go beyond description and investigate the causes of vulnerabilities in child wellbeing. For BiB children only, we examined associations between neighbourhood area deprivation (Index of Multiple Deprivation) and vulnerabilities. We found no significant associations for any domain except for more vulnerabilities in Material Resources at higher levels of deprivation, and as lack of material resources is definitional for deprivation do not report those analyses here (See Supplementary Materials, Table S5). We plan future data collection with this sample to allow us to examine how vulnerabilities at primary school ages shape trajectories of wellbeing across time.

Implications for providers of services to children and policymakers

Implications for services and schools: Our findings showed the importance of friendships and school environments as key stressors for children. Schools are well placed to identify concerns and intervene early to support children's wellbeing. There is a growing evidence base on 'what works' to support wellbeing and mental health in schools,[26] however most studies have focused on adolescent age groups.

For peer review only - http://bmjopen.bmj.com/site/about/guidelines.xhtml

Wellbeing is also increasingly recognised as a central aim and focus of education, for example through the Well Schools Movement (https://www.well-school.org/), and the roll-out of mental health support in schools.[27] As visible, trusted spaces in communities, schools can also be employed to engage and connect with families and enhance access to support where required.[28] Although all schools work hard to create safe and supportive environments for children, the high levels of bullying and social isolation that we report suggests that more needs to be done to make this a reality for all children, and to address inequalities in wellbeing by ethnicity. This could be facilitated through better resourcing for schools to support wellbeing and create safe and supportive environments.

From a public health perspective, multi-sectoral approaches are required to integrate access to support across services, with schools at the centre. Pathways need to facilitate identification and provision of support across services and sectors covering the domains we have identified as key concerns for children's wellbeing – including from schools to local welfare services, voluntary sector mental health support, health care and early help. In the context of limited evidence on interventions to reduce inequalities in children's wellbeing by sex and ethnicity, local monitoring and evaluation needs to disaggregate data to understand how services can mitigate inequalities.

Implications for national and local policymakers: Our research also highlights the importance of structural factors, from household finances and resources to green space. Action on the wider and structural determinants of wellbeing is critical to reduce inequalities in children's wellbeing, including the wide-ranging inequalities by ethnicity. Bradford is the 13th most deprived local authority in England (Indices of deprivation 2019), and many of the schools studied fall within the most deprived neighbourhoods in England. National policies to prevent children growing up in poverty and deprived neighbourhoods include uplifts to child benefit and abolishment of the two-child limit, improved access to free school meals and improvements to the National Living Wage.[29] There is a need to direct further resources to schools, especially in deprived areas, to facilitate work to support wellbeing and reduce inequalities. At a local level, local authorities, schools and other services can take action to ensure families can access advice services, limit the costs of the school day and school holidays, and facilitate access to high quality green space.

For peer review only - http://bmjopen.bmj.com/site/about/guidelines.xhtml

Implications in the context of the Covid-19 pandemic: Our findings suggest that, prior to the Covid-19 pandemic, a substantial number of children were experiencing sub-optimal wellbeing with potential lifelong consequences, with implications for all providers of services to children, as well as local and national policymakers. As the economic impact of the pandemic is increasing child poverty, income insecurity, food and housing insecurity, parental mental health challenges and family violence, the need for attention to the identification and amelioration of vulnerability is heightened.[30] The International Society for Social Pediatrics and Child Health has called for the adoption of "a Child Rights Based Approach (CRBA) to respond to the Covid-19 pandemic, and to advance a future in which the health, development and well-being of children and youth are prioritised with explicit strategies to reduce health inequities and advance social justice" (https://www.issop.org/2020/06/01/issop-covid-19-declaration/). Such an approach can underpin and inform services and policy making at all levels.

As the population was encouraged to 'lockdown' at home and schools closed for almost all children (and attendance was very low for those who were eligible to go to school) for varying periods, the response to the pandemic will have affected children's wellbeing (as well as their educational trajectories[31]) differently, depending on their circumstances and context. Children who found school a nurturing refuge from difficult home situations will have suffered from school shutdowns, whereas others who found school a challenging environment may have been happy to be at home for a prolonged period; most children have probably experienced both positive and negative impacts.[32 33] In addition, our subsequent research has confirmed that the high prevalence of children with no access to the internet at home and no access to a garden or park has meant that many children have at times been unable to study[32] or be physically active.[34]

Locally, our research team has engaged with our local authority, public health, NHS and voluntary sector colleagues to identify principles to mitigate the impact of the pandemic on vulnerable groups (https://www.bradfordresearch.nhs.uk/wp-content/uploads/2020/04/CSAG-Briefing-Paper-Vulnerable-Groups-and-Recovery-230420.pdf). We have proposed additional support for children and young people in post-pandemic recovery planning, including assessing the impact of decision making on children,

For peer review only - http://bmjopen.bmj.com/site/about/guidelines.xhtml

prioritising children's needs, providing extra support for vulnerable children, and monitoring impacts on children's lives and wellbeing.

Conclusions and implications for public health

Among primary school children aged 7-10 in a deprived Northern city in the UK, fewer than 10% had no vulnerabilities in Home, Family and Family Relationships, Material Resources, Friends and School and Subjective Wellbeing, while 10% had more than one vulnerability in all of these domains. These children are at risk of poor health, social and economic trajectories throughout their lives. For public health, our findings highlight the emergence of inequalities in child wellbeing at an early age, and the need for early intervention to improve children's lives and reduce impacts later in life. Multi-sectoral approaches are required to address the wide range of domains, across health, schools and wider local services, with integrated pathways to identify and support children. Inequalities in child wellbeing could be mitigated by national policy and local practice focused at structural factors, such as poverty eradication, and school-based approaches to identify and support wellbeing using both universal approaches and targeted support for the most vulnerable.

For peer review only

Figure Captions

Figure 1: Recruitment of schools, Born in Bradford cohort members and non-Born in Bradford children to the BiB Primary School Years wellbeing survey

Figure 2: Percent of primary school children with each vulnerability across domains of wellbeing

Figure 3: Percent of primary school children with zero, one or more than vulnerability in each domain of wellbeing

Figure 4: Percent of children with vulnerabilities within and across domains

Figure 5: Percent of primary school children with at least one vulnerability in the Home, Family and Family Relationships domain

Figure 6: Percent of primary school children with at least one vulnerability in the Material Resources domain

Figure 7: Percent of primary school children with at least one vulnerability in the Friends and School domain

Figure 8: Percent of primary school children with at least one vulnerability in the Subjective Wellbeing domain

For peer review only

For peer review only - http://bmjopen.bmj.com/site/about/guidelines.xhtml

References

1. UNICEF Innocenti Research Centre. Child poverty in perspective: An overview of child well-being in rich countries. Florence: Innocenti Report Card, 2007

2. Layard R, Dunn J. A Good Childhood: Searching for values in a competitive age. London: Penguin 2009.

3. The Children's Society. The Good Childhood Report. London: The Children's Society, 2012-2019

4. Department for Education. State of the Nation 2019: Children and Young People's Wellbeing. London: Department for Education, UK Government, 2019

5. International Society for Social Pediatrics and Child Health. Declaration: Advancing Health Equity and Social Justice in Response to COVID-19: https://www.issop.org/cmdownloads/issop_covid-declaration/, 2020

6. Office for National Statistics. 2011 Census: Key Statistics and Quick Statistics for Local Authorities in the United Kingdom. 2013, 2013

7. Public Health England. Public Health Outcomes Framework: Yorkshire and Humber 2020 [Available from: https://tinyurl.com/y2y8yo84 accessed 25 July 2020.

8. Bradford Institute for Health Research. Overview: BIHR Covid-19 Scientific Advisory Group 2020 [Available from: https://www.bradfordresearch.nhs.uk/wp-content/uploads/2020/05/C-SAG-Overview-210520.pdf accessed 25 July 2020.

9. Raynor P, Born in Bradford Collaborative G. Born in Bradford, a cohort study of babies born in Bradford, and their parents: Protocol for the recruitment phase. *BMC public health* 2008;8(1):327.

10. Wright J, Small N, Raynor P, et al. Cohort profile: The Born in Bradford multi-ethnic family cohort study. *International journal of epidemiology* 2012 doi: 10.1093/ije/dys112 [published Online First: 2012/10/16]

For peer review only - http://bmjopen.bmj.com/site/about/guidelines.xhtml

11. Bird PK, McEachan RRC, Mon-Williams M, et al. Growing up in Bradford: protocol for the age 7-11 follow up of the Born in Bradford birth cohort. *BMC public health* 2019;19(1):939. doi: 10.1186/s12889-019-7222-2 [published Online First: 2019/07/14]

12. Shire KA, Mon-Williams M, Hill LJB, et al. The Born in Bradford Primary School Years project: In school assessment of 7-11 year old's cognitive and sensorimotor abilities. *Wellcome Open Research* In preparation

13. National Centre for Social Research. Child of the new century: age 7 survey: http://doc.ukdataservice.ac.uk/doc/6411/mrdoc/pdf/mcs4_child_self_completion.pdf, 2008

14. International Survey of Children's Wellbeing. Children's Worlds: ISCWeB: https://isciweb.org/wp-content/uploads/2019/12/Childrens-Worlds_Q08_100713_Website.pdf, 2017

15. Avon Longitudinal Study of Parents and Children. Child completed questionnaires: http://www.bristol.ac.uk/alspac/researchers/our-data/questionnaires/child-completed-questionnaires/, Accessed: 30/12/2020

16. Public Health England. Wellbeing of adolescent girls: An analysis of data from the Health Behaviour in School-aged Children (HBSC) survey for England, 2014 London: Public Health England, 2017

17. Deighton J, Lereya T, Patalay P, et al. Mental health problems in young people aged 11-14: Results from the first HeadStart annual survey of 30,000 children. London: CAMHS Press 2018.

18. Batz C, & Tay, L. . Gender differences in subjective well-being. In: E. Diener SO, & L. Tay, ed. Handbook of well-being. Salt Lake City, UT: DEF Publishers 2018.

19. Batz-Barbarich C, Tay L, Kuykendall L, et al. A Meta-Analysis of Gender Differences in Subjective Well-Being: Estimating Effect Sizes and Associations With Gender Inequality. *Psychological Science* 2018;29(9):1491-503. doi: 10.1177/0956797618774796

20. Gwyther K, Swann R, Casey K, et al. Developing young men’s wellbeing through community and school-based programs: A systematic review. *PloS one* 2019;14(5):e0216955. doi: 10.1371/journal.pone.0216955

For peer review only - http://bmjopen.bmj.com/site/about/guidelines.xhtml

21. VanderWeele TJ, Robinson WR. On the causal interpretation of race in regressions adjusting for confounding and mediating variables. Epidemiology 2014;25(4):473-84.

22. Salway S, Barley R, Allmark P, Gerrish K, Higginbottom G, Ellison G. Ethnic diversity and inequality: ethical and scientific rigour in social research. York: Joseph Rowntree Foundation; 2011.

23. Bradshaw J. The well-being of children in the UK. Bristol: Policy Press 2016.

24. Ben-Arieh A. Developing indicators for child well-being in a changing context. In: McAuley R, Rose W, eds. Child well-being: Understanding children's lives. London: Jessica Kingley Publishers 2010:129-42.

25. Felitti VJ, Anda RF, Nordenberg D, et al. Relationship of childhood abuse and household dysfunction to many of the leading causes of death in adults. The Adverse Childhood Experiences (ACE) Study. *Am J Prev Med* 1998;14(4):245-58. doi: 10.1016/s0749-3797(98)00017-8 [published Online First: 1998/06/23]

26. Clarke A, Sorgenfrei M, Mulcahy J, et al. Adolescent mental health: A systematic review on the effectiveness of school-based interventions: Early Intervention Foundation, 2021.https://www.eif.org.uk/report/adolescent-mental-health-a-systematic-review-on-the-effectiveness-of-school-based-interventions

27. Department for Education. Promoting and supporting mental health and wellbeing in schools and colleges: UK Government, 2021.https://www.gov.uk/guidance/mental-health-and-wellbeing-support-in-schools-and-colleges

28. Mon-Williams M, Ayadurai C, Bond C, et al. Schools and education. In: Pickett K. T-RD, et al, ed. The Child of the North: Building a fairer future after COVID-19: Northern Health Science Alliance and N8 Research Partnership 2021. https://www.thenhsa.co.uk/app/uploads/2021/12/Child-of-the-North-Report-FINAL.pdf

29. Bradshaw J, Wickham S, Alexiou A, et al. Child poverty, inequality and deprivation. In: Pickett K. T-RD, et al, ed. The Child of the North: Building a fairer future after COVID-19: Northern Health Science Alliance and N8 Research Partnership 2021. https://www.thenhsa.co.uk/app/uploads/2021/12/Child-of-the-North-Report-FINAL.pdf

For peer review only - http://bmjopen.bmj.com/site/about/guidelines.xhtml

30. Dickerson J KB, Lockyer B, Bridges S, Cartwright C, Willan K, Shire, K, Crossley K, Sheldon TA, Lawlor DA, Wright J, McEachan RRC, Pickett KE. Experiences of lockdown during the Covid-19 pandemic: descriptive findings from a survey of families in the Born in Bradford study [version 2; peer review: 2 approved]. *Wellcome Open Research* 2020;5(228):(https://doi.org/10.12688/wellcomeopenres.6317.1).

31. Waterman A, Shire K. The impact of Covid-19 on learning and wellbeing. Bradford: Centre for Applied Educational Research, 2020.https://caer.org.uk/wp-content/uploads/2020/11/CAERschoolsurveyCovid.pdf

32. Dickerson J, Hou B, Lockyer B, et al. 'I think I am falling behind': Findings of the Born in Bradford Covid19 Children Survey. Bradford: Bradford Institute for Health Research, 2020.https://www.bradfordresearch.nhs.uk/wp-content/uploads/2020/11/BiB-Covid-19-Child-Briefing-Summary-V1.0-August-2020.pdf

33. Moss R EC, Lockyer B, Iqbal H, Kelly B, McIvor C, Crossley K, Sheard L, Islam S, Razaq R, Zarate M, Anser Z, Bridges S, Smith H, Lawlor D, Willan K, Rahman A, McEachan R, Wright J, Bryant M, Pickett K, Dickerson J. "Falling behind at school": Summary of the free-text findings from Phase 2 (Oct – Dec 2020) of the Born in Bradford Covid-19 Child Survey. Bradford: Bradford Institute for Health Research, 2021.https://www.bradfordresearch.nhs.uk/wp-content/uploads/2021/10/Ph-2-Child-Qual-v0.8-FINAL.pdf

34. Bingham DD, Daly-Smith A, Hall J, et al. Covid-19 lockdown: Ethnic differences in children's self-reported physical activity and the importance of leaving the home environment; a longitudinal and cross-sectional study from the Born in Bradford birth cohort study. *International Journal of Behavioral Nutrition and Physical Activity* 2021;18(1):117. doi: 10.1186/s12966-021-01183-y

For peer review only - http://bmjopen.bmj.com/site/about/guidelines.xhtml

Ethics approval

Ethical approval has been obtained from the National Health Service Health Research Authority Yorkshire and the Humber (Bradford Leeds) Research Ethics Committee (reference: 16/YH/0062).

Transparency statement

Kate Pickett (the manuscript's guarantor) affirms that the manuscript is an honest, accurate, and transparent account of the study being reported; that no important aspects of the study have been omitted; and that any discrepancies from the study as originally planned have been explained.

Competing interests

No authors declare competing interests.

Contributions

KEP, DAL, JWr, RM, MM-W, NS obtained funding for the study, and with PKB and JD conceived the research questions and analyses. BK, BH, MA, KS cleaned and curated the data, conducted analyses and contributed to the methodology. KEP drafted the first manuscript; KEP,PKB and CM revised the manuscript. All authors read and approved the final version of the manuscript.

Data Availability

Underlying data

Scientists are encouraged and able to use BiB data, which are available through a system of managed open access. The steps below describe how to apply for access to BiB data.

Before you contact BiB, please make sure you have read our Guidance for Collaborators. Our BiB Executive review proposals on a monthly basis and we will endeavour to respond to your request as soon as possible. You can find out about the different datasets which are available here. If you are unsure if we have the data that you need please contact a member of the BiB team (borninbradford@bthft.nhs.uk).

Once you have formulated your request please complete the 'Expression of Interest' form available here and send to the BiB Programme Director (rosie.mceachan@bthft.nhs.uk).

For peer review only - http://bmjopen.bmj.com/site/about/guidelines.xhtml

If your request is approved, we will ask you to sign a collaboration agreement and, if your request involves biological samples, we will ask you to complete a material transfer agreement.

Extended data

This project contains the following extended data:

- Supplementary Materials

Data are available under the terms of the Creative Commons Zero "No rights reserved" data waiver (CC0 1.0 Public domain dedication).

Acknowledgements

This study was funded by a joint grant from the UK Medical Research Council and UK Economic and Social Science Research Council (MR/N024397/1), with support from a Wellcome Trust infrastructure grant (WT101597MA) and the National Institute for Health Research under its Applied Research Collaboration Yorkshire and Humber (NIHR200166). The funders had no role in the design of the study, the collection, analysis, or interpretation of the data; the writing of the manuscript, or the decision to submit the manuscript for publication. All authors are independent of the funders, had full access to all of the data (including statistical reports and tables) in the study and can take responsibility for the integrity of the data and the accuracy of the data analysis. Views expressed in this paper are those of the authors and not necessarily those of any funder.

Born in Bradford is only possible because of the enthusiasm and commitment of the Children and Parents in BiB. We are grateful to all the participants, health professionals, schools, and researchers who have made Born in Bradford happen. Research results are disseminated through https://borninbradford.nhs.uk/our-findings/ and actively through various engagement activities to parents, children and young people and stakeholders.

For peer review only - http://bmjopen.bmj.com/site/about/guidelines.xhtml

Patient and Participant Involvement

BiB is committed to the co-production of research. Our child wellbeing survey was shared with the Parent Governors group for feedback during development, and piloted with Bradford school children.

For peer review only

For peer review only - http://bmjopen.bmj.com/site/about/guidelines.xhtml

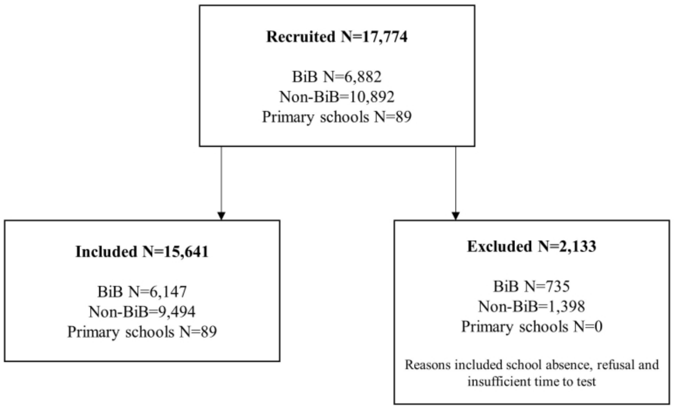

Figure 1: Recruitment of schools, Born in Bradford cohort members and non-Born in Bradford children to the BiB Primary School Years wellbeing survey

254x190mm (300 x 300 DPI)

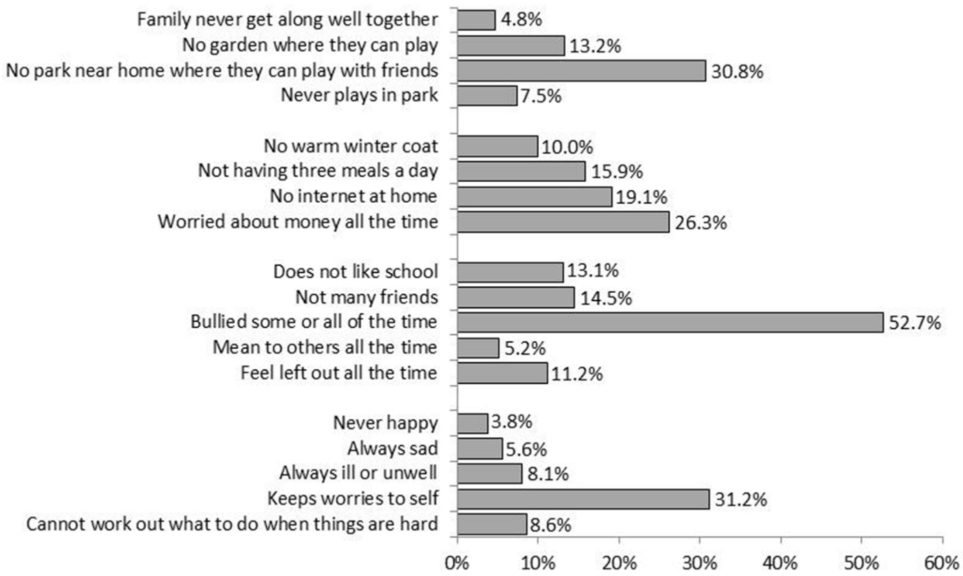

Figure 2: Percent of primary school children with each vulnerability across domains of wellbeing

254x190mm (300 x 300 DPI)

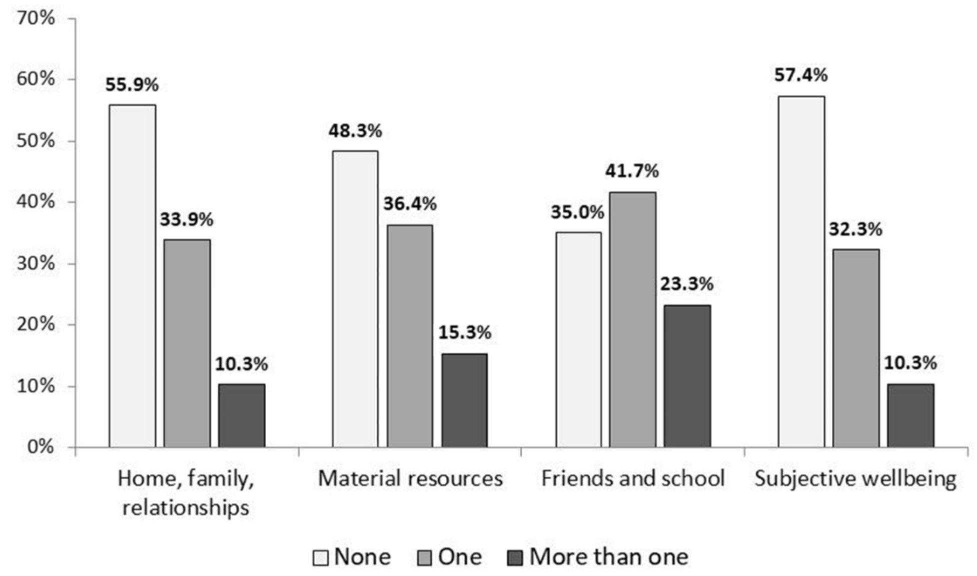

Figure 3: Percent of primary school children with zero, one or more than vulnerability in each domain of wellbeing

254x190mm (300 x 300 DPI)

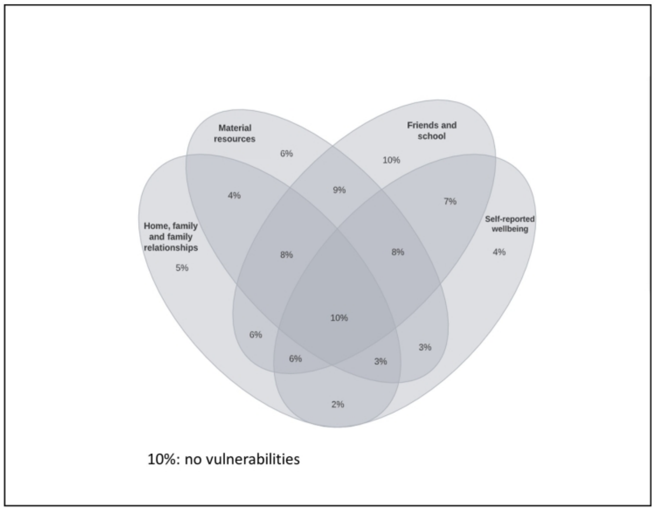

Figure 4: Percent of children with vulnerabilities within and across domains

254x190mm (300 x 300 DPI)

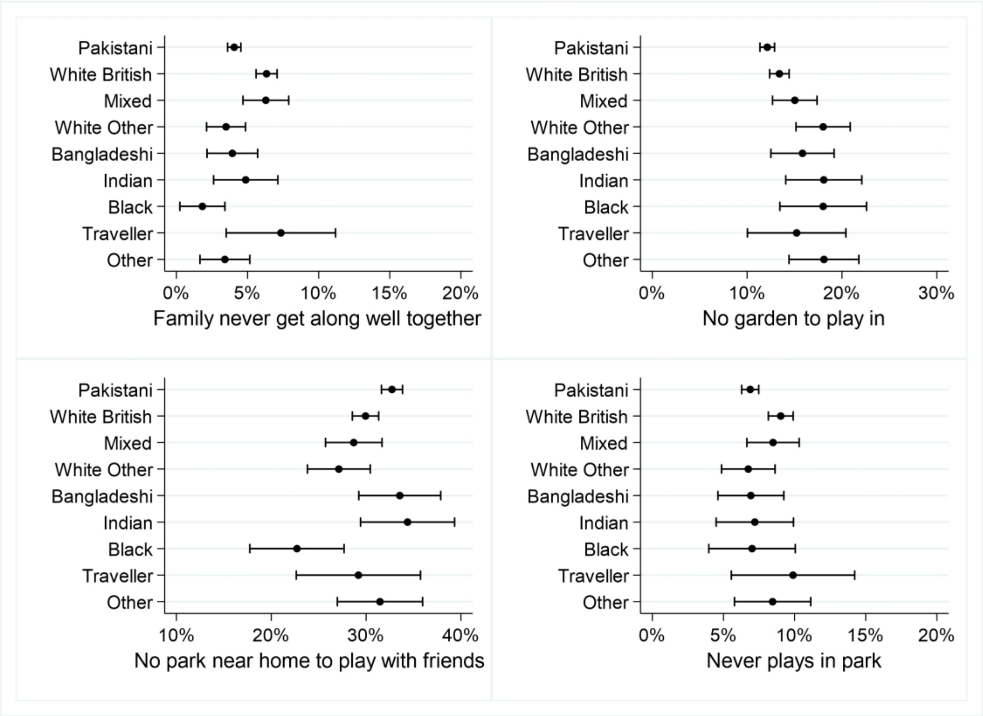

Figure 5: Percent of primary school children with at least one vulnerability in the Home, Family and Family Relationships domain

254x190mm (300 x 300 DPI)

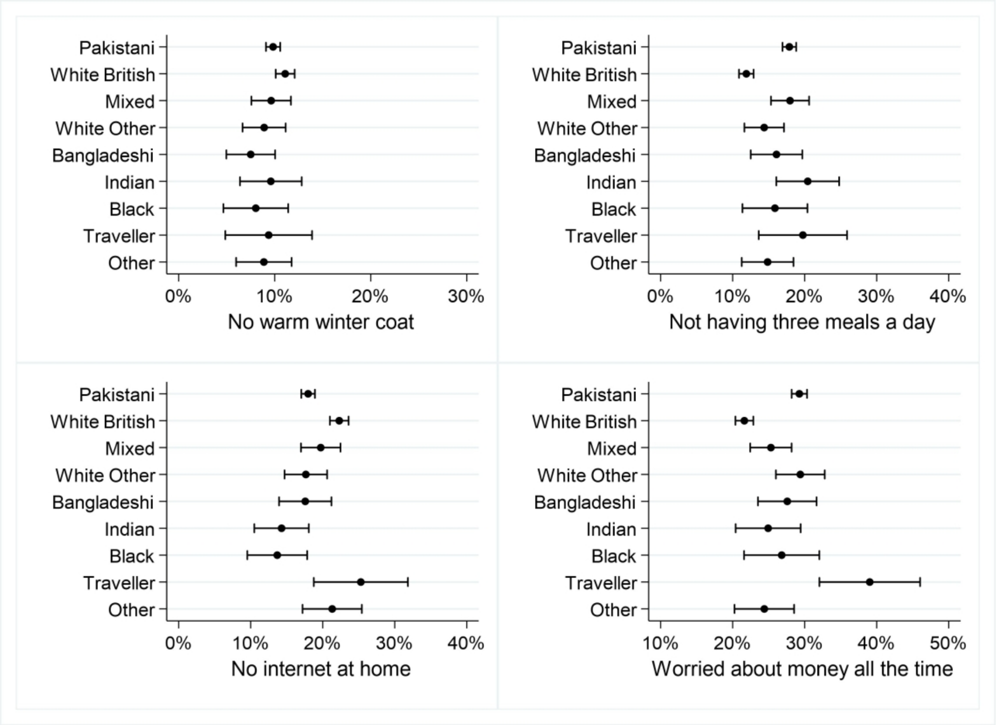

Figure 6: Percent of primary school children with at least one vulnerability in the Material Resources domain

254x190mm (300 x 300 DPI)

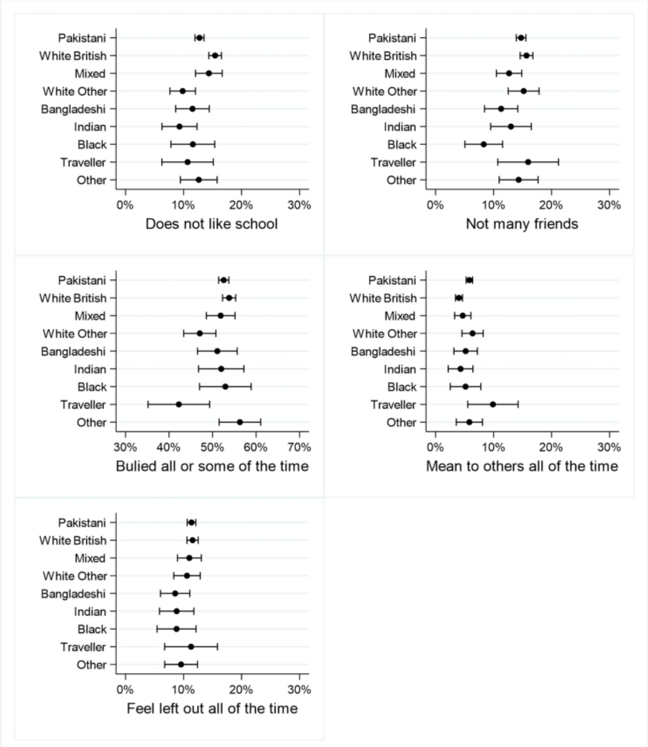

Figure 7: Percent of primary school children with at least one vulnerability in the Friends and School domain

254x190mm (300 x 300 DPI)

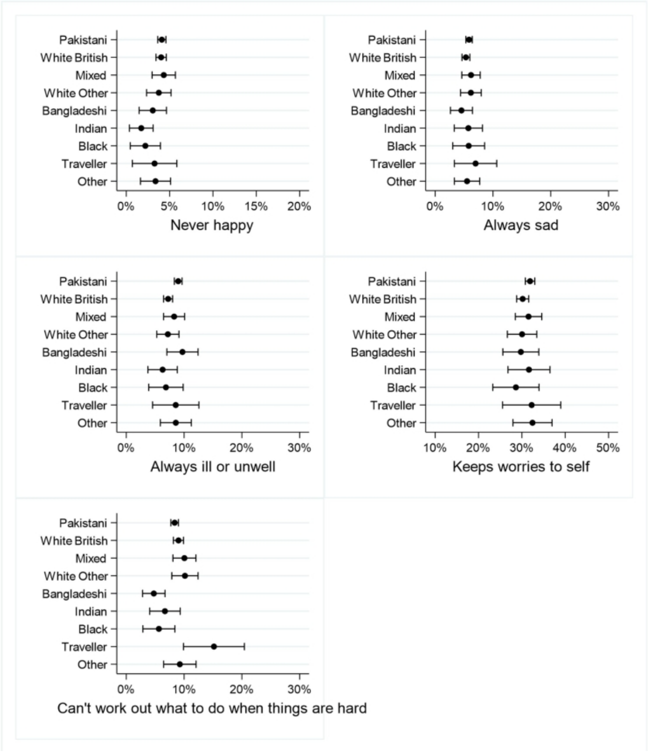

Figure 8: Percent of primary school children with at least one vulnerability in the Subjective Wellbeing domain

254x190mm (300 x 300 DPI)

Supplementary Materials: Vulnerabilities in child wellbeing among primary school children: a cross sectional study in Bradford, UK

1: STROBE Statement—Checklist of items that should be included in reports of ***cross-sectional studies***

	Item No	Recommendation
**Title and abstract**	1	(*a*) Indicate the study's design with a commonly used term in the title or the abstract SEE TITLE
		(*b*) Provide in the abstract an informative and balanced summary of what was done and what was found SEE ABSTRACT
**Introduction**		
Background/rationale	2	Explain the scientific background and rationale for the investigation being reported SEE INTRODUCTION, PAGE 4
Objectives	3	State specific objectives, including any prespecified hypotheses N/A
**Methods**		
Study design	4	Present key elements of study design early in the paper SEE METHODS, PAGES -7
Setting	5	Describe the setting, locations, and relevant dates, including periods of recruitment, exposure, follow-up, and data collection SEE SETTINGS AND PARTICIPANTS, PAGE 5
Participants	6	(*a*) Give the eligibility criteria, and the sources and methods of selection of participants SEE SETTINGS AND PARTICIPANTS, PAGE 5
Variables	7	Clearly define all outcomes, exposures, predictors, potential confounders, and effect modifiers. Give diagnostic criteria, if applicable SEE DESIGN AND PROCEDURES, PAGE 6
Data sources/ measurement	8*	For each variable of interest, give sources of data and details of methods of assessment (measurement). Describe comparability of assessment methods if there is more than one group SEE DESIGN AND PROCEDURES, PAGE 6
Bias	9	Describe any efforts to address potential sources of bias SEE FIGURE 1, PAGE 5
Study size	10	Explain how the study size was arrived at SEE SETTINGS AND PARTICIPANTS, PAGE 5
Quantitative variables	11	Explain how quantitative variables were handled in the analyses. If applicable, describe which groupings were chosen and why SEE DESIGN AND PROCEDURES, PAGE 6
Statistical methods	12	(*a*) Describe all statistical methods, including those used to

For peer review only

		control for confounding
		(*b*) Describe any methods used to examine subgroups and interactions
		(*c*) Explain how missing data were addressed
		(*d*) If applicable, describe analytical methods taking account of sampling strategy
		(<u>*e*</u>) Describe any sensitivity analyses<br>SEE DATA ANALYSIS, PAGE 7
**Results**		
Participants	13*	(a) Report numbers of individuals at each stage of study—eg numbers potentially eligible, examined for eligibility, confirmed eligible, included in the study, completing follow-up, and analysed
		(b) Give reasons for non-participation at each stage
		(c) Consider use of a flow diagram<br>SEE METHODS, FIGURE 1, PAGE 5 AND ALL RESULTS TABLES
Descriptive data	14*	(a) Give characteristics of study participants (eg demographic, clinical, social) and information on exposures and potential confounders<br>SEE TABLE 1, PAGE 8
		(b) Indicate number of participants with missing data for each variable of interest<br>SEE ALL TABLES AND FIGURES
Outcome data	15*	Report numbers of outcome events or summary measures<br>SEE FIGURE 1, PAGE 9
Main results	16	(*a*) Give unadjusted estimates and, if applicable, confounder-adjusted estimates and their precision (eg, 95% confidence interval). Make clear which confounders were adjusted for and why they were included<br>SEE ALL TABLES AND FIGURES
		(*b*) Report category boundaries when continuous variables were categorized<br>N/A
		(*c*) If relevant, consider translating estimates of relative risk into absolute risk for a meaningful time period<br>N/A
Other analyses	17	Report other analyses done—eg analyses of subgroups and interactions, and sensitivity analyses<br>SEE DISCUSSION RE: UNREPORTED ANALYSIS OF IMD, PAGE 18
**Discussion**		
Key results	18	Summarise key results with reference to study objectives<br>SEE STATEMENT OF PRINCIPAL FINDINGS, PAGE 16
Limitations	19	Discuss limitations of the study, taking into account sources of potential bias or imprecision. Discuss both direction and magnitude of any potential bias

For peer review only

		SEE STRENGTHS AND WEAKNESSES OF THE STUDY, PAGE 17
Interpretation	20	Give a cautious overall interpretation of results considering objectives, limitations, multiplicity of analyses, results from similar studies, and other relevant evidence<br>SEE IMPLICATIONS SECTION, PAGE 18
Generalisability	21	Discuss the generalisability (external validity) of the study results<br>SEE STRENGTHS AND WEAKNESSES OF THE STUDY, PAGE 17
**Other information**		
Funding	22	Give the source of funding and the role of the funders for the present study and, if applicable, for the original study on which the present article is based<br>SEE FUNDING STATEMENT

For peer review only

2. Me and My Life wellbeing survey

Me and my life

Please tick the box next to your answer

Please try to answer all the questions. There are no right or wrong answers. We want to know what you think.

Your answers will not be shown to anyone that you know (including mums and dads).

If you do not want to answer a question you can miss it out.

If you need any help, you can ask your teacher or the person who gave you this quiz.

Thank you!

For peer review only

1. Which of these would you most like to eat today?

Pizza 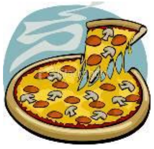 ☐

Curry 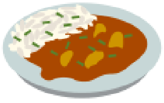 ☐

Pasta with sauce 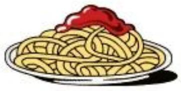 ☐

Sandwich 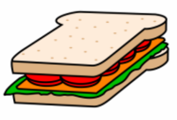 ☐

Where I live

2. How much do you like the home where you live?

I like it a lot 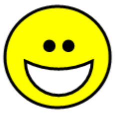 ☐

I like it a bit 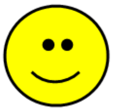 ☐

I don't like it 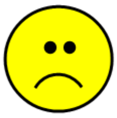 ☐

3. How often do you play in a park?

Very often ☐

Sometimes ☐

Never ☐

For peer review only

My family and the people I live with

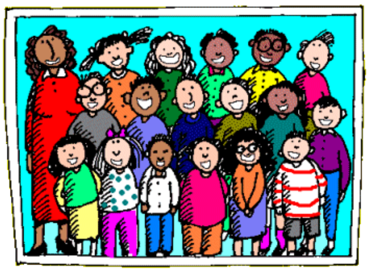

4. How often do you have fun with your family at the weekend?

All of the time ☐

Some of the time ☐

Never ☐

5. How often does your family get along well together?

All of the time ☐

Some of the time ☐

Never ☐

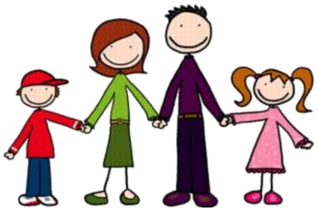

6. How often do you get along with your brothers, sisters and other children you live with?

All of the time ☐

Some of the time ☐

Never ☐

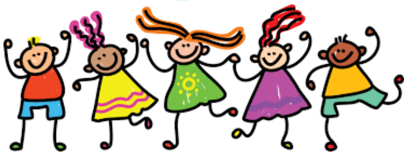

I don't have brothers or sisters or live with other children ☐

For peer review only

7. What languages do you speak with your family at home?

You can tick **more than one** answer if you like.

- English ☐
- Urdu ☐
- Mirpuri/Punjabi ☐
- Slovakian ☐
- Arabic ☐
- Bangla/Bengali ☐
- Hindko ☐
- Pushto ☐
- Other ☐

For peer review only

A bit about me

8. Here is a list of things that some children have.

Do you have each of these things?

		Yes<br>I have it	No<br>I don't have it
Some money to spend on myself	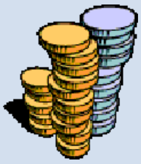	☐	☐
Clothes that you think your friends like	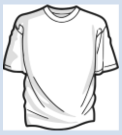	☐	☐
A warm winter coat	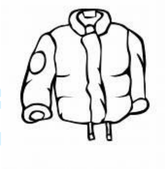	☐	☐
Your own mobile phone	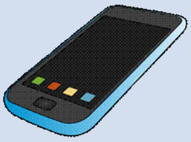	☐	☐
A computer, laptop or tablet with internet at home	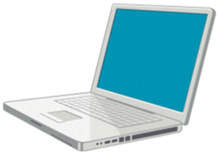	☐	☐
Three meals every day	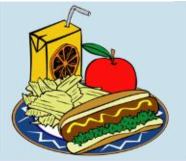	☐	☐

9. Do you have each of these things?

		Yes<br>I have it	No<br>I don't have it
**Family trips or days out at least once a month, such as to the seaside or a theme park**	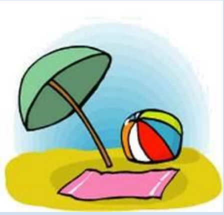	☐	☐
**At least one family holiday away from home each year**	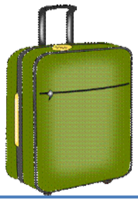	☐	☐

10. Does your home have a garden where you can play?

Yes ☐

No ☐

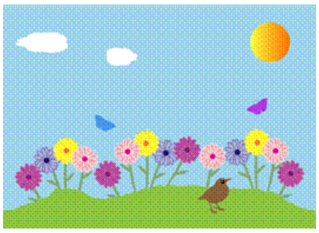

11. Does your family have a car?

Yes ☐

No ☐

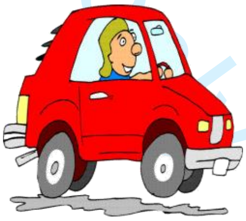

12. Do you have a park near your home where you can play with your friends?

Yes ☐

No ☐

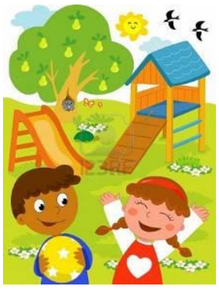

For peer review only

13. How often do you worry about how much money your family has?

All of the time ☐

Some of the time ☐

Never ☐

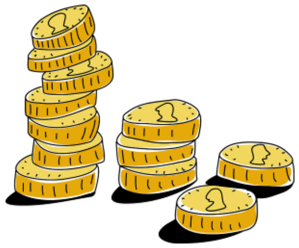

14. How often do you feel happy?

All of the time ☐

Some of the time ☐

Never ☐

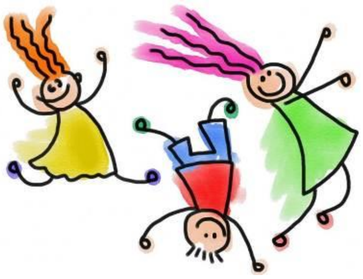

15. How often do you feel sad?

All of the time ☐

Some of the time ☐

Never ☐

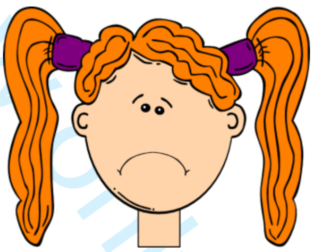

16. How often are you ill or unwell?

All of the time ☐

Some of the time ☐

Never ☐

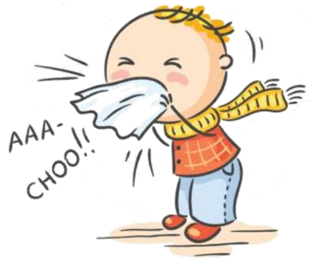

For peer review only

17. How often do you feel healthy?

All of the time ☐

Some of the time ☐

Never ☐

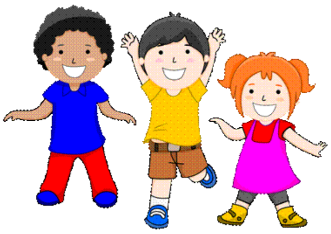

18. What do you do if you are worried about something?

You can tick **<u>more than one</u>** answer if you like.

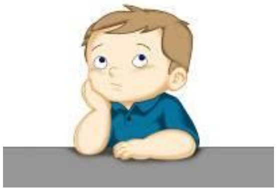

I keep it to myself ☐

I tell a friend ☐

I tell my Mum, Dad or the person who looks after me ☐

I tell a teacher ☐

19. When I find something really hard I can work out what to do next.

All of the time ☐

Some of the time ☐

Never ☐

For peer review only

My friends and other children

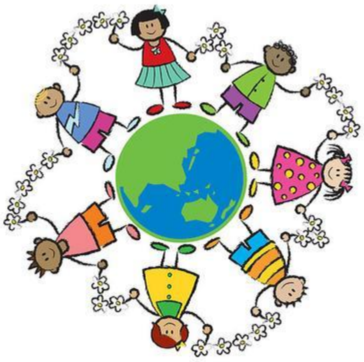

20. How many friends do you have?

Lots

Some

Not many

21. Do you have any best friends?

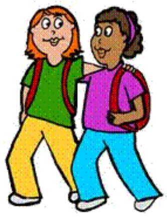

Yes

No

22. How much do you like playing with your friends?

I like it a lot 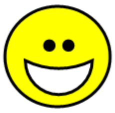

I like it a bit 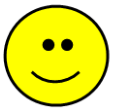

I don't like it 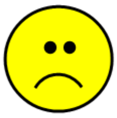

For peer review only

23. How often do other children bully you?

All of the time ☐

Some of the time ☐

Never ☐

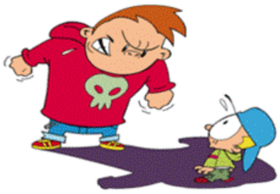

24. How often are you mean to other children at school?

All of the time ☐

Some of the time ☐

Never ☐

25. How often do you feel left out of things by other children?

All of the time ☐

Some of the time ☐

Never ☐

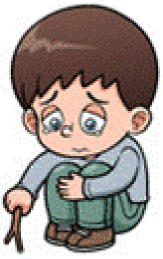

For peer review only

My school

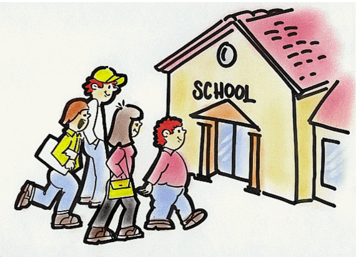

26. How much do you like school?

I like it a lot	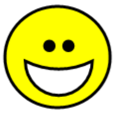	☐
I like it a bit	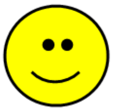	☐
I don't like it	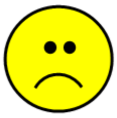	☐

After school and weekend activities

27. Do you go to any of these clubs or activities after school or at weekends?

		Yes	No
**Sports (such as football, cricket, swimming, karate)**	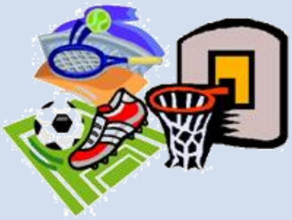	☐	☐
**Music lessons (such as choir, piano lessons)**	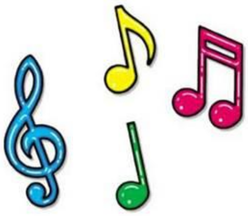	☐	☐
**Dance lessons**	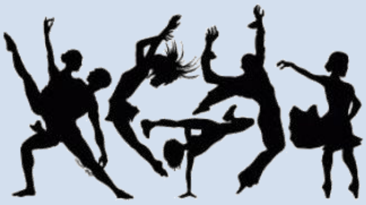	☐	☐
**Any other club (such as art, drama, film, cooking)**		☐	☐

For peer review only

Religion

28. **What is your religion?**

Christian ☐

Muslim ☐

Hindu ☐

Sikh ☐

Other religion ☐

None/No religion ☐

Don't know ☐

If you are Muslim:

Do you go to Mosque or Madrassa?

Yes ☐

No ☐

If yes: How often do you go to Mosque or Madrassa?

Most days a week ☐

Some days a week ☐

Less than once a week ☐

For peer review only

If you are Christian:

Do you go to Church or Sunday school?

Yes ☐

No ☐

If yes: How often do you go to Church or Sunday school?

Most days a week ☐

Some days a week ☐

Less than once a week ☐

If you are Hindu/Sikh:

Do you go to a Temple?

Yes ☐

No ☐

If yes: How often do you go to a Temple?

Most days a week ☐

Some days a week ☐

Less than once a week ☐

For peer review only

If you are another religion:

Do you go to a place of worship?

Yes ☐

No ☐

If yes: How often do you go to a place of worship?

Most days a week ☐

Some days a week ☐

Less than once a week ☐

For peer review only

When I grow up

29. When you grow up, would you like to be:

		Yes, I'd like it a lot	Yes, I'd like it a bit	No, not at all
A cook	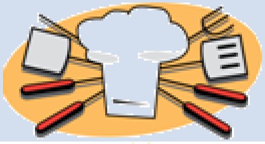	☐	☐	☐
A sports star	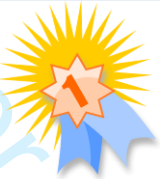	☐	☐	☐
A train driver	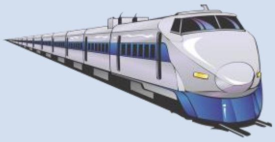	☐	☐	☐
A pop star	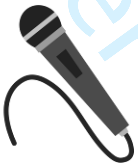	☐	☐	☐
Someone who looks after children at home	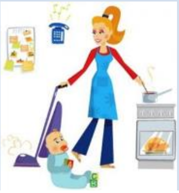	☐	☐	☐
A doctor or a nurse	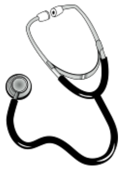	☐	☐	☐
Someone who works in a shop	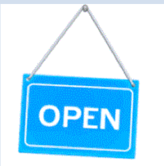	☐	☐	☐
A teacher	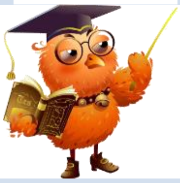	☐	☐	☐
Someone who works with computers	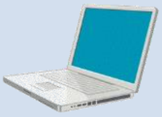	☐	☐	☐

30. What would you most like to be when you grow up?
You can write any job you like!

………

………

31. How much do you like:

		I like it a lot	I like it a bit	I don't like it/ I don't do it
**Eating Asian food, such as curry**	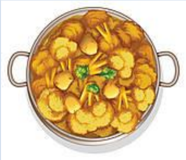	☐	☐	☐
**Eating English food, such as fish and chips**	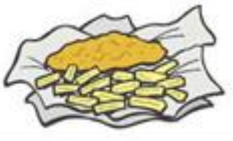	☐	☐	☐
**Wearing Asian clothes such as Salwar kameez or kurta**	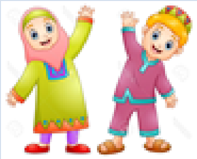	☐	☐	☐
**Wearing clothes such as jeans, t-shirts or dresses**	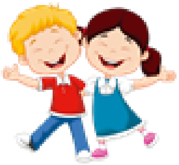	☐	☐	☐

32.

		Yes	No
Do you and your family celebrate Eid?	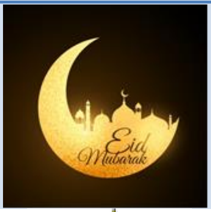	☐	☐
Do you and your family celebrate Christmas?	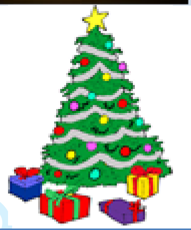	☐	☐

Thank you for your time!

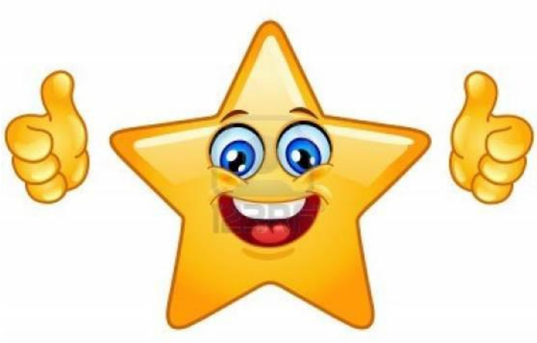

Researcher/Teacher only:
Tick if the child was assisted to complete the questionnaire. ☐

Table S1: Vulnerability across domains of wellbeing

Table S1a: Response to items within 'Home, Family and Family Relationships' domain

How often does your family get along well	Number	Percentage (95% CI)
Never	729	4.8% (4.4%-5.1%)
Some of the time	6,845	44.8% (44.1%-45.6%)
All of the time	7,689	50.4% (49.6%-51.2%)
Missing	378	
Total	15,641	100.0%

Does your home have a garden where you can play?	Number	Percentage (95% CI)
No	2,036	13.2% (12.7%-13.8%)
Yes	13,357	86.8% (86.2%-87.3%)
Missing	248	
Total	15,641	100.0%

Do you have a park near your home where you can play with your friends?	Number	Percentage (95% CI)
No	4,711	30.8% (30.1%-31.5%)
Yes	10,584	69.2% (68.5%-69.9%)
Missing	346	
Total	15,641	100.0%

How often do you play in a park?	Number	Percentage (95% CI)
Never	1,137	7.5% (7.1%-7.9%)
Sometimes	11,701	76.9% (76.2%-77.5%)
Very often	2,386	15.7% (15.1%-16.3%)
Missing	417	
Total	15,641	100.0%

For peer review only

Supplementary Table S1b: Response to items within 'Material Resources' domain

Do you have a warm winter coat?	Number	Percentage (95% CI)
No	1,402	10% (9.5%-10.5%)
Yes	12,594	90% (89.5%-90.5%)
Missing	1,645	
Total	15,641	100.0%

Do you have three meals every day?	Number	Percentage (95% CI)
No	2,243	15.9% (15.3%-16.5%)
Yes	11,891	84.1% (83.5%-84.7%)
Missing	1,507	
Total	15,641	100.0%

Do you have a computer/laptop/tablet with internet at home?	Number	Percentage (95% CI)
No	2,733	19.1% (18.5%-19.8%)
Yes	11,563	80.9% (80.2%-81.5%)
Missing	1,345	
Total	15,641	100.0%

How often do you worry about how much money your family has?	Number	Percentage (95% CI)
Never	5,264	34.1% (33.3%-34.8%)
Some of the time	6,124	39.7% (38.9%-40.4%)
All of the time	4,054	26.3% (25.6%-27%)
Missing	199	
Total	15,641	100.0%

For peer review only

Supplementary Table S1c: Response to items within 'Friends and School' domain

How much do you like your school?	Number	Percentage (95% CI)
Don't like it	2,032	13.1% (12.6%-13.6%)
Like it a bit	4,691	30.2% (29.5%-31%)
Like it a lot	8,797	56.7% (55.9%-57.5%)
Missing	121	
Total	15,641	100.0%

How many friends do you have?	Number	Percentage (95% CI)
Not many	2,257	14.5% (14%-15.1%)
Some	3,647	23.5% (22.8%-24.2%)
Lots	9,623	62% (61.2%-62.7%)
Missing	114	
Total	15,641	100.0%

How often do other children bully you?	Number	Percentage (95% CI)
Never	7,276	47.3% (46.6%-48.1%)
Some of the time	6,413	41.7% (41%-42.5%)
All of the time	1,679	10.9% (10.4%-11.4%)
Missing	273	
Total	15,641	100.0%

How often are you mean to other children at school?	Number	Percentage (95% CI)
Never	10,806	70.9% (70.2%-71.6%)
Some of the time	3,645	23.9% (23.2%-24.6%)
All of the time	794	5.2% (4.9%-5.6%)
Missing	396	
Total	15,641	100.0%

How often do you feel left out by other children?	Number	Percentage (95% CI)
Never	5,598	36.2% (35.5%-37%)
Some of the time	8,129	52.6% (51.8%-53.4%)
All of the time	1,729	11.2% (10.7%-11.7%)
Missing	185	
Total	15,641	100.0%

For peer review only

Supplementary Table S1d: Response to items within 'Subjective Wellbeing' domain

How often do you feel happy?	Number	Percentage (95% CI)
Never	585	3.8% (3.5%-4.1%)
Some of the time	8,306	54.2% (53.5%-55%)
All of the time	6,420	41.9% (41.2%-42.7%)
Missing	330	
Total	15,641	100.0%

How often do you feel sad?	Number	Percentage (95% CI)
Never	3,837	24.9% (24.2%-25.6%)
Some of the time	10,715	69.5% (68.8%-70.2%)
All of the time	863	5.6% (5.2%-6%)
Missing	226	
Total	15,641	100.0%

How often are you ill or unwell?	Number	Percentage (95% CI)
Never	2,216	14.4% (13.8%-14.9%)
Some of the time	11,966	77.6% (76.9%-78.2%)
All of the time	1,247	8.1% (7.7%-8.5%)
Missing	212	
Total	15,641	100.0%

Keep worries to self	Number	Percentage (95% CI)
No	10,655	68.8% (68.1%-69.5%)
Yes	4,828	31.2% (30.5%-31.9%)
Missing	158	
Total	15,641	100.0%

When I find something really hard I can work out what to do next	Number	Percentage (95% CI)
Never	1,299	8.6% (8.1%-9%)
Some of the time	9,429	62.3% (61.5%-63.1%)
All of the time	4,409	29.1% (28.4%-29.9%)
Missing	504	
Total	15,641	100.0%

For peer review only

Table S2: Percentage primary school children with zero, one, or more than one vulnerability in each domain of wellbeing

Home, family, relationships	Number	Percentage (95% CI)
None	8,085	55.9% (55%-56.7%)
One	4,902	33.9% (33.1%-34.6%)
More than one	1,489	10.3% (9.8%-10.8%)
Missing	1,165	
Total	15,641	100.0%

Material resources	Number	Percentage (95% CI)
None	6,331	48.3% (47.5%-49.2%)
One	4,763	36.4% (35.5%-37.2%)
More than one	2,008	15.3% (14.7%-16%)
Missing	2,539	
Total	15,641	100.0%

Friends and school	Number	Percentage (95% CI)
None	5,204	35% (34.3%-35.8%)
One	6,189	41.7% (40.9%-42.5%)
More than one	3,455	23.3% (22.6%-24%)
Missing	793	
Total	15,641	100.0%

Subjective wellbeing	Number	Percentage (95% CI)
None	8,312	57.4% (56.6%-58.2%)
One	4,676	32.3% (31.5%-33.1%)
More than one	1,496	10.3% (9.8%-10.8%)
Missing	1,157	
Total	15,641	100.0%

For peer review

Supplementary Table S3: Associations of gender with vulnerabilities in child wellbeing domains

Supplementary Table S3a – Home, family, family relations

	Family never get along well together (N = 15,263 non-missing)		No garden where they can play (N = 15,393 non-missing)		No park near home where they can play with friends (N = 15,295 non-missing)		Never plays in park (N = 15,224 non-missing)	
**Gender**	**N**	**Percentage (95% CI)**	**N**	**Percentage (95% CI)**	**N**	**Percentage (95% CI)**	**N**	**Percentage (95% CI)**
Female (N = 7,647)	296	4.0% (3.5%-4.4%)	891	11.8% (11.1%-12.5%)	2225	11.8% (11.1%-12.5%)	456	6.1% (5.6%-6.7%)
Male (N = 7,994)	433	5.6% (5.1%-6.1%)	1145	14.6% (13.8%-15.4%)	2486	14.6% (13.8%-15.4%)	681	8.8% (8.2%-9.4%)

Supplementary Table S3b – Material resources

	No warm winter coat (N = 13,996 non-missing)		Not having three meals a day (N = 14,134 non-missing)		No internet at home (N = 14,296 non-missing)		Worried about money all the time (N = 15,442 non-missing)	
**Gender**	**N**	**Percentage (95% CI)**	**N**	**Percentage (95% CI)**	**N**	**Percentage (95% CI)**	**N**	**Percentage (95% CI)**
Female (N = 7,647)	616	8.9% (8.2%-9.6%)	983	14.0% (13.3%-14.9%)	1383	19.7% (18.8%-20.6%)	1771	23.4% (22.5%-24.4%)
Male (N = 7,994)	786	11.1% (10.4%-11.9%)	1260	17.7% (16.8%-18.6%)	1350	18.6% (17.7%-19.5%)	2283	28.9% (28.0%-30.0%)

Supplementary Table S3c – Friends and school

Gender	Does not like school (N = 15,520 non-missing) N	Percentage (95% CI)	Not many friends (N = 15,527 non-missing) N	Percentage (95% CI)	Bullied some or all of the time (N = 15,368 non-missing) N	Percentage (95% CI)	Mean to others all the time (N = 15,245 non-missing) N	Percentage (95% CI)	Feel left out all the time (N = 15,456 non-missing) N	Percentage (95% CI)
Female (N = 7,647)	606	8.0% (7.4%-8.6%)	1269	16.7% (15.9%-17.6%)	4192	55.8% (54.7%-56.9%)	252	3.4% (3.0%-3.8%)	916	12.1% (11.4%-12.8%)
Male (N = 7,994)	1426	18.0% (17.1%-18.8%)	988	12.5% (11.8%-13.2%)	3900	49.7% (48.6%-50.8%)	542	7.0% (6.4%-7.6%)	813	10.3% (9.7%-11.0%)

Supplementary Table S3d – Subjective wellbeing

Gender	Never happy (N = 15,331 non-missing) N	Percentage (95% CI)	Always sad (N = 15,415 non-missing) N	Percentage (95% CI)	Always ill or unwell (N = 15,429 non-missing) N	Percentage (95% CI)	Keeps worries to self (N = 15,483 non-missing) N	Percentage (95% CI)	Cannot work out what to do when things are hard (N = 15,137 non-missing) N	Percentage (95% CI)
Female (N = 7,647)	179	2.4% (2.1%-2.8%)	412	5.5% (5.0%-6.0%)	581	7.7% (7.1%-8.3%)	2166	28.5% (27.5%-29.6%)	555	7.5% (6.9%-8.1%)
Male (N = 7,994)	406	5.2% (4.7%-5.7%)	451	5.7% (5.2%-6.3%)	666	8.5% (7.9%-9.1%)	2662	33.7% (32.7%-34.8%)	744	9.6% (9.0%-10.3%)

Supplementary Table S3e: Associations of gender for each child wellbeing domain

Gender	Home, family and family relationships (N = 14,476 non-missing) N	Percentage (95% CI)	Material resources (N = 13,102 non-missing) N	Percentage (95% CI)	Friends and school (N = 14,848 non-missing) N	Percentage (95% CI)	Subjective wellbeing (N = 14,484 non-missing) N	Percentage (95% CI)
Female (N = 7,647)	7,117	41.7% (40.6%-42.9%)	6,520	48.8% (47.6%-50.0%)	7,284	65.0% (63.9%-66.1%)	7,148	39.5% (38.4%-40.7%)
Male (N = 7,994)	7,359	46.5% (45.3%-47.6%)	6,582	54.5% (53.3%-55.7%)	7,564	64.9% (63.8%-66.0%)	7,336	45.6% (44.5%-46.7%)

For peer review only

Supplementary Table S4: Associations of ethnicity with vulnerabilities in child wellbeing domains

Supplementary Table S4a – Home, family, family relations

	Family never get along well together (N = 15,263 non-missing)		No garden where they can play (N = 15,393 non-missing)		No park near home where they can play with friends (N = 15,295 non-missing)		Never plays in park (N = 15,224 non-missing)	
**Ethnicity**	**N**	**Percentage (95% CI)**	**N**	**Percentage (95% CI)**	**N**	**Percentage (95% CI)**	**N**	**Percentage (95% CI)**
Pakistani (N = 7,031)	278	4.1% (3.6%-4.6%)	837	12.1% (11.4%-12.9%)	2243	32.7% (31.6%-33.9%)	470	6.9% (6.3%-7.5%)
White British (N = 4,247)	265	6.3% (5.6%-7.1%)	562	13.4% (12.4%-14.5%)	1247	29.9% (28.6%-31.3%)	374	9.0% (8.2%-9.9%)
Mixed (N = 900)	55	6.3% (4.9%-8.1%)	134	15.0% (12.8%-17.5%)	254	28.7% (25.8%-31.8%)	75	8.5% (6.8%-10.5%)
White Other (N = 707)	24	3.5% (2.3%-5.2%)	125	18.0% (15.3%-21%)	188	27.1% (23.9%-30.6%)	46	6.7% (5.1%-8.9%)
Bangladeshi (N = 471)	18	3.9% (2.5%-6.2%)	73	15.8% (12.8%-19.5%)	154	33.6% (29.4%-38.0%)	32	6.9% (4.9%-9.6%)
Indian (N = 357)	17	4.9% (3.0%-7.7%)	64	18.1% (14.4%-22.4%)	121	34.4% (29.6%-39.5%)	25	7.2% (4.9%-10.4%)
Black/ Black British (N = 277)	5	1.8% (0.8%-4.3%)	49	18.0% (13.9%-23%)	62	22.7% (18.1%-28.1%)	19	7.0% (4.5%-10.7%)
Gypsy/ Irish traveller (N = 190)	13	7.3% (4.3%-12.2%)	28	15.2% (10.7%-21.2%)	54	29.2% (23.1%-36.1%)	18	9.9% (6.3%-15.2%)
Other (N = 425)	14	3.4% (2.0%-5.7%)	76	18.1% (14.7%-22.1%)	129	31.5% (27.2%-36.1%)	35	8.5% (6.1%-11.5%)

For peer review only

Supplementary Table S4b – Material resources

Ethnicity	No warm winter coat (N = 13,996 non-missing) N	Percentage (95% CI)	Not having three meals a day (N = 14,134 non-missing) N	Percentage (95% CI)	No internet at home (N = 14,296 non-missing) N	Percentage (95% CI)	Worried about money all the time (N = 15,442 non-missing) N	Percentage (95% CI)
Pakistani (N = 7,031)	609	9.8% (9.1%-10.6%)	1116	17.9% (16.9%-18.8%)	1141	18.0% (17.0%-18.9%)	2031	29.3% (28.2%-30.3%)
White British (N = 4,247)	432	11.1% (10.1%-12.1%)	470	11.9% (10.9%-12.9%)	880	22.3% (21.0%-23.6%)	912	21.6% (20.4%-22.9%)
Mixed (N = 900)	76	9.6% (7.8%-11.9%)	145	18.0% (15.5%-20.8%)	159	19.7% (17.1%-22.6%)	223	25.3% (22.5%-28.3%)
White Other (N = 707)	55	8.9% (6.9%-11.4%)	90	14.4% (11.8%-17.3%)	113	17.7% (14.9%-20.8%)	204	29.4% (26.1%-32.9%)
Bangladeshi (N = 471)	31	7.5% (5.3%-10.5%)	65	16.1% (12.8%-20.0%)	74	17.6% (14.2%-21.5%)	128	27.6% (23.7%-31.8%)
Indian (N = 357)	31	9.6% (6.8%-13.3%)	67	20.4% (16.4%-25.1%)	47	14.3% (10.9%-18.5%)	88	24.9% (20.7%-29.7%)
Black/ Black British (N = 277)	20	8.0% (5.2%-12.1%)	40	15.9% (11.9%-20.9%)	36	13.7% (10%-18.4%)	74	26.8% (21.9%-32.3%)
Gypsy/ Irish traveller (N = 190)	15	9.4% (5.7%-15%)	32	19.8% (14.3%-26.6%)	43	25.3% (19.3%-32.4%)	73	39.0% (32.3%-46.2%)
Other (N = 425)	33	8.9% (6.4%-12.2%)	56	14.9% (11.6%-18.8%)	81	21.3% (17.5%-25.7%)	101	24.4% (20.5%-28.8%)

For peer review only

Supplementary Table S4c – Friends and school

	Does not like school (N = 15,520 non-missing)		Not many friends (N = 15,527 non-missing)		Bullied some or all of the time (N = 15,368 non-missing)		Mean to others all the time (N = 15,245 non-missing)		Feel left out all the time (N = 15,456 non-missing)	
**Ethnicity**	**N**	**Percentage (95% CI)**	**N**	**Percentage (95% CI)**	**N**	**Percentage (95% CI)**	**N**	**Percentage (95% CI)**	**N**	**Percentage (95% CI)**
Pakistani (N = 7,031)	889	12.7% (12.0%-13.5%)	1029	14.7% (13.9%-15.6%)	3630	52.6% (51.4%-53.7%)	400	5.8% (5.3%-6.4%)	790	11.4% (10.6%-12.1%)
White British (N = 4,247)	652	15.4% (14.4%-16.6%)	662	15.7% (14.6%-16.8%)	2253	53.8% (52.3%-55.3%)	167	4.0% (3.5%-4.7%)	486	11.6% (10.6%-12.6%)
Mixed (N = 900)	128	14.4% (12.2%-16.8%)	113	12.7% (10.6%-15%)	459	51.9% (48.6%-55.1%)	41	4.7% (3.5%-6.3%)	98	11.0% (9.1%-13.2%)
White Other (N = 707)	69	9.8% (7.8%-12.3%)	106	15.2% (12.7%-18%)	328	47.1% (43.4%-50.8%)	44	6.4% (4.8%-8.5%)	74	10.6% (8.5%-13.1%)
Bangladeshi (N = 471)	54	11.5% (8.9%-14.8%)	53	11.3% (8.7%-14.5%)	235	51.1% (46.5%-55.6%)	24	5.2% (3.5%-7.6%)	40	8.5% (6.3%-11.4%)
Indian (N = 357)	33	9.3% (6.7%-12.8%)	46	13.0% (9.9%-16.9%)	183	52.0% (46.8%-57.2%)	15	4.3% (2.6%-7.0%)	31	8.8% (6.3%-12.3%)
Black/ Black British (N = 277)	32	11.6% (8.3%-15.9%)	23	8.3% (5.6%-12.2%)	144	52.9% (47%-58.8%)	14	5.2% (3.1%-8.5%)	24	8.8% (6.0%-12.8%)
Gypsy/ Irish traveller (N = 190)	20	10.7% (7.0%-16.0%)	30	16.0% (11.4%-21.9%)	79	42.2% (35.4%-49.4%)	18	9.9% (6.3%-15.2%)	21	11.3% (7.5%-16.7%)
Other (N = 425)	53	12.6% (9.8%-16.2%)	60	14.3% (11.3%-18%)	233	56.3% (51.5%-61.0%)	24	5.8% (3.9%-8.5%)	40	9.6% (7.1%-12.8%)

For peer review only

Supplementary Table S4d – Subjective wellbeing

	Never happy (N = 15,331 non-missing)		Always sad (N = 15,415 non-missing)		Always ill or unwell (N = 15,429 non-missing)		Keeps worries to self (N = 15,483 non-missing)		Cannot work out what to do when things are hard (N = 15,137 non-missing)	
**Ethnicity**	**N**	**Percentage (95% CI)**	**N**	**Percentage (95% CI)**	**N**	**Percentage (95% CI)**	**N**	**Percentage (95% CI)**	**N**	**Percentage (95% CI)**
Pakistani (N = 7,031)	281	4.1% (3.7%-4.6%)	406	5.9% (5.3%-6.4%)	622	9% (8.3%-9.7%)	2222	31.9% (30.8%-33%)	570	8.4% (7.7%-9.1%)
White British (N = 4,247)	168	4% (3.5%-4.7%)	223	5.3% (4.7%-6%)	305	7.2% (6.5%-8.1%)	1268	30.2% (28.8%-31.6%)	372	9% (8.2%-9.9%)
Mixed (N = 900)	38	4.3% (3.2%-5.9%)	55	6.2% (4.8%-8%)	73	8.3% (6.6%-10.3%)	281	31.5% (28.6%-34.7%)	88	10.1% (8.2%-12.2%)
White Other (N = 707)	26	3.8% (2.6%-5.5%)	43	6.2% (4.6%-8.2%)	50	7.2% (5.5%-9.4%)	210	30% (26.8%-33.5%)	69	10.1% (8.1%-12.7%)
Bangladeshi (N = 471)	14	3.1% (1.8%-5.1%)	21	4.5% (3%-6.9%)	45	9.7% (7.3%-12.8%)	139	29.8% (25.8%-34.1%)	22	4.8% (3.2%-7.1%)
Indian (N = 357)	6	1.7% (0.8%-3.8%)	20	5.7% (3.7%-8.7%)	22	6.3% (4.2%-9.4%)	112	31.6% (27%-36.7%)	23	6.7% (4.5%-9.9%)
Black/ Black British (N = 277)	6	2.2% (1%-4.8%)	16	5.8% (3.6%-9.3%)	19	6.9% (4.4%-10.5%)	79	28.6% (23.6%-34.2%)	15	5.6% (3.4%-9.1%)
Gypsy/ Irish traveller (N = 190)	6	3.3% (1.5%-7.1%)	13	7% (4.1%-11.7%)	16	8.6% (5.3%-13.5%)	60	32.3% (25.9%-39.3%)	27	15.2% (10.6%-21.2%)
Other (N = 425)	14	3.4% (2%-5.6%)	23	5.5% (3.7%-8.2%)	36	8.6% (6.2%-11.7%)	135	32.5% (28.1%-37.1%)	38	9.3% (6.8%-12.5%)

For peer review only

Supplementary Table S5: Associations of Index of Multiple Deprivation for each child wellbeing domain

IMD 2019 Quintile (1 = most materially deprived)	Home, family and family relationships (N = 5,703 non-missing)		Material resources (N = 5,153 non-missing)		Friends and school (N = 5,869 non-missing)		Subjective wellbeing (N = 5,706 non-missing)	
	N	Percentage (95% CI)	N	Percentage (95% CI)	N	Percentage (95% CI)	N	Percentage (95% CI)
1 (N = 4,334)	1817	45.4% (43.8%-46.9%)	1907	53.5% (51.9%-55.1%)	2713	65.7% (64.2%-67.1%)	1726	43.1% (41.6%-44.6%)
2 (N = 1,303)	511	42.0% (39.2%-44.8%)	546	48.5% (45.6%-51.5%)	797	64.0% (61.3%-66.6%)	482	39.5% (36.8%-42.3%)
3 (N = 308)	103	35.5% (30.0%-41.0%)	119	43.9% (38.0%-49.8%)	182	61.1% (55.5%-66.6%)	113	39.6% (34.0%-45.3%)
4 (N = 139)	50	38.5% (30.1%-46.8%)	49	37.1% (28.9%-45.4%)	80	60.6% (52.3%-68.9%)	47	35.1% (27.0%-43.2%)
5 (N = 59)	23	39.0% (26.5%-51.4%)	19	33.9% (21.5%-46.3%)	33	56.9% (44.2%-69.6%)	19	32.8% (20.7%-44.8%)

For peer review only
